# Global, regional, and national epidemiology of congenital heart disease in children from 1990 to 2021

**DOI:** 10.3389/fcvm.2025.1522644

**Published:** 2025-05-16

**Authors:** Jiaoli Xu, Qinhong Li, Lili Deng, Jingxuan Xiong, Zugen Cheng, Caixia Ye

**Affiliations:** ^1^ Department of Cardiology, Kunming Children's Hospital, Kunming, Yunnan, China; ^2^ Department of Cardiology, The First Affiliated Hospital of Kunming Medical University, Kunming, China; ^3^ Department of Respiratory Medicine, Kunming Children’s Hospital, Kunming, Yunnan, China; ^4^ Department of Pediatrics, Yunyang Women and Children's Hospital (Yunyang Maternal and Child Health Hospital), Chongqing, China

**Keywords:** congenital heart disease, prevalence, mortality, DALYs—disability-adjusted life years, childhood

## Abstract

**Background:**

Congenital heart disease (CHD) is a leading cause of morbidity and mortality in children globally, with significant variations in outcomes across different regions.

**Objective:**

To provide comprehensive estimates of CHD prevalence, mortality, and disability-adjusted life years (DALYs) among children under five years old globally from 1990 to 2021.

**Methods:**

Using data from the Global Burden of Disease (GBD) study, trends in CHD prevalence, mortality, and DALYs were analyzed. Mortality estimates were generated using Cause of Death Ensemble modeling, while prevalence and DALYs were estimated using DisMod-MR 2.1. Systematic literature reviews informed the disability estimates.

**Results:**

In 2021, the global prevalence of CHD in children under five years was over 4.18 million, reflecting a 3.4% increase since 1990. CHD-associated mortality decreased by 56.2%, and DALYs declined by 55.7% from 1990 to 2021. Low and low-middle Socio-Demographic Index (SDI) regions experienced the highest prevalence and mortality rates. South Asia had the highest number of CHD cases, while Oceania had the highest mortality and DALY rates. India had the highest number of cases, while Afghanistan had the highest mortality and DALY rates.

**Conclusions:**

CHD remains a significant global health challenge, with substantial disparities in disease burden across regions. Targeted interventions are needed to improve survival and quality of life, particularly in high-burden areas.

## Introduction

It is estimated that non-communicable diseases (NCDs) are responsible for nearly three-quarters of all deaths globally each year (1). By 2048, the global annual death toll is projected to reach nearly 90 million, with 77 million of these deaths attributed to NCDs. This represents an almost 90% increase in absolute numbers compared to 2019 (2). Congenital heart disease (CHD) remains the leading cause of mortality among infants and young children due to NCDs (3). CHD is one of the most common birth defects, affecting approximately 1% of live births worldwide (4). CHD is defined as any structural abnormality of the heart or great vessels, such as a bicuspid aortic valve, present from birth, excluding cases associated with syndromic illnesses, including extra-cardiac or neurocognitive manifestations in addition to cardiac malformations (5, 6). These congenital defects can vary widely in severity, from minor conditions that may not require intervention to severe malformations necessitating complex surgeries and long-term medical care. In the fetal stage, severe types of CHD, such as complete transposition of the great arteries and hypoplastic left heart syndrome, can be detected through fetal echocardiography. These conditions typically require immediate intervention after birth (7). In contrast, milder forms of CHD, such as atrial septal defects and patent ductus arteriosus, may present symptoms during infancy or childhood (8), and in some cases, are not diagnosed until adulthood (9). Early diagnosis and management are crucial for improving outcomes and quality of life for affected individuals (10). Untreated severe CHD can lead to heart failure, growth delays, and even early death (11). Even with surgical intervention, many patients require long-term follow-up and treatment to monitor and manage potential complications, such as arrhythmias, heart failure, and infective endocarditis (12). It is estimated that 250,000 people worldwide die from CHD each year, with 1.35 million new CHD births annually. In the United States alone, there are 2.4 million individuals living with CHD (13). Despite advances in surgical treatment over the decades, CHD remains a leading cause of mortality in infants and young children (14, 15). These conditions place a significant burden on patients and their families, highlighting CHD as a critical global public health issue.

With the adoption of the new Sustainable Development Goals (SDGs) in September 2015, which aim to reduce under-five mortality rates, addressing CHD in young children has become an important target (16). According to the Global Burden of Disease (GBD) study, in 2017, 180,624 deaths due to CHD (approximately 69% of all CHD deaths across all age groups) occurred in infants under one year of age (17). Therefore, enhancing the GBD for CHD in children under five is crucial for reducing the burden of non-communicable diseases in this population. Moreover, regular reassessment of the GBD for CHD in children is vital for efforts to prevent long-term complications of CHD. To our knowledge, there has been no comprehensive analysis of epidemiological trends in CHD among children under five. This study utilizes the GBD database to analyze trends in the prevalence of CHD, CHD-related mortality, and CHD-related disability-adjusted life years (DALYs) in children under the age of five from 1990 to 2021. The interpretation of the 2021 GBD estimates is expected to contribute to improved early diagnosis and treatment of CHD, ultimately aiming to reduce health risks associated with pediatric CHD.

## Methods

### Overview and data collection

This cross-sectional study was approved by the Kunming Children's Hospital. The Ethics Committee of Kunming Children's Hospital granted a waiver of informed consent, as the study solely involved data analysis with no identifiable personal information. Data for children aged 0–5 years with CHD were sourced from the Global Health Data Exchange query tool, developed by the GBD collaborators, which provided standardized disease definitions and prevalence information.

The 2021 GBD study assessed the incidence, mortality, and morbidity of 369 diseases and injuries across 204 countries and territories from 1990 to 2021, along with their corresponding prevalence rates and uncertainty intervals (UIs) (18). This study collected data on CHD case numbers, incidence rates, CHD-related mortality, and CHD-related morbidity in children, accompanied by corresponding global, regional, and national-level rates. Notably, the GBD database does not include participant race and ethnicity data, as such classifications are not assigned during data collection.

To quantify temporal trends, linear regression was employed to calculate the Estimated Annual Percentage Change (EAPC). Additionally, data on global risk factors associated with CHD mortality in children were collected. This study adhered to the Strengthening the Reporting of Observational Studies in Epidemiology (STROBE) guidelines, ensuring rigorous reporting standards were maintained throughout.

### Sociodemographic Index

The Socio-Demographic Index (SDI) is a composite measure reflecting the development status of a country or region, based on fertility rates, educational attainment, and per capita income (19). SDI values range from 0 to 1, with higher values indicating greater socio-economic development. Previous studies have reported associations between SDI and both disease incidence and mortality. In this study, countries and geographic regions were stratified into five SDI quintiles—low, low-middle, middle, high-middle, and high—to explore the relationship between the burden of CHD in children and socio-economic development. This stratification enables a nuanced analysis of how different levels of development affect the CHD burden across various populations.

### Prevalence rate

Prevalence rate refers to the proportion or frequency of individuals within a population who have a specific disease or condition at a defined time point or within a specified period. It is usually expressed as the number of existing cases per 1,000 or 100,000 individuals in the population.

### Statistical analysis

Incidence, mortality, and prevalence rates, along with their respective ratios, are key indicators used to describe the burden of CHD in children. According to the GBD methodology, these rates are reported per 100,000 population, accompanied by 95% UIs (20). To assess the temporal trends in CHD burden, the EAPC (21) was calculated, and determined the 95% confidence intervals (CI) for EAPC using linear modeling. An EAPC with its upper limit of 95% CI entirely below zero indicates a decreasing trend in the corresponding rate, whereas an EAPC with its lower limit of 95% CI above zero signifies an increasing trend.

Furthermore, Gaussian curve analyses were employed to elucidate the relationship between EAPC, and CHD incidence in children. All analyses and graphical representations were conducted using the World Health Organization's Health Equity Assessment Toolkit and the statistical computing software R (Version 3.5.2), ensuring a robust and comprehensive exploration of the data.

## Result

### Congenital heart disease in children: global trends

#### Prevalence

In 2021, the global prevalent cases of CHD in children were 4183259 (95% UI, 3643894−4800270). From 1990 to 2021, the global prevalent cases of childhood CHD increased by 3.4% (95% UI, 2.0%−4.9%). The corresponding prevalence rate increased accordingly 652.4 (95% UI, 567.4–750.5) in 1990 to 635.6 (95% UI, 553.6–729.3) in 2021; the EAPC was 0 [95% CI, (−0.0 to 0.0)] (Supplementary Table S1).

#### Mortality

**Figure 1:**
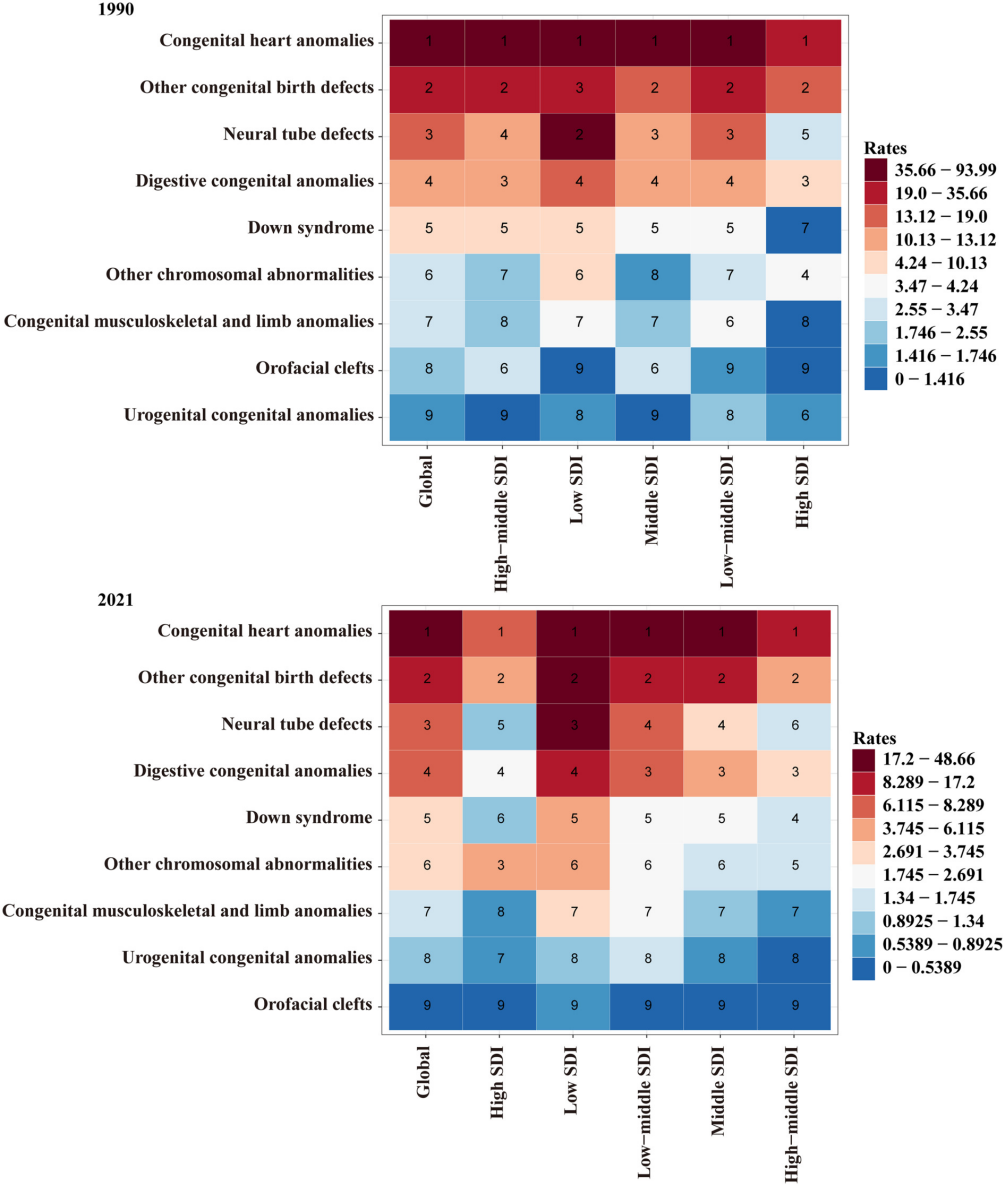
Mortality rankings for causes of death due to congenital anomalies in under-five children across global and SDI levels in 1990 and 2021.

Over the past 30 years, the global number of CHD-associated deaths in children decreased by 56.2% (466,157; 95% UI, 261,283–601,016) in 1990 vs. 204,223 (95% UI, 165,239–255,409) in 2021. Similarly, the CHD-associated death rate decreased from 75.2 (95% UI, 42.1–96.9) per 100,000 in 1990 to 31.0 (95% UI, 25.1–38.8) per 100,000 in 2021; the EAPC was −2.6 (95% CI, −2.7 to −2.5) (Table 1). Globally, CHD has consistently been the leading cause of death among children under five years old due to congenital anomalies. This was true in both 1990 and 2021, surpassing all other congenital birth defects in terms of its contribution to childhood mortality (Figure 1). The persistent dominance of CHD as the primary cause of death reflects the significant global burden of this condition, despite medical advancements in diagnosis and treatment over the past decades.

**Table 1:** Deaths of congenital heart disease in children between 1990 and 2021 at the global and regional level.

Location	1990		2021		1990–2021	
	Deaths cases	Deaths rate	Deaths cases	Deaths rate	Cases change	EAPC^a^
Global	466,156.7 (261,282.5–601,016.4)	75.2 (42.1–96.9)	204,223.0 (165,238.5–255,409.2)	31.0 (25.1–38.8)	−56.2 (−66.4 to 24.7)	−2.6 (−2.7 to 2.5)
High SDI	17,757.8 (15,242.4–19,413.3)	28.8 (24.7–31.5)	3,638.4 (2,918.6–4,432.0)	6.8 (5.4–8.2)	−79.5 (−83.9 to 73.5)	−4.3 (−4.5 to 4.2)
High-middle SDI	69,371.2 (47,871.9–86,634.3)	74.7 (51.5–93.3)	10,284.7 (8,295.2–12,492.8)	14.7 (11.8–17.8)	−85.2 (−89.2 to 76.2)	−5.3 (−5.7 to 5.0)
Middle SDI	150,753.6 (91,645.4–200,331.2)	75.2 (45.7–99.9)	41,132.4 (33,693.0–50,820.9)	23.3 (19.1–28.8)	−72.7 (−80.5 to 46.8)	−3.4 (−3.6 to 3.2)
Low-middle SDI	142,550.2 (75,050.5–194,335.9)	82.2 (43.3–112.0)	68,363.1 (52,761.3–86,318.7)	35.7 (27.5–45.1)	−52.0 (−65.4 to 5.4)	−2.3 (−2.4 to 2.1)
Low SDI	85,339.8 (29,634.4–125,904.2)	94.0 (32.6–138.7)	80,576.4 (54,063.3–109,319.6)	48.7 (32.7–66.0)	−5.6 (−28.5 to 90.8)	−1.9 (−2.0 to 1.8)
Regions						
Andean Latin America	4,811.3 (2,518.9–6,328.5)	91.1 (47.7–119.8)	2,036.5 (1,500.2–2,637.1)	33.1 (24.4–42.8)	−57.7 (−71.7 to 9.3)	−2.6 (−2.8 to 2.4)
Australasia	227.8 (207.6–254.1)	14.8 (13.5–16.5)	85.7 (60.8–109.4)	4.7 (3.3–6.0)	−62.4 (−73.7 to 51.7)	−3.3 (−3.5 to 3.1)
Caribbean	3,608.9 (2,741.7–4,587.3)	87.4 (66.4–111.0)	2,238.5 (1,402.6–3,598.2)	57.9 (36.3–93.0)	−38.0 (−57.7 to 4.3)	−1.0 (−1.2–0.7)
Central Asia	3,774.0 (3,238.6–4,291.9)	39.6 (34.0–45.1)	4,019.5 (3,098.6–5,028.5)	40.2 (31.0–50.3)	6.5 (−14.9 to 32.8)	0.6 (0.3–1.0)
Central Europe	4,440.4 (3,783.0–4,966.9)	48.6 (41.4–54.4)	615.2 (489.5–733.9)	11.0 (8.8–13.1)	−86.1 (−89.9 to 82.8)	−4.8 (−4.9 to 4.6)
Central Latin America	10,858.9 (9,476.2–12,459.1)	47.2 (41.2–54.1)	6,951.9 (5,201.9–9,050.7)	34.6 (25.9–45.0)	−36.0 (−53.0 to 13.6)	−0.7 (−0.9 to 0.5)
Central Sub-Saharan Africa	7,361.4 (2,152.7–13,430.0)	70.9 (20.7–129.3)	5,715.2 (3,496.3–9,249.1)	27.1 (16.6–43.9)	−22.4 (−44.9 to 85.5)	−2.8 (−3.0 to 2.5)
East Asia	108,856.8 (66,791.9–151,606.1)	94.0 (57.7–131.0)	12,279.7 (9,167.3–16,376.0)	15.3 (11.4–20.5)	−88.7 (−92.8 to 78.1)	−5.8 (−6.2 to 5.4)
Eastern Europe	6,567.9 (5,800.5–7,902.9)	38.1 (33.6–45.8)	1,040.6 (814.3–1,292.5)	10.3 (8.0–12.8)	−84.2 (−88.8 to 79.2)	−4.4 (−5.2 to 3.6)
Eastern Sub-Saharan Africa	27,153.2 (7,098.3–52,577.7)	75.2 (19.7–145.7)	21,035.7 (12,342.4–37,967.4)	33.0 (19.3–59.5)	−22.5 (−46.4 to 100.5)	−2.4 (−2.5 to 2.3)
High-income Asia Pacific	2,959.6 (2,363.8–3,373.3)	29.0 (23.1–33.0)	318.4 (244.4–429.6)	4.9 (3.8–6.7)	−89.2 (−91.4 to 82.7)	−5.3 (−5.5 to 5.2)
High-income North America	4,559.7 (3,890.4–4,995.0)	21.0 (17.9–23.0)	1,435.0 (1,179.9–1,791.6)	7.0 (5.8–8.7)	−68.5 (−75.0 to 57.6)	−3.0 (−3.2 to 2.8)
North Africa and Middle East	89,758.9 (39,144.9–128,430.1)	175.2 (76.4–250.7)	30,917.3 (24,328.5–38,985.8)	50.6 (39.8–63.8)	−65.6 (−75.3 to 32.7)	−3.7 (−3.9 to 3.5)
Oceania	903.8 (289.6–1,353.2)	90.0 (28.8–134.8)	1,524.0 (593.3–2,343.7)	78.8 (30.7–121.2)	68.6 (26.4–140.0)	−0.3 (−0.5 to 0.1)
South Asia	101,882.6 (64,937.6–137,510.9)	64.9 (41.4–87.6)	46,943.3 (32,435.1–68,431.0)	29.6 (20.5–43.1)	−53.9 (−69.1 to 0.8)	−2.1 (−2.3 to 2.0)
Southeast Asia	40,676.1 (18,715.6–55,616.2)	69.8 (32.1–95.4)	18,663.0 (15,082.6–23,697.2)	33.2 (26.8–42.1)	−54.1 (−66.8 to 0.8)	−2.4 (−2.5 to 2.3)
Southern Latin America	1,925.2 (1,598.6–2,280.3)	37.4 (31.1–44.3)	795.2 (637.1–989.2)	18.6 (14.9–23.1)	−58.7 (−69.0 to 46.2)	−1.9 (−2.2 to 1.6)
Southern Sub-Saharan Africa	1,686.5 (1,359.6–2,213.6)	22.6 (18.2–29.6)	1,341.6 (883.2–1,857.4)	16.7 (11.0–23.1)	−20.5 (−44.8 to 18.7)	−0.6 (−0.7 to 0.5)
Tropical Latin America	7,579.7 (6,388.1–8,846.9)	44.4 (37.4–51.8)	4,417.1 (3,505.0–5,446.4)	25.7 (20.4–31.7)	−41.7 (−56.5 to 23.3)	−1.1 (−1.5 to 0.7)
Western Europe	5,675.2 (4,868.8–6,200.1)	24.7 (21.2–27.0)	1,228.4 (959.2–1,508.5)	5.8 (4.5–7.1)	−78.4 (−83.7 to 71.6)	−4.6 (−4.8 to 4.4)
Western Sub-Saharan Africa	30,888.9 (7,574.3–47,938.8)	86.4 (21.2–134.1)	40,621.2 (22,582.0–58,735.3)	50.8 (28.2–73.5)	31.5 (−0.4 to 222.7)	−1.4 (−1.5 to 1.2)

EAPC, estimated annual percentage change; UI, uncertainty interval.; SDI, Sociodemographic Index.

^a^ EAPC is expressed as 95% CIs.

#### Disability-adjusted life years

The global number of CHD-associated DALYs in children decreased by 55.69% from 1990 to 2021 (41,970,011; 95% UI, 23,700,815–53,981,699) in 1990 vs. 18,598,827 (95% UI, 15,123,186–23,181,408) in 2021; the EAPC was −2.5 (95% CI, −2.7 to −2.4) (Supplementary Table S2).

### Congenital heart disease in children: SDI regional trends

#### Prevalence

In 2021, the low-middle SDI region reported the highest number of childhood CHD cases, with an estimated 1,240,141 cases (95% UI, 1,069,636–1,442,034). The low SDI region experienced a substantial 76.2% increase in prevalent CHD cases (95% UI, 73.2%–79.6%). However, from 1990 to 2021, the overall childhood CHD prevalence among the five SDI regions either remained stable or exhibited a marginal decline, highlighting regional disparities in the burden of CHD over time (Supplementary Table S1 and Supplementary Figures S3A,B).

#### Mortality

In 2021, all five SDI regions experienced declines in CHD-associated mortality. The low SDI region reported the highest number of CHD-related deaths, with an estimated 80,576 cases (95% UI, 54,063–109,320), while the high SDI region had the lowest mortality, with 3,638 deaths (95% UI, 2,919–4,432). Notably, the high-middle SDI region saw the most significant reduction in CHD-associated mortality, with a decrease of 85.2%. In terms of mortality rates, the low SDI region had the highest rate of CHD-related deaths, at 48.7 per 100,000 (95% UI, 32.7–66.0), whereas the high SDI region had the lowest, at 6.8 per 100,000 (95% UI, 5.4–8.2). Furthermore, the high-middle SDI region exhibited the steepest decline in CHD-associated mortality trends, with an EAPC of −5.3 (95% CI, −5.7 to −5.0) (Table 1 and Supplementary Figures S3C, D). In 1990, CHD was the leading cause of death among children under five years of age due to congenital anomalies, both in high-SDI and low-SDI regions. By 2021, CHD continued to hold this position, remaining the primary cause of mortality associated with congenital birth defects in children under five, regardless of SDI region (Figure 1).

#### Disability-adjusted life years

In 2021, the low SDI region had the highest number of CHD-associated DALYs (7,269,820; 95% UI, (4,903,173–9,820,650) with a decrease of 5.1% from 1990 to 2021. The high SDI region had the greatest decrease (77.9%) in the number of CHD-associated DALYs (Supplementary Figures S3E,F and Supplementary Table S2).

### Congenital heart disease in children: geographic regional trends

#### Prevalence

**Figure 2:**
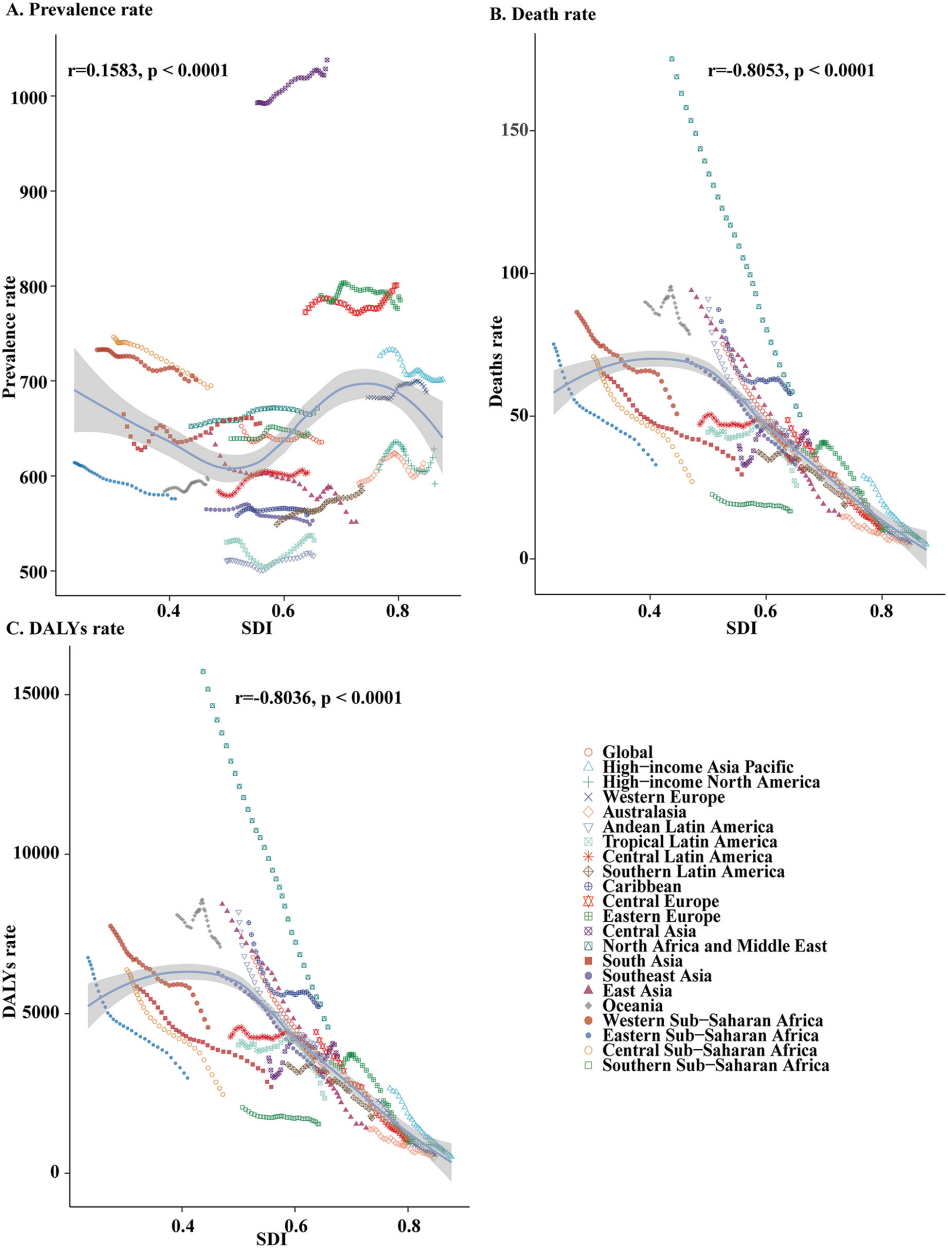
Prevalence, death, and disability-adjusted life-years rates for childhood congenital heart disease from 1990 to 2019. **(A)** Prevalence rate, **(B)** deaths rate, **(C)** DALYs rate.

Among 21 geographic regions, South Asia had the most cases of childhood CHD in 2021 (1,039,362; 95% UI, 890,150–1,216,481), whereas Australasia had the fewest (10,973; 95% UI, 9,372–12,718). The prevalence of childhood CHD was highest in Southern Latin America (1,037.9; 95% UI, 882.9–1,222.3). In contrast, the prevalence of childhood CHD was lowest in Australasia (515.9; 95% UI, 452.5–579.8). In 2021, Southern Latin America (SDI, 0.74) had the highest prevalence of childhood CHD, whereas Australasia (SDI, 0.85) had the lowest prevalence. The global SDI was 0.67 in 2021. 9 regions (e.g., Southeast Asia and the Caribbean) had higher prevalences of childhood CHD than the global mean, whereas 12 regions (e.g., Eastern Europe and Tropical Latin America) had lower prevalences than the global mean (635.6) (Supplementary Table S1 and Figure 2A).

#### Mortality

In 2021, South Asia had the highest number of childhood CHD-associated deaths (46,943; 95% UI, 32,435–68,431). Oceania had the highest childhood CHD-associated mortality rate (78.8; 95% UI, 30.7–121.2). Oceania had the smallest decrease in the childhood CHD-associated mortality rate (EAPC, −0.3; 95% CI, −0.5 to −0.1), whereas East Asia had the largest decrease (EAPC, −5.8; 95% CI, −6.2 to −5.4). In 2021, Oceania (SDI, 0.7) had the highest childhood CHD-associated mortality rate, whereas Australasia (SDI, 0.85) had the lowest mortality rate. As noted previously, the global SDI was 0.67 in 2021; 9 regions had higher childhood CHD-associated mortality rates than the global mean, whereas 12 regions had lower rates than the global mean (31) (Table 1 and Figure 2B).

#### Disability-adjusted life years

In 2021, South Asia had the highest number of childhood CHD-associated DALYs (4,287,461; 95% UI, 2,973,355–6,180,911), whereas Australasia had the lowest number (8,770; 95% UI, 6,503–10,995). Oceania had the highest DALY rate (7,092.6; 95% UI, 2,773.8–10,882.3); Australasia had the lowest DALY rate (482.9; 95% UI, 358.1–605.4). From 1990 to 2021, Oceania had the smallest decrease in the DALYs rate (EAPC, −0.3; 95% CI, −0.5 to −0.1); East Asia had the largest decrease (EAPC, −5.7; 95% CI, −6.1 to −5.3). The global SDI was 0.67 in 2021; 9 regions (e.g., Caribbean) had rates of DALYs that were higher than the global mean, whereas 12 regions (e.g., Southern Latin America) had rates that were lower than the global mean (2,825.8) (Supplementary Table S2 and Figure 2C).

### Congenital heart disease in children: national trends

#### Prevalence

**Figure 3:**
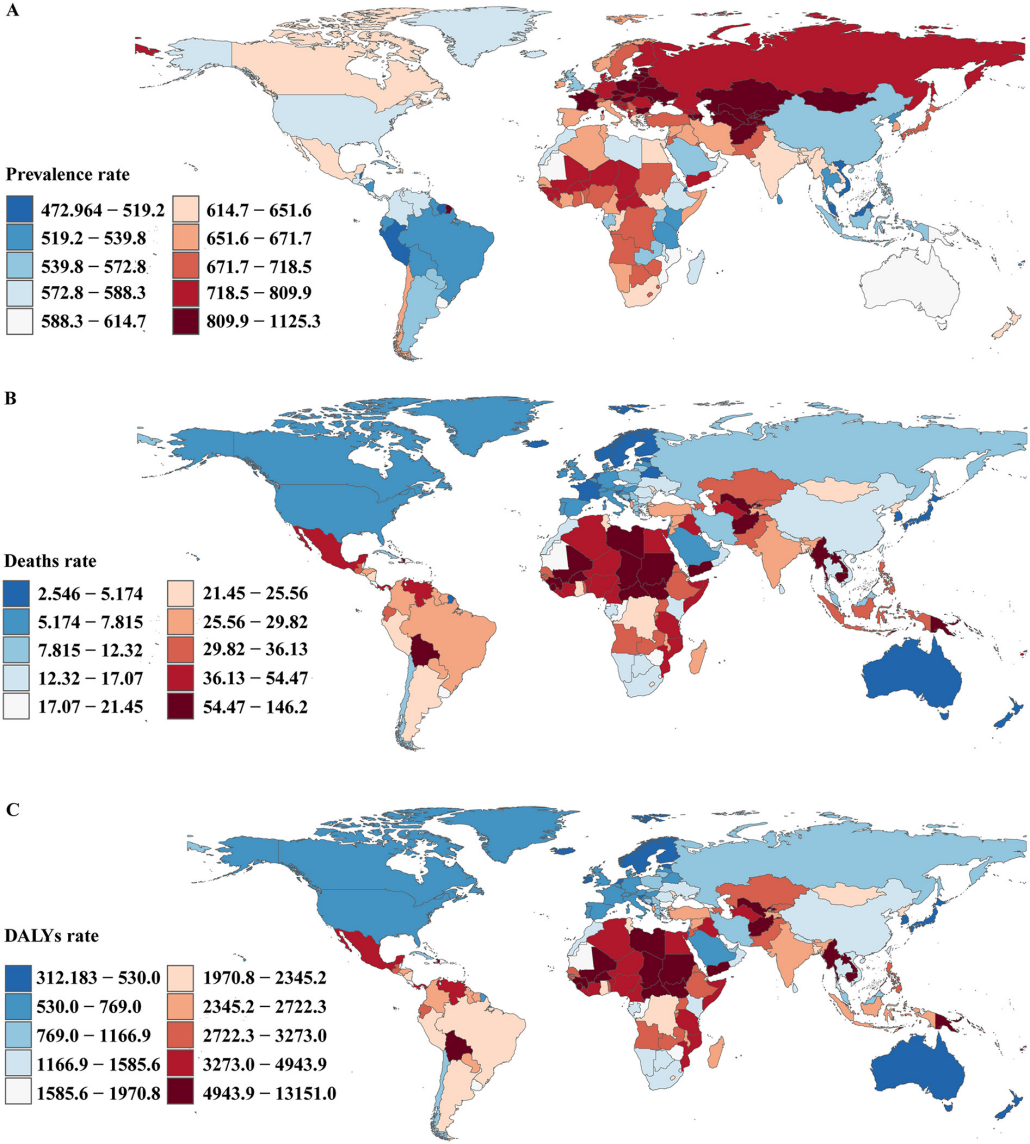
Prevalence, death, and disability-adjusted life-years rate of congenital heart disease in children in 204 countries and territories. **(A)** Prevalence rate, **(B)** deaths rate, **(C)** DALYs rate.

In 2021, among 204 countries, India had the most cases of childhood CHD (725,281; 95% UI, 623,694–841,748); Tajikistan had the highest prevalence rate of childhood CHD (1,125.3; 95% UI, 948.1–1,357.4) (Figure 3A and Supplementary Table S5). United Kingdom (EAPC, 0.8; 95% CI, 0.7–0.9) had the largest increases in childhood CHD prevalence; Canada (EAPC, −0.9; 95% CI, −1.0 to −0.8) had the largest decreases (Supplementary Figure S1A and Supplementary Table S5). In 2021, Tajikistan (SDI, 0.5) had the highest prevalence of childhood CHD, whereas Guam (SDI, 0.8) had the lowest prevalence. The global prevalence of childhood CHD in 2021 was 635.6 (95% UI, 553.6–729.3); the prevalences were above the global mean in 114 countries and below the global mean in 90 countries (Supplementary Table S5).

#### Mortality

In 2021, India had the highest number of childhood CHD-associated deaths (625; 95% UI, 466–825). Afghanistan (146.2; 95% UI, 71.5–210.4) had the highest childhood CHD-associated mortality rate; San Marino (1.4; 95% UI, 0.7–2.5)) had the lowest mortality rate (Figure 3B and Supplementary Table S3). Guatemala (EAPC, 4.0; 95% CI, 3.1–4.8) had the greatest increases in the mortality rate; Belarus (EAPC, −8.0; 95% CI, −9.2 to −6.7) and Saudi Arabia (EAPC, −7.9; 95% CI, −8.0 to −7.8) had the greatest decreases. In 2021, Afghanistan (SDI, 0.3) had the highest childhood CHD-associated mortality rate, whereas San Marino (SDI, 0.9) had the lowest mortality rate. The global childhood CHD-associated mortality rate in 2021 was 31 (95% UI, 25.1–38.8); the rates were above the global mean in 96 countries and below the global mean in 148 countries (Supplementary Figure S1B and Supplementary Table S3).

#### Disability-adjusted life years

In 2021, India had the highest number of CHD-associated childhood DALYs (3,006,729.2; 95% UI, 2,137,601.2–4,357,783.8). Afghanistan had the highest rate of childhood CHD-associated DALYs (13,151.0; 95% UI, 6,460.4–18,873.2) (Figure 3C and Supplementary Table S4). Guatemala (EAPC, 3.9; 95% CI, 3.1–4.7) had the greatest increase in DALYs rate; Saudi Arabia (EAPC, −7.7; 95% CI, −7.8 to −7.7) and Belarus (EAPC, −7.63; 95% CI, −8.8 to −6.4) had the greatest decreases (Supplementary Figure S1C and Supplementary Table S4). Afghanistan (SDI, 0.3) had the highest rate of childhood CHD-associated DALYs; San Marino (SDI, 0.9) had the lowest rate. The global rate of childhood CHD-associated DALYs in 2021 was 2,825.8 (95% UI, 2,297.8–3,522.1); the rates were above the global mean in 55 countries and below the global mean in 149 countries (Supplementary Figure S1C and Supplementary Table S4).

### Correlations between EAPC and health metrics in relation to CHD burden

**Figure 4:**
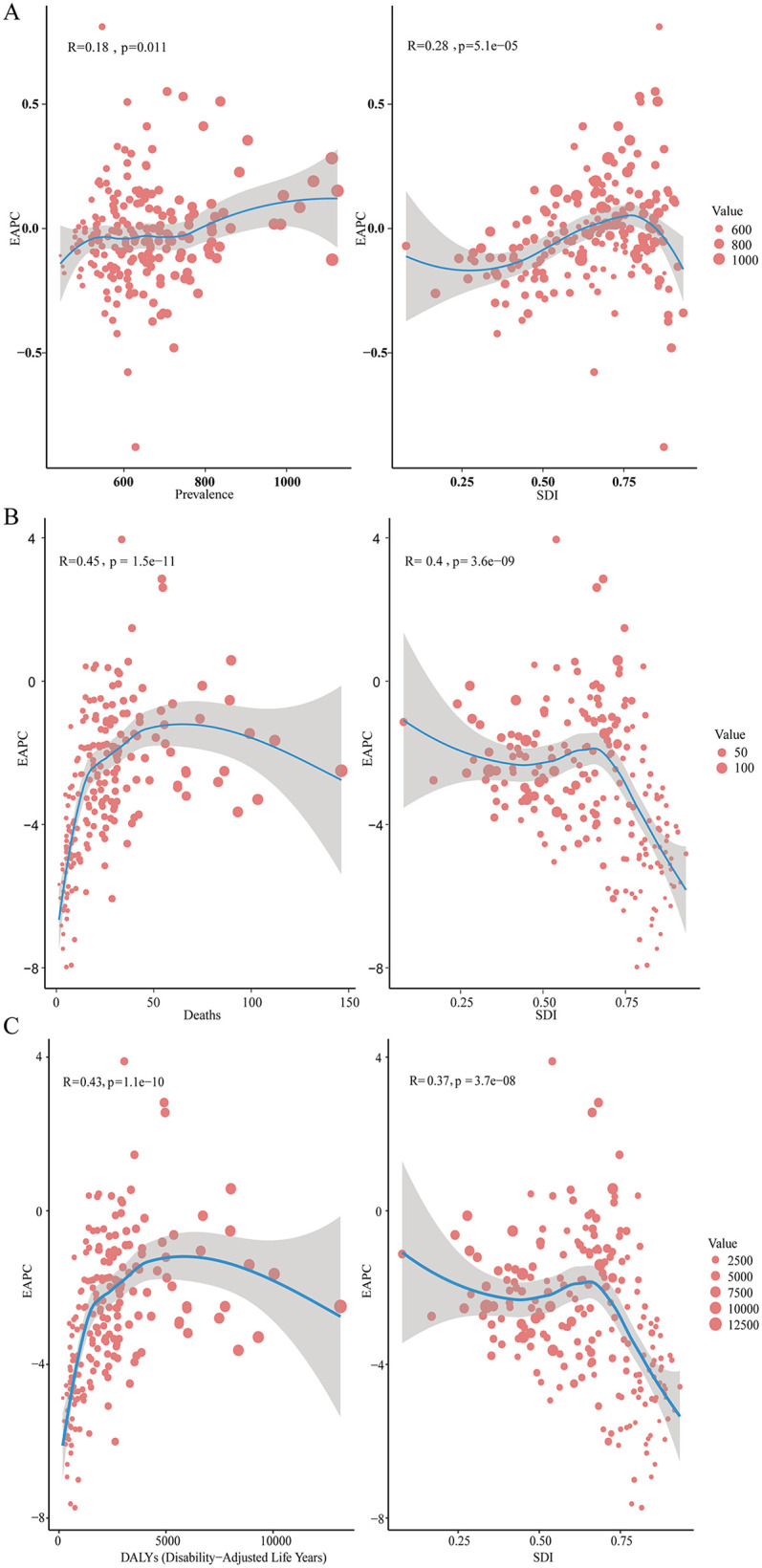
The correlation between EAPC and SDI, as well as the correlation between EAPC and the prevalence rate, death rate, and DALYs rate of childhood congenital heart disease. **(A)** EAPC and prevalence rate and SDI. **(B)** EAPC and death rate and SDI. **(C)** EAPC and DALYs rate and SDI.

In 2021, a detailed analysis of the EAPC revealed significant correlations with various health metrics, underscoring the complex interactions between healthcare quality, as measured by the SDI, and disease burden across different regions. EAPC showed a notable positive correlation with the DALYs rate (*R* = 0.43, *p* = 1.1e−10) and the death rate (*R* = 0.45, *p* = 1.5e−11), indicating that regions experiencing higher disease burden and mortality are undergoing more rapid changes in health outcomes. A positive correlation was also observed between EAPC and prevalence rates (*R* = 0.18, *p* = 0.011). Furthermore, when examining the relationship between EAPC and SDI, distinct patterns emerged across different health indicators. EAPC was positively correlated with SDI in the context of prevalence rates (*R* = 0.28, *p* = 5.1e−05), suggesting that regions with higher healthcare quality are witnessing faster increases in prevalence. Conversely, EAPC demonstrated a negative correlation with SDI in both death rates (*R* = −0.4, *p* = 3.6e−09) and DALYs (*R* = −0.37, *p* = 3.7e−08), highlighting that areas with better healthcare infrastructure are experiencing a deceleration in the growth of mortality and overall disease burden (Figure 4 and Supplementary Figure S2).

## Discussion

Since the mid-20th century, advancements in diagnostic techniques and medical interventions have profoundly impacted mortality rates and treatment outcomes for CHD, driven by the concerted efforts of healthcare systems worldwide (22). In this study, The prevalence, mortality, and disability associated with CHD in children aged 0–5 years were systematically examined across all GBD regions and countries from 1990 to 2021. The findings provide valuable insights into the evolving burden of CHD among children under five years old across regions with varying income levels over the past three decades. These results align with previous research conducted over the same period, highlighting a concerning trend: while the global prevalence of CHD in children has seen a slight increase, significant regional disparities persist. Specifically, high-SDI, upper-middle-SDI, and middle-SDI regions have experienced a notable decline in CHD prevalence, whereas lower-middle-SDI and low-SDI regions have seen a significant rise.

In 2021, the global prevalence of CHD among children under five was estimated to be approximately 6.4 per 1,000 live births, showing no significant change from 1990. However, a systematic review from 2019 indicates that the global birth prevalence of CHD has increased over the past five decades, rising from 4.5 per 1,000 live births between 1970 and 1974 to 9.4 per 1,000 live births between 2010 and 2017, with a plateau observed between 1995 and 2009. Notably, there are regional variations in CHD birth prevalence, with Africa reporting the lowest incidence at 2.1 per 1,000 live births, while Asia, Europe, North America, South America, and Oceania exhibit higher rates, ranging from 7.2 to 9.2 per 1,000 live births (23). The observed increase in CHD birth prevalence is primarily attributable to the rise in the incidence of mild defects, such as a six-fold increase in atrial septal defects (ASD) and a two- to three-fold increase in ventricular septal defects (VSD) and patent ductus arteriosus (PDA). This trend is likely influenced by ascertainment bias, driven by the increasing use of echocardiography and pulse oximetry for CHD screening and diagnosis, particularly before 2000 (23).

In the 1960s, before the advent of cardiopulmonary bypass surgery, approximately 50% of children with CHD requiring treatment died within their first year of life, and fewer than 15% survived to adulthood (24). For example, According to the National Heart, Lung, and Blood Institute (NHLBI), TGA affects roughly 5 out of every 10,000 babies. The defect is found more frequently in Caucasians. Untreated, more than 50% of infants with transposition will die in the first month of life, 90% in the first year (25). However, profound advancements in medical science have fundamentally altered the prognosis for CHD patients. More accurate diagnostic techniques, improved surgical skills, and enhanced postoperative care have significantly reduced perioperative mortality and provided treatment options for complex congenital heart defects that were once deemed inoperable. Whereas the natural course of CHD led to an 85% mortality rate during childhood, now over 85% of affected infants can expect to reach adulthood (26).

Our study estimated that in 2021, CHD accounted for 204,223 deaths (95% UI 165,238.5–255,409.2) among children under five globally. Remarkably, since 1990, the global mortality rate for children under five with CHD has decreased by 59%, with reductions exceeding 75% in high-SDI and upper-middle-SDI regions. Supporting this trend, a population-based cohort study from Quebec, Canada, demonstrated a significant increase in the median age at death for CHD patients, from 2 years in 1997–1998 to 23 years in 2004–2005 (27). Similarly, in the United States, CHD-related mortality declined by 39.3% (1.9% annually) from 1979 to 1997, with the most substantial decrease observed in patients with transposition of the great arteries, where mortality dropped by 71% (27). These data reflect a broader global trend: as advancements in CHD treatment continue, the survival and quality of life for CHD patients are significantly improving.

In 2021, the global burden of CHD in children under five years old was estimated to account for 18,598,827 DALYs (95% UI 15,123,186–23,181,408), with a corresponding DALY rate of 2,825.8 per 100,000 (95% UI 2,297.8–3,522.1). Since 1990, high-SDI countries have achieved a remarkable 75% reduction in DALY rates, while low-SDI countries have experienced a more modest decline of 48%. Over the past decade, the pace of health gains in lower-SDI countries has, on average, accelerated compared to the previous two decades, suggesting that low-income countries have the potential to significantly alter their health trajectories through strategic investments. However, progress has not been uniform. Recently, the decline in DALY rates across higher-SDI countries has been particularly striking, suggesting a near-universal trend. These findings underscore the need for regular, detailed reporting of population health outcomes by cause, to help policymakers identify successful disease control strategies and pinpoint opportunities for improvement by emulating the practices of well-performing nations.

The observed positive correlation between the annual rate of change EAPC of CHD prevalence rates and higher SDI, which generally corresponds to higher healthcare quality, could be attributed to several reasons:(1) Improved Diagnostic Capacity and Screening Programs; (2) Increased Survival Rates and Better Clinical Management; (3) Enhanced Disease Surveillance and Reporting Systems. Therefore, the increasing prevalence rates in regions with higher healthcare quality are likely due to improved detection, enhanced survival outcomes, and better disease monitoring rather than an actual rise in the underlying incidence of CHD.

CHD is a multifaceted condition, with its etiology often eluding clear definition despite well-established risk factors such as genetic predispositions and environmental exposures, including chromosomal abnormalities like Down syndrome, maternal infections, or teratogenic substances during pregnancy (28). The clinical presentation of CHD varies widely, primarily depending on the defect's severity; severe anomalies often manifest shortly after birth with symptoms like respiratory distress, feeding difficulties, or cyanosis (29). Diagnostic approaches commonly involve echocardiography, cardiac MRI, or cardiac catheterization, and in some cases, prenatal detection is feasible through fetal ultrasound (30). Treatment strategies are highly individualized, ranging from routine monitoring for minor defects to surgical correction or catheter-based interventions for more severe abnormalities. While advances in medical technology have greatly improved the prognosis for many CHD patients, enabling a large proportion to lead normal lives post-treatment, those with complex congenital heart defects may require lifelong follow-up to manage complications such as arrhythmias or heart failure (31).

In the 1990s, significant advancements were made in the diagnosis and treatment of CHD (32). During this period, echocardiography emerged as the primary diagnostic tool, offering non-invasive, detailed cardiac anatomical information that enabled early detection (33). Concurrently, surgical intervention was the predominant treatment approach, allowing for the correction of complex congenital heart defects, such as Tetralogy of Fallot and ventricular septal defects, thereby markedly improving patient survival rates (34). With the dawn of the 21st century, the focus in CHD care shifted towards minimally invasive surgery and interventional procedures, such as catheter-based closure techniques. These innovations substantially reduced surgical trauma, shortened hospital stays, and lowered complication rates (35). Notably, catheter-based technologies, including percutaneous pulmonary valve implantation and device closure of patent ductus arteriosus, became crucial in managing mild to moderate CHD. As medical technology continued to advance into the 2010s, there was a growing emphasis on comprehensive treatment strategies and long-term follow-up. Improved survival rates among patients with complex CHD led to a significant increase in the number of CHD patients reaching adulthood. Simultaneously, the application of emerging imaging modalities, such as cardiac magnetic resonance imaging and three-dimensional echocardiography, became increasingly prevalent in both the diagnosis and management of CHD, further enhancing patient care (36). In recent years, gene therapy and regenerative medicine have gained prominence as research focal points (37). Although these technologies are still in the experimental stages, they hold promise for potentially curing certain congenital heart diseases. Moreover, the steady development of personalized medicine strategies based on patient genomic data aims to tailor the most appropriate treatment for each individual, paving the way for more precise and effective care.

Our study is subject to several limitations. First, it is constrained by the variability in data quality and the presence of missing data within the GBD database. Second, the prevalence of CHD may be underreported or underestimated globally, potentially introducing bias into our findings. Third, the GBD 2021 dataset does not provide detailed information on specific types of CHD, limiting our ability to conduct a more granular analysis of the disease. Lastly, and perhaps most importantly, our study is entirely reliant on the GBD database, which inherently restricts the scope and depth of our analysis. Despite these limitations, our findings provide critical insights into the global burden of CHD and highlight the need for continued efforts to improve diagnosis, treatment, and reporting practices worldwide.

## Conclusions

This study provides a comprehensive overview of global trends and disparities in CHD among children under five years of age. Despite substantial medical advancements, CHD remains the leading cause of mortality among children under five years of age due to congenital anomalies. Between 1990 and 2021, CHD prevalence showed a modest increase, while associated mortality and DALYs experienced significant declines, reflecting improvements in healthcare delivery. However, notable regional disparities persist, particularly in low and low-middle SDI regions, which continue to bear the highest burden. In contrast, high SDI regions demonstrated the most pronounced reductions in CHD-associated outcomes, underscoring the benefits of well-established healthcare systems. To effectively reduce the CHD burden, targeted interventions are essential, with a focus on early diagnosis, enhanced access to specialized care, and equitable distribution of healthcare resources, particularly in regions with limited healthcare infrastructure.

## Data Availability

The datasets presented in this study can be found in online repositories. The names of the repository/repositories and accession number(s) can be found in the article/Supplementary Material.

## Supplementary material

Figure S1 The national burden of congenital heart disease in children in 204 countries and territories. **(A)** EAPC for prevalence rate. **(B)** EAPC for death rate. **(C)** EAPC for DALYs rate. DALYs, disability-adjusted life-years; EAPC, estimated annual percentage change.

Figure S2 The correlation between EAPC and SDI, as well as the correlation between EAPC and the prevalent cases, death cases, and DALYs cases of childhood congenital heart disease. **(A)** EAPC and prevalent cases and SDI. **(B)** EAPC and death cases and SDI. **(C)** EAPC and DALYs cases and SDI.

Figure S3 The epidemiological trends of congenital heart disease in children under five years old across five socio-demographic index regions from 1990 to 2021 include changes in prevalence rate, mortality rate, and disability-adjusted life years (DALYs) rate, as well as the absolute numbers of prevalent cases, deaths, and DALYs. **(A)** prevalence rate, **(B)** prevalent cases, **(C)** mortality, **(D)** number of deaths, **(E)** DALYs rate, F. DALYs.
