# Supplementary figures and images for: Global, regional, and national epidemiology of congenital heart disease in children from 1990 to 2021
Source: Front Cardiovasc Med. 2025 May 16;12:1522644. doi: 10.3389/fcvm.2025.1522644 (PMC12122482; doi:10.3389/fcvm.2025.1522644)

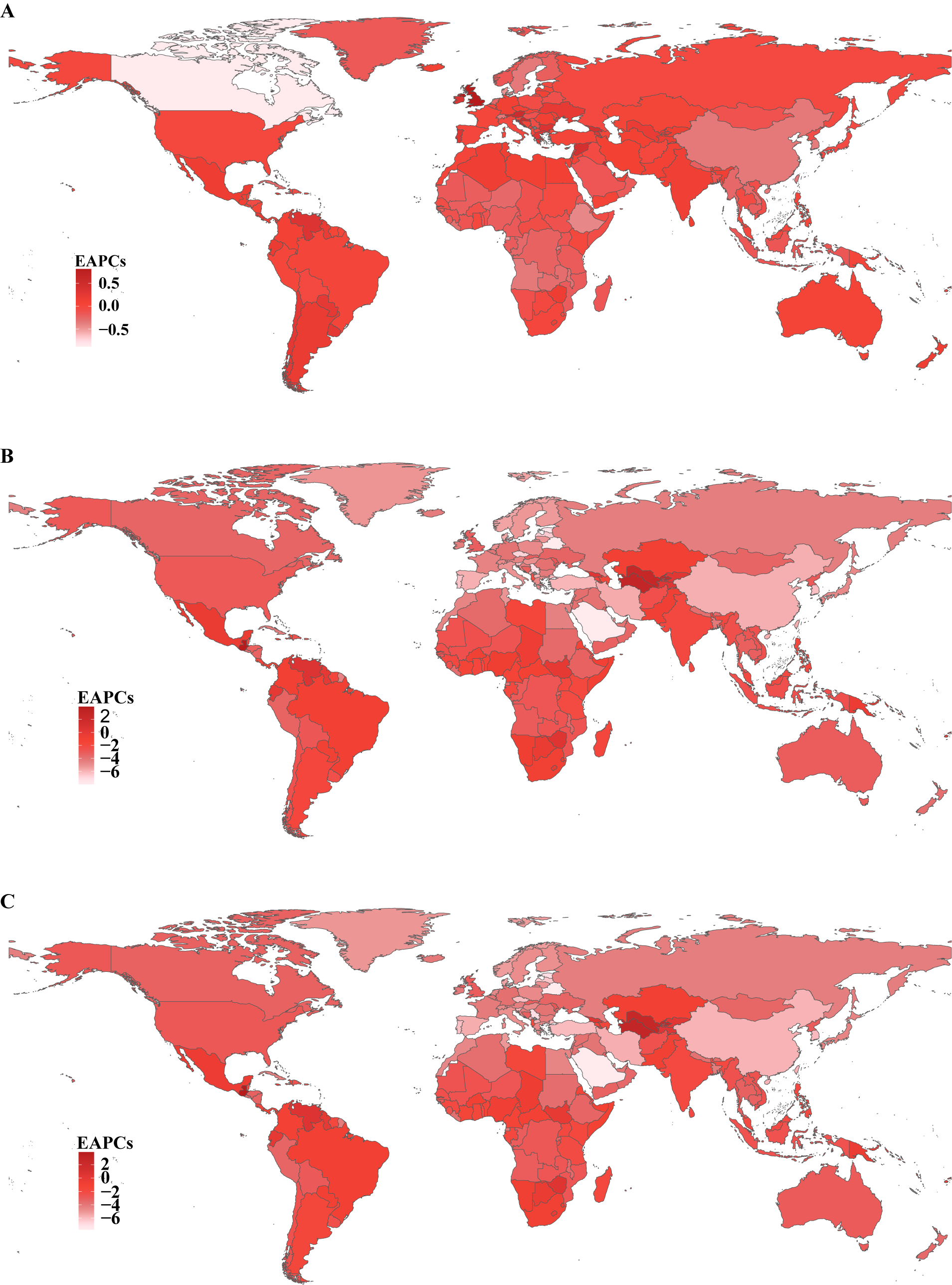

Supplement: Figure S1 — The national burden of congenital heart disease in children in 204 countries and territories. (A) EAPC for prevalence rate. (B) EAPC for death rate. (C) EAPC for DALYs rate. DALYs, disability-adjusted life-years; EAPC, estimated annual percentage change. [file Image1.tif]

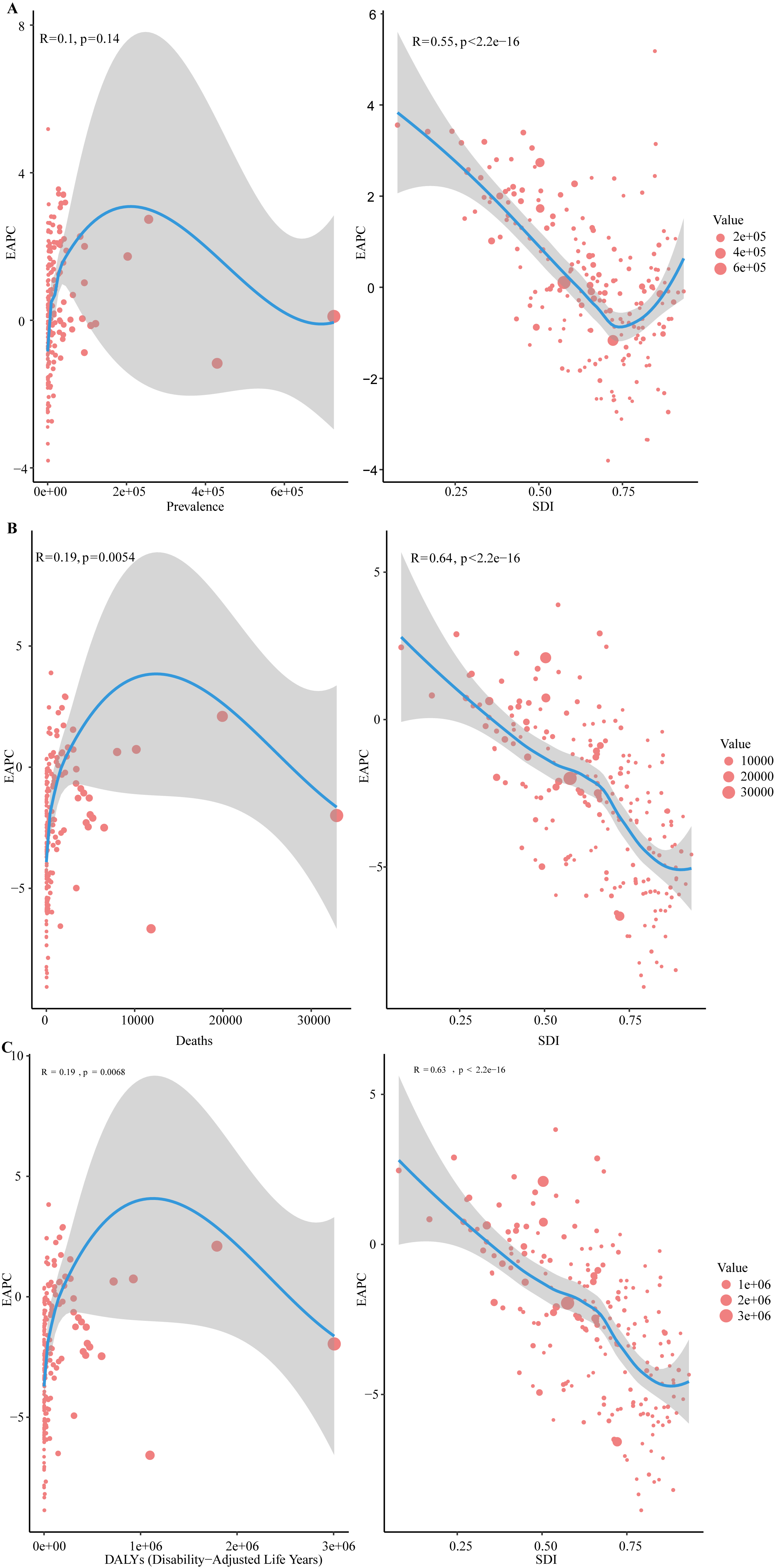

Supplement: Figure S2 — The correlation between EAPC and SDI, as well as the correlation between EAPC and the prevalent cases, death cases, and DALYs cases of childhood congenital heart disease. (A) EAPC and prevalent cases and SDI. (B) EAPC and death cases and SDI. (C) EAPC and DALYs cases and SDI. [file Image2.tif]

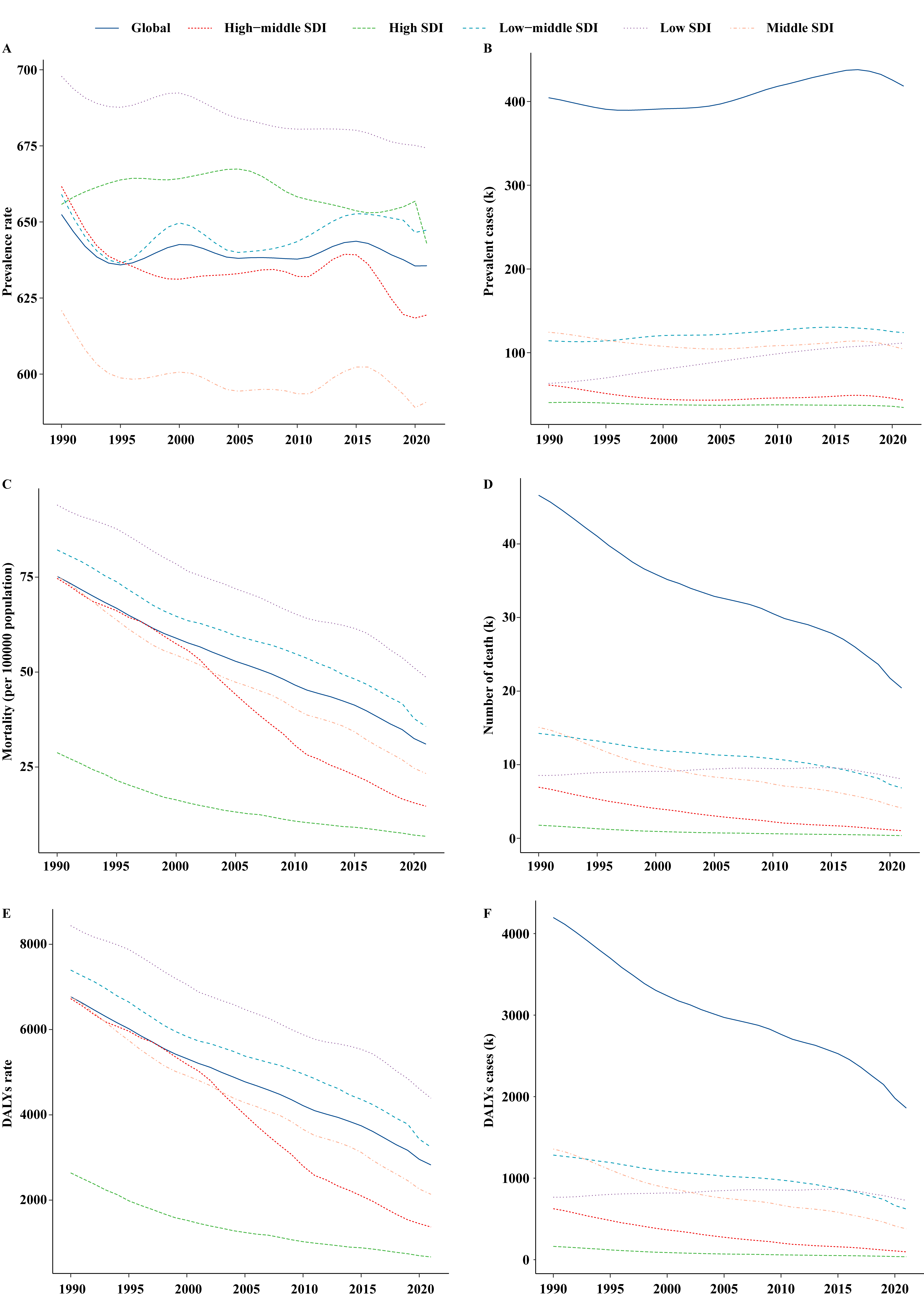

Supplement: Figure S3 — The epidemiological trends of congenital heart disease in children under five years old across five socio-demographic index regions from 1990 to 2021 include changes in prevalence rate, mortality rate, and disability-adjusted life years (DALYs) rate, as well as the absolute numbers of prevalent cases, deaths, and DALYs. (A) prevalence rate, (B) prevalent cases, (C) mortality, (D) number of deaths, (E) DALYs rate, F. DALYs. [file Image3.tif]
