# Supplementary material for: Global, regional, and national epidemiology of congenital heart disease in children from 1990 to 2021
Source: Front Cardiovasc Med. 2025 May 16;12:1522644. doi: 10.3389/fcvm.2025.1522644 (PMC12122482; doi:10.3389/fcvm.2025.1522644)
Supplement: Supplementary file 4 [file Table1.docx]

Table S1 Prevalence of Congenital Heart Disease in Children Between 1990 and 2021 at the Global and Regional Level

|  | 1990 | |  | 2021 | |  | 1990-2021 |  |
| --- | --- | --- | --- | --- | --- | --- | --- | --- |
| **Location** | prevalent cases | prevalence rate |  | prevalent cases | prevalence rate |  | Cases change | EAPC ^a^ |
| Global | 4044606.9(3517726.4-4652693.9) | 652.4(567.4-750.5) |  | 4183259.3(3643893.8-4800270.1) | 635.6(553.6-729.3) |  | 3.4(2.0-4.9) | -0.0(-0.0-0.0) |
| High SDI | 404683.6(358105.7-451399.3) | 655.8(580.3-731.5) |  | 346092.0(305663.8-387449.3) | 642.7(567.7-719.5) |  | -14.5(-16.2--12.6) | -0.1(-0.1--0.0) |
| High-middle SDI | 614700.1(531474.3-702224.5) | 661.7(572.1-755.9) |  | 433866.1(379058.7-491553.0) | 619.4(541.2-701.8) |  | -29.4(-31.7--27.1) | -0.1(-0.1--0.1) |
| Middle SDI | 1245061.5(1085364.0-1428539.6) | 620.9(541.2-712.4) |  | 1043542.8(914182.9-1183332.4) | 590.9(517.6-670.0) |  | -16.2(-18.8--13.5) | -0.1(-0.1--0.0) |
| Low-middle SDI | 1143300.3(983591.3-1339608.9) | 659.0(567.0-772.2) |  | 1240140.8(1069635.5-1442033.5) | 647.3(558.3-752.7) |  | 8.5(6.1-11.2) | 0.0(-0.0-0.1) |
| Low SDI | 633619.0(539840.6-752522.8) | 697.9(594.6-828.8) |  | 1116440.0(955993.8-1329330.8) | 674.3(577.4-802.9) |  | 76.2(73.2-79.6) | -0.1(-0.1--0.1) |
| **Regions** |  |  |  |  |  |  |  |  |
| Andean Latin America | 26967.3(23779.9-30371.0) | 510.6(450.2-575.0) |  | 31759.4(27857.2-35688.6) | 515.9(452.5-579.8) |  | 17.8(13.8-21.6) | 0.0(0.0-0.1) |
| Australasia | 9139.2(7910.6-10536.6) | 592.6(512.9-683.2) |  | 10973.0(9372.0-12718.4) | 604.2(516.1-700.3) |  | 20.1(13.7-26.3) | 0.0(-0.0-0.1) |
| Caribbean | 23072.5(20410.3-25862.0) | 558.5(494.0-626.0) |  | 21677.8(19167.4-24607.4) | 560.4(495.5-636.1) |  | -6.0(-9.5--1.8) | 0.0(-0.0-0.0) |
| Central Asia | 94552.1(80299.1-112920.5) | 992.7(843.1-1185.6) |  | 103757.4(88265.0-122194.9) | 1037.9(882.9-1222.3) |  | 9.7(6.1-13.8) | 0.1(0.1-0.2) |
| Central Europe | 70540.6(61174.8-80652.7) | 772.5(669.9-883.2) |  | 44730.4(38908.2-51056.1) | 800.8(696.6-914.1) |  | -36.6(-38.4--34.9) | 0.0(0.0-0.1) |
| Central Latin  America | 134371.0(118945.0-150253.4) | 583.8(516.7-652.8) |  | 121221.8(107400.5-136277.4) | 603.4(534.6-678.3) |  | -9.8(-12.0--7.4) | 0.1(0.1-0.2) |
| Central Sub-Saharan Africa | 77498.6(65503.6-93896.6) | 746.3(630.8-904.2) |  | 146408.5(123512.3-175866.3) | 695.0(586.3-834.8) |  | 88.9(77.1-101.3) | -0.2(-0.3--0.2) |
| East Asia | 746559.9(637798.0-872099.3) | 645.0(551.0-753.4) |  | 441554.1(382541.3-501446.6) | 551.4(477.7-626.2) |  | -40.9(-44.1--37.4) | -0.3(-0.4--0.3) |
| Eastern Europe | 136205.9(116318.8-157566.9) | 790.0(674.6-913.8) |  | 79447.6(68079.7-91417.3) | 785.1(672.8-903.4) |  | -41.7(-42.8--40.5) | -0.0(-0.1-0.0) |
| Eastern Sub-Saharan Africa | 221574.6(189971.7-259372.5) | 614.0(526.4-718.8) |  | 367418.3(316411.3-434981.4) | 575.9(496.0-681.8) |  | 65.8(62.1-69.4) | -0.2(-0.2--0.2) |
| High-income Asia Pacific | 74204.3(65641.7-83360.4) | 726.4(642.5-816.0) |  | 45268.7(40233.8-50376.5) | 701.6(623.6-780.8) |  | -39.0(-40.7--37.2) | -0.2(-0.2--0.1) |
| High-income North America | 131474.7(115470.2-148428.8) | 606.4(532.6-684.6) |  | 121294.2(106146.3-138197.8) | 591.7(517.8-674.2) |  | -7.7(-11.5--3.5) | -0.1(-0.2--0.0) |
| North Africa and Middle East | 334222.9(295232.9-376307.6) | 652.4(576.3-734.6) |  | 410491.0(363406.7-463365.8) | 671.4(594.4-757.9) |  | 22.8(20.3-25.4) | 0.1(0.1-0.1) |
| Oceania | 5860.6(5069.5-6822.2) | 583.6(504.8-679.4) |  | 11528.8(9875.9-13481.1) | 596.0(510.5-696.9) |  | 96.7(86.6-108.6) | 0.1(0.0-0.1) |
| South Asia | 1043927.2(886402.9-1231684.1) | 664.8(564.5-784.4) |  | 1039361.6(890150.3-1216481.2) | 655.4(561.3-767.0) |  | -0.4(-2.9-2.5) | 0.1(0.0-0.1) |
| Southeast Asia | 329245.5(285147.4-382394.1) | 564.8(489.2-656.0) |  | 311226.1(271243.6-357701.9) | 553.0(481.9-635.5) |  | -5.5(-7.4--3.7) | -0.1(-0.1--0.1) |
| Southern Latin America | 28251.4(24441.0-32729.9) | 548.9(474.9-635.9) |  | 25006.4(21685.2-28499.2) | 584.5(506.8-666.1) |  | -11.5(-17.2--5.9) | 0.2(0.2-0.2) |
| Southern Sub-Saharan Africa | 47760.2(41734.4-55725.3) | 639.1(558.5-745.7) |  | 51684.6(44972.1-59764.6) | 643.7(560.1-744.3) |  | 8.2(4.8-11.7) | 0.0(0.0-0.1) |
| Tropical Latin America | 90541.0(80919.7-101058.0) | 530.1(473.8-591.7) |  | 91603.8(81383.2-102557.2) | 532.3(472.9-596.0) |  | 1.2(-2.8-4.4) | 0.0(-0.1-0.1) |
| Western Europe | 156694.1(140357.4-173384.6) | 682.6(611.4-755.3) |  | 146111.5(129632.1-162330.0) | 688.3(610.6-764.7) |  | -6.8(-9.7--3.8) | 0.1(0.1-0.1) |
| Western Sub-Saharan Africa | 261943.2(220907.9-313601.1) | 732.8(618.0-877.3) |  | 560734.3(478034.3-666936.8) | 701.3(597.9-834.1) |  | 114.1(110.4-118.1) | -0.2(-0.2--0.1) |

Abbreviations: EAPC, estimated annual percentage change; SDI, Sociodemographic Index;

EAPC ^a^ is expressed as 95% CIs. Data are estimated (95% uncertainty interval).
