# Supplementary material for: Global, regional, and national epidemiology of congenital heart disease in children from 1990 to 2021
Source: Front Cardiovasc Med. 2025 May 16;12:1522644. doi: 10.3389/fcvm.2025.1522644 (PMC12122482; doi:10.3389/fcvm.2025.1522644)
Supplement: Supplementary file 5 [file Table2.docx]

Table S2 DALYs of Congenital Heart Disease in Children Between 1990 and 2021 at the Global and Regional Level

|  | 1990.00 |  |  | 2021.00 |  |  | | 1990-2021 |  |
| --- | --- | --- | --- | --- | --- | --- | --- | --- | --- |
| location | DALYs Cases | DALYs Rate |  | DALYs Cases | DALYs Rate |  | | Cases change | EAPC ^a^ |
| Global | 41970011.369(23700815.225-53981699.078) | 6770.035(3823.095-8707.598) |  | 18598827.434(15123186.365-23181408.070) | 2825.832(2297.757-3522.091) |  | | -55.685(-65.873--24.337) | -2.549(-2.662--2.436) |
| High SDI | 1628511.188(1406261.993-1781706.124) | 2638.886(2278.747-2887.128) |  | 359620.122(295423.775-427955.727) | 667.865(548.644-794.774) |  | | -77.917(-82.161--71.751) | -4.124(-4.272--3.975) |
| High-middle SDI | 6247584.800(4330727.650-7783706.580) | 6724.872(4661.576-8378.347) |  | 959878.519(786304.307-1155265.657) | 1370.377(1122.573-1649.323) |  | | -84.636(-88.691--75.469) | -5.208(-5.538--4.876) |
| Middle SDI | 13569760.487(8278968.843-17969206.290) | 6766.752(4128.424-8960.597) |  | 3773737.901(3117944.996-4632319.483) | 2136.676(1765.369-2622.802) |  | | -72.190(-80.058--46.202) | -3.332(-3.499--3.164) |
| Low-middle SDI | 12829201.031(6807080.136-17439011.280) | 7395.079(3923.775-10052.292) | | 6215113.529(4816215.640-7825278.377) | 3244.171(2513.973-4084.646) |  | | -51.555(-64.878-5.465) | -2.264(-2.410--2.118) |
| Low SDI | 7660322.209(2697490.743-11273135.377) | 8436.892(2970.951-12415.957) | | 7269819.935(4903172.875-9820650.376) | 4390.643(2961.295-5931.230) |  | | -5.098(-28.107-90.322) | -1.889(-1.976--1.801) |
| Regions |  |  |  |  |  |  | |  |  |
| Andean Latin America | 432642.378(227734.170-568583.088) | 8191.666(4311.927-10765.572) | | 185488.430(137918.145-239209.252) | 3013.227(2240.456-3885.913) |  | | -57.127(-71.094--8.952) | -2.529(-2.722--2.336) |
| Australasia | 21297.348(19543.716-23609.151) | 1380.904(1267.200-1530.800) |  | 8770.195(6503.271-10994.996) | 482.926(358.099-605.434) |  | | -58.820(-69.603--48.699) | -3.051(-3.254--2.848) |
| Caribbean | 324451.192(247881.412-411434.127) | 7853.264(5999.911-9958.665) |  | 201959.182(127342.063-323340.935) | 5221.039(3292.041-8358.994) |  | | -37.754(-57.421-4.164) | -0.948(-1.159--0.738) |
| Central Asia | 344270.015(296708.124-391898.578) | 3614.558(3115.197-4114.620) |  | 367040.532(283287.251-456508.587) | 3671.498(2833.716-4566.445) |  | | 6.614(-14.418-32.424) | 0.618(0.256-0.981) |
| Central Europe | 404266.818(345513.398-453132.211) | 4427.155(3783.742-4962.283) |  | 59412.696(47996.048-70489.104) | 1063.662(859.271-1261.962) |  | | -85.304(-89.132--81.862) | -4.610(-4.757--4.463) |
| Central Latin America | 983856.107(862254.891-1125996.627) | 4274.306(3746.017-4891.827) |  | 634184.286(478676.713-822824.220) | 3156.652(2382.614-4095.608) |  | | -35.541(-52.330--13.412) | -0.678(-0.913--0.442) |
| Central Sub-Saharan Africa | 662291.686(197175.563-1204465.073) | 6377.753(1898.766-11598.787) | | 520167.487(321864.028-835633.038) | 2469.169(1527.848-3966.644) |  | | -21.459(-43.993-85.541) | -2.734(-3.006--2.461) |
| East Asia | 9761973.355(6008710.674-13575585.660) | 8433.758(5191.165-11728.490) | | 1136570.693(864985.789-1501372.777) | 1419.407(1080.238-1874.990) |  | | -88.357(-92.459--77.604) | -5.716(-6.114--5.316) |
| Eastern Europe | 600139.007(530526.404-719390.743) | 3480.652(3076.917-4172.281) |  | 100047.082(79944.661-121573.366) | 988.713(790.051-1201.446) |  | | -83.329(-88.097--78.382) | -4.281(-5.065--3.490) |
| Eastern Sub-Saharan Africa | 2439063.714(649938.643-4711071.089) | 6758.950(1801.061-13054.965) | | 1903662.726(1126935.774-3415129.928) | 2983.967(1766.457-5353.172) |  | | -21.951(-45.886-99.290) | -2.373(-2.478--2.267) |
| High-income Asia Pacific | 271438.038(217956.584-309701.211) | 2657.010(2133.499-3031.554) |  | 32590.959(25937.323-42466.928) | 505.117(401.995-658.182) |  | | -87.993(-90.144--81.547) | -5.034(-5.189--4.878) |
| High-income North America | 421117.617(359534.848-460455.094) | 1942.265(1658.235-2123.696) |  | 140212.822(116053.633-171570.467) | 684.013(566.155-836.987) |  | | -66.705(-73.093--55.847) | -2.867(-3.059--2.675) |
| North Africa and Middle East | 8060856.995(3535110.841-11511019.785) | 15734.758(6900.521-22469.461) | | 2803823.682(2214945.650-3519698.866) | 4586.125(3622.916-5757.059) |  | | -65.217(-74.918--32.306) | -3.677(-3.861--3.491) |
| Oceania | 81303.025(26385.048-121323.861) | 8096.417(2627.508-12081.820) | | 137202.055(53657.039-210513.753) | 7092.558(2773.760-10882.351) | | 68.754(26.692-139.447) | | -0.296(-0.477--0.115) |
| South Asia | 9187372.733(5903536.577-12375348.820) | 5850.871(3759.598-7881.096) |  | 4287460.639(2973354.961-6180910.911) | 2703.423(1874.825-3897.323) |  | | -53.333(-68.484-0.824) | -2.090(-2.231--1.950) |
| Southeast Asia | 3664520.808(1697726.570-4995279.000) | 6286.745(2912.570-8569.754) |  | 1697210.391(1372581.456-2148242.440) | 3015.411(2438.647-3816.753) |  | | -53.685(-66.404-0.727) | -2.363(-2.488--2.237) |
| Southern Latin America | 175120.143(145749.665-207211.209) | 3402.414(2831.774-4025.912) |  | 73682.957(59490.292-91332.282) | 1722.183(1390.459-2134.698) |  | | -57.924(-68.145--45.505) | -1.841(-2.168--1.513) |
| Southern Sub-Saharan Africa | 154200.249(124648.573-200424.396) | 2063.459(1668.008-2682.015) |  | 123863.236(83261.795-170372.325) | 1542.676(1036.999-2121.932) |  | | -19.674(-43.207-18.514) | -0.558(-0.677--0.439) |
| Tropical Latin America | 686744.741(580382.534-801353.030) | 4020.946(3398.187-4691.987) |  | 404779.544(323176.944-496534.850) | 2352.329(1878.105-2885.554) |  | | -41.058(-55.814--22.914) | -1.070(-1.485--0.654) |
| Western Europe | 523738.823(451083.078-568742.590) | 2281.420(1964.929-2477.457) |  | 124393.211(99337.693-148107.377) | 585.953(467.929-697.658) |  | | -76.249(-81.510--69.582) | -4.328(-4.515--4.140) |
| Western Sub-Saharan Africa | 2769346.575(693091.931-4275814.739) | 7747.644(1939.024-11962.205) | | 3656304.629(2052248.470-5276682.482) | 4572.776(2566.655-6599.309) |  | | 32.028(0.199-219.710) | -1.362(-1.489--1.236) |

Abbreviations: EAPC, estimated annual percentage change; SDI, Sociodemographic Index;

EAPC ^a^ is expressed as 95% CIs. Data are estimated (95% uncertainty interval).
