# Supplementary material for: Global, regional, and national epidemiology of congenital heart disease in children from 1990 to 2021
Source: Front Cardiovasc Med. 2025 May 16;12:1522644. doi: 10.3389/fcvm.2025.1522644 (PMC12122482; doi:10.3389/fcvm.2025.1522644)
Supplement: Supplementary file 6 [file Table3.docx]

Table S3. Deaths of Childhood Congenital Heart Disease at the national level

| location | 1990 | |  | 2021 | |  | 1990-2021 | |
| --- | --- | --- | --- | --- | --- | --- | --- | --- |
|  | death case | death rate |  | death case | death rate |  | Cases change | EAPC |
| Afghanistan | 6132.67(1413.31-10075.76) | 357.76(82.45-587.79) |  | 8014.27(3916.85-11530.91) | 146.21(71.46-210.36) |  | 30.68(-12.73-220.61) | -2.50(-2.65--2.34) |
| Albania | 192.93(141.34-263.90) | 47.78(35.01-65.36) |  | 37.00(22.49-55.37) | 25.95(15.77-38.83) |  | -80.82(-89.19--68.07) | -2.01(-2.20--1.82) |
| Algeria | 6238.11(3030.81-8856.08) | 166.97(81.12-237.05) |  | 1988.55(1476.24-2635.23) | 42.27(31.38-56.01) |  | -68.12(-78.97--28.12) | -3.72(-4.00--3.44) |
| American Samoa | 1.59(1.15-2.11) | 20.88(15.13-27.72) |  | 0.51(0.33-0.76) | 13.69(9.01-20.45) |  | -68.08(-79.78--48.72) | -1.11(-1.24--0.99) |
| Andorra | 0.48(0.30-0.64) | 17.58(11.15-23.49) |  | 0.05(0.03-0.07) | 1.98(1.26-2.85) |  | -89.52(-93.98--79.88) | -6.05(-6.48--5.62) |
| Angola | 1601.53(372.55-3066.72) | 82.04(19.08-157.09) |  | 1701.90(1046.79-2761.83) | 30.21(18.58-49.03) |  | 6.27(-31.30-235.78) | -3.07(-3.35--2.79) |
| Antigua and Barbuda | 1.44(1.16-1.77) | 23.77(19.12-29.23) |  | 1.02(0.83-1.21) | 19.33(15.66-22.98) |  | -29.29(-44.08--11.58) | -0.52(-0.89--0.15) |
| Argentina | 1410.39(1141.89-1712.71) | 41.04(33.23-49.83) |  | 653.40(511.56-818.58) | 21.81(17.07-27.32) |  | -53.67(-66.18--38.36) | -1.79(-2.11--1.47) |
| Armenia | 186.77(139.95-244.57) | 48.78(36.55-63.87) |  | 46.92(34.51-62.13) | 25.20(18.54-33.37) |  | -74.88(-82.91--63.96) | -1.37(-2.36--0.37) |
| Australia | 181.97(163.53-206.26) | 14.41(12.95-16.34) |  | 69.98(49.32-89.53) | 4.65(3.28-5.96) |  | -61.54(-73.63--49.62) | -3.17(-3.40--2.93) |
| Austria | 117.55(93.39-129.95) | 26.29(20.89-29.07) |  | 23.76(18.29-30.66) | 5.50(4.23-7.10) |  | -79.79(-85.65--69.84) | -4.63(-5.11--4.15) |
| Azerbaijan | 618.59(452.99-771.20) | 68.45(50.12-85.33) |  | 257.60(158.16-424.58) | 35.73(21.93-58.88) |  | -58.36(-73.17--34.30) | -1.65(-2.10--1.20) |
| Bahamas | 8.43(6.80-10.32) | 33.06(26.67-40.47) |  | 3.62(2.52-4.91) | 17.17(11.97-23.33) |  | -57.12(-70.99--39.09) | -1.85(-2.17--1.53) |
| Bahrain | 40.34(29.72-53.06) | 65.68(48.39-86.39) |  | 10.73(8.07-14.05) | 11.45(8.61-15.00) |  | -73.40(-81.62--57.80) | -4.97(-5.45--4.48) |
| Bangladesh | 18283.17(8942.68-31268.80) | 96.58(47.24-165.17) |  | 3389.20(1528.43-6305.82) | 23.59(10.64-43.90) |  | -81.46(-89.86--43.85) | -4.27(-4.48--4.06) |
| Barbados | 6.75(5.51-8.17) | 34.46(28.15-41.72) |  | 3.47(2.40-4.85) | 25.47(17.59-35.64) |  | -48.60(-64.13--29.02) | -0.69(-0.96--0.41) |
| Belarus | 397.68(323.91-482.98) | 49.36(40.20-59.94) |  | 24.02(15.31-47.03) | 5.14(3.28-10.06) |  | -93.96(-96.32--87.55) | -7.98(-9.23--6.71) |
| Belgium | 126.60(100.07-142.61) | 21.22(16.78-23.91) |  | 37.77(28.17-47.93) | 6.38(4.76-8.10) |  | -70.17(-79.03--59.62) | -4.16(-4.68--3.63) |
| Belize | 12.28(10.01-15.13) | 41.57(33.87-51.21) |  | 7.26(5.75-9.25) | 19.05(15.08-24.26) |  | -40.84(-58.21--18.79) | -2.35(-2.66--2.03) |
| Benin | 919.08(205.75-1519.11) | 93.15(20.85-153.96) |  | 1195.07(704.37-1828.28) | 51.02(30.07-78.05) |  | 30.03(-13.64-309.27) | -1.57(-1.74--1.40) |
| Bermuda | 0.97(0.70-1.34) | 22.52(16.27-31.11) |  | 0.21(0.11-0.34) | 8.06(4.13-13.34) |  | -78.75(-86.77--68.64) | -2.93(-3.21--2.65) |
| Bhutan | 77.74(30.92-140.47) | 81.43(32.39-147.14) |  | 17.96(8.42-31.28) | 29.43(13.80-51.26) |  | -76.90(-88.04--30.01) | -3.58(-3.79--3.36) |
| Bolivia (Plurinational State of) | 1682.21(666.44-2387.71) | 166.56(65.99-236.42) |  | 743.35(542.34-986.33) | 62.25(45.42-82.59) |  | -55.81(-70.69--0.05) | -2.90(-3.04--2.76) |
| Bosnia and Herzegovina | 76.28(50.53-104.62) | 21.69(14.37-29.76) |  | 13.08(9.57-17.95) | 8.66(6.34-11.88) |  | -82.85(-87.88--73.79) | -3.26(-3.62--2.89) |
| Botswana | 38.56(26.28-53.48) | 18.10(12.34-25.11) |  | 35.29(21.41-52.02) | 14.99(9.09-22.09) |  | -8.48(-39.38-44.20) | -0.45(-0.57--0.32) |
| Brazil | 7313.08(6136.38-8589.34) | 44.42(37.28-52.18) |  | 4233.78(3366.43-5193.08) | 25.57(20.33-31.36) |  | -42.11(-57.29--24.42) | -1.10(-1.53--0.66) |
| Brunei Darussalam | 10.62(8.23-13.27) | 30.80(23.86-38.47) |  | 6.27(4.63-8.16) | 20.30(14.98-26.42) |  | -40.95(-58.51--18.43) | -0.95(-1.13--0.77) |
| Bulgaria | 296.90(258.57-341.54) | 55.31(48.17-63.62) |  | 51.42(40.72-63.33) | 16.99(13.45-20.92) |  | -82.68(-87.43--77.88) | -4.29(-4.89--3.69) |
| Burkina Faso | 2107.34(382.65-3481.96) | 112.30(20.39-185.55) |  | 3025.41(1231.02-4760.75) | 73.67(29.98-115.93) |  | 43.57(1.11-257.74) | -1.05(-1.19--0.91) |
| Burundi | 810.96(199.70-1411.56) | 75.43(18.58-131.30) |  | 675.65(373.28-1107.94) | 31.27(17.28-51.28) |  | -16.68(-45.54-121.44) | -2.20(-2.65--1.75) |
| Cabo Verde | 31.57(11.47-46.22) | 53.06(19.28-77.67) |  | 5.43(2.97-8.76) | 12.33(6.74-19.89) |  | -82.79(-92.37--42.91) | -5.04(-5.39--4.70) |
| Cambodia | 3002.36(770.64-4542.43) | 164.51(42.23-248.89) |  | 1165.87(772.20-1605.91) | 66.68(44.16-91.84) |  | -61.17(-74.52-22.07) | -3.21(-3.42--2.99) |
| Cameroon | 1423.83(403.61-2254.30) | 70.96(20.12-112.36) |  | 1938.35(1092.95-2773.26) | 39.85(22.47-57.02) |  | 36.14(-9.08-202.84) | -1.47(-1.64--1.29) |
| Canada | 427.04(347.94-473.11) | 22.18(18.07-24.58) |  | 112.38(89.03-139.95) | 5.91(4.69-7.37) |  | -73.68(-79.72--64.23) | -3.47(-3.88--3.06) |
| Central African Republic | 444.54(126.05-848.54) | 88.26(25.03-168.46) |  | 464.83(197.05-809.66) | 55.41(23.49-96.52) |  | 4.56(-25.59-88.64) | -1.22(-1.35--1.08) |
| Chad | 957.67(185.19-1535.36) | 77.05(14.90-123.53) |  | 2168.63(703.78-3350.12) | 59.65(19.36-92.15) |  | 126.45(60.41-343.29) | -0.63(-0.69--0.57) |
| Chile | 400.30(351.10-454.09) | 27.86(24.43-31.60) |  | 104.60(86.87-124.77) | 9.61(7.98-11.47) |  | -73.87(-79.29--66.91) | -2.62(-3.07--2.17) |
| China | 106810.93(64973.99-148991.73) | 95.53(58.11-133.26) |  | 11857.70(8848.44-15823.88) | 15.27(11.39-20.37) |  | -88.90(-92.91--78.20) | -5.89(-6.31--5.47) |
| Colombia | 2006.97(1668.32-2311.96) | 47.71(39.66-54.96) |  | 925.31(637.36-1328.93) | 26.88(18.51-38.60) |  | -53.89(-69.61--34.98) | -1.17(-1.62--0.72) |
| Comoros | 56.41(16.64-110.39) | 68.44(20.19-133.94) |  | 23.96(13.82-44.38) | 29.47(17.00-54.58) |  | -57.52(-73.82--1.38) | -2.55(-2.75--2.36) |
| Congo | 187.44(68.51-351.86) | 47.52(17.37-89.19) |  | 119.08(76.26-185.76) | 18.82(12.05-29.35) |  | -36.47(-59.04-62.38) | -3.08(-3.40--2.75) |
| Cook Islands | 0.36(0.21-0.53) | 16.12(9.30-23.60) |  | 0.06(0.02-0.15) | 5.35(2.15-13.47) |  | -83.33(-94.21--37.22) | -6.04(-6.93--5.15) |
| Costa Rica | 150.51(136.04-166.60) | 37.07(33.51-41.04) |  | 58.48(45.85-73.00) | 18.97(14.87-23.68) |  | -61.15(-70.30--50.55) | -1.79(-2.13--1.44) |
| Croatia | 91.50(73.84-101.37) | 30.37(24.51-33.65) |  | 14.13(10.52-18.87) | 7.76(5.77-10.36) |  | -84.55(-88.65--77.94) | -4.52(-4.88--4.16) |
| Cuba | 376.24(332.68-411.78) | 42.03(37.17-46.01) |  | 52.20(40.46-70.93) | 9.59(7.43-13.03) |  | -86.13(-89.10--80.79) | -4.41(-4.61--4.21) |
| Cyprus | 17.50(8.58-24.83) | 27.39(13.43-38.86) |  | 2.78(1.94-3.85) | 3.71(2.58-5.12) |  | -84.09(-91.18--63.64) | -6.35(-6.56--6.14) |
| Czechia | 169.32(128.68-190.10) | 26.10(19.83-29.30) |  | 14.31(8.64-19.72) | 2.55(1.54-3.51) |  | -91.55(-95.20--86.53) | -6.82(-7.20--6.44) |
| C么te d'Ivoire | 1667.42(457.11-2738.68) | 72.17(19.78-118.53) |  | 1805.00(1096.03-2718.28) | 41.45(25.17-62.43) |  | 8.25(-28.88-195.95) | -1.54(-1.73--1.35) |
| Democratic People's Republic of Korea | 1624.48(1123.61-2235.30) | 69.53(48.09-95.68) |  | 346.49(211.74-548.63) | 22.90(13.99-36.26) |  | -78.67(-88.10--61.36) | -3.03(-3.39--2.68) |
| Democratic Republic of the Congo | 5021.79(1533.43-8920.61) | 68.83(21.02-122.27) |  | 3358.81(1936.27-5530.24) | 24.77(14.28-40.78) |  | -33.12(-56.41-64.29) | -2.84(-3.15--2.53) |
| Denmark | 110.72(91.20-125.42) | 38.23(31.49-43.31) |  | 18.98(13.85-23.70) | 6.11(4.46-7.63) |  | -82.86(-87.53--76.24) | -5.73(-6.26--5.20) |
| Djibouti | 32.16(9.58-65.72) | 49.76(14.82-101.69) |  | 32.97(18.51-59.77) | 22.63(12.70-41.03) |  | 2.51(-40.59-169.91) | -2.37(-2.73--2.00) |
| Dominica | 2.31(1.77-2.98) | 26.71(20.39-34.37) |  | 1.35(0.87-1.96) | 38.79(25.12-56.24) |  | -41.67(-62.99--11.40) | 1.48(1.12-1.85) |
| Dominican Republic | 568.36(438.92-810.12) | 57.07(44.07-81.35) |  | 217.98(116.57-426.44) | 21.10(11.28-41.28) |  | -61.65(-79.28--22.84) | -2.60(-2.90--2.30) |
| Ecuador | 601.15(497.97-697.74) | 44.68(37.01-51.85) |  | 528.35(393.36-699.12) | 31.81(23.68-42.09) |  | -12.11(-36.81-19.86) | -0.08(-0.38-0.23) |
| Egypt | 15384.82(5182.88-23585.59) | 180.30(60.74-276.41) |  | 4739.92(3486.90-6320.61) | 36.36(26.75-48.48) |  | -69.19(-82.29--1.02) | -4.53(-4.88--4.18) |
| El Salvador | 756.07(399.37-1014.76) | 98.18(51.86-131.77) |  | 148.41(91.57-226.86) | 24.70(15.24-37.76) |  | -80.37(-88.68--50.23) | -3.78(-4.08--3.48) |
| Equatorial Guinea | 48.74(15.91-95.07) | 59.26(19.35-115.57) |  | 35.95(20.91-58.61) | 19.19(11.16-31.29) |  | -26.25(-62.24-187.84) | -4.05(-4.27--3.83) |
| Eritrea | 356.02(85.51-724.81) | 57.10(13.72-116.25) |  | 303.22(158.25-588.53) | 33.03(17.24-64.11) |  | -14.83(-49.24-131.89) | -1.49(-1.66--1.32) |
| Estonia | 37.25(32.56-41.66) | 30.88(27.00-34.54) |  | 2.33(1.34-3.48) | 3.36(1.94-5.04) |  | -93.76(-96.69--89.97) | -7.47(-8.30--6.63) |
| Eswatini | 33.63(19.86-47.13) | 23.13(13.66-32.41) |  | 24.10(15.99-36.59) | 17.17(11.39-26.05) |  | -28.33(-55.09-49.25) | -0.54(-0.86--0.23) |
| Ethiopia | 8207.18(1908.67-16839.78) | 85.14(19.80-174.70) |  | 4959.34(2781.88-8935.22) | 31.06(17.42-55.97) |  | -39.57(-61.85-69.74) | -3.36(-3.48--3.23) |
| Fiji | 45.61(34.25-58.56) | 48.40(36.35-62.13) |  | 40.21(28.05-56.31) | 44.16(30.80-61.85) |  | -11.84(-41.26-37.62) | -0.19(-0.51-0.13) |
| Finland | 74.87(64.22-84.87) | 23.95(20.54-27.14) |  | 11.60(7.61-15.58) | 4.78(3.14-6.42) |  | -84.51(-89.11--78.62) | -5.34(-5.58--5.09) |
| France | 857.26(702.15-918.96) | 22.04(18.05-23.63) |  | 174.14(128.92-245.15) | 4.93(3.65-6.94) |  | -79.69(-85.48--67.37) | -4.61(-4.90--4.32) |
| Gabon | 57.30(27.17-106.81) | 36.69(17.39-68.39) |  | 34.65(22.09-59.03) | 16.22(10.34-27.65) |  | -39.54(-64.53-43.72) | -1.85(-2.28--1.42) |
| Gambia | 101.06(28.13-154.80) | 54.42(15.15-83.37) |  | 92.26(60.68-132.63) | 25.80(16.97-37.09) |  | -8.71(-46.35-166.60) | -2.55(-2.77--2.32) |
| Georgia | 108.73(80.03-136.29) | 23.21(17.08-29.09) |  | 46.63(33.45-61.52) | 19.17(13.75-25.29) |  | -57.12(-68.58--41.05) | 0.35(-0.16-0.87) |
| Germany | 1023.40(859.90-1186.03) | 22.85(19.20-26.48) |  | 251.64(188.54-303.86) | 6.22(4.66-7.51) |  | -75.41(-81.62--68.86) | -4.05(-4.38--3.73) |
| Ghana | 1337.76(414.53-2119.00) | 50.79(15.74-80.45) |  | 1122.22(670.19-1624.58) | 24.19(14.44-35.01) |  | -16.11(-49.54-161.72) | -1.79(-2.00--1.58) |
| Greece | 245.06(204.42-274.80) | 44.16(36.83-49.52) |  | 39.28(30.65-48.40) | 9.29(7.25-11.45) |  | -83.97(-88.10--77.50) | -4.86(-5.33--4.39) |
| Greenland | 2.02(1.07-3.00) | 36.68(19.38-54.49) |  | 0.30(0.19-0.53) | 7.47(4.80-13.05) |  | -85.05(-92.44--69.65) | -5.19(-5.45--4.92) |
| Grenada | 4.83(3.87-6.02) | 40.24(32.27-50.12) |  | 1.97(1.52-2.53) | 28.69(22.12-36.82) |  | -59.22(-68.49--46.07) | -0.65(-0.91--0.40) |
| Guam | 3.05(2.40-4.02) | 18.85(14.85-24.89) |  | 1.93(1.35-2.71) | 15.08(10.60-21.20) |  | -36.78(-55.36--14.47) | 0.41(-0.11-0.94) |
| Guatemala | 255.74(210.30-327.79) | 16.67(13.71-21.37) |  | 522.65(373.72-691.03) | 33.52(23.97-44.32) |  | 104.37(41.13-191.54) | 3.95(3.09-4.83) |
| Guinea | 1432.02(283.14-2491.40) | 123.39(24.40-214.68) |  | 1319.56(735.48-1971.92) | 58.63(32.68-87.62) |  | -7.85(-39.15-190.08) | -1.98(-2.16--1.80) |
| Guinea-Bissau | 185.40(35.01-307.44) | 98.58(18.62-163.47) |  | 120.04(73.48-172.95) | 36.09(22.09-51.99) |  | -35.25(-62.84-140.35) | -2.99(-3.33--2.65) |
| Guyana | 46.97(38.50-56.98) | 41.64(34.13-50.51) |  | 21.51(15.05-29.50) | 28.87(20.19-39.60) |  | -54.20(-68.72--33.74) | -0.13(-0.52-0.27) |
| Haiti | 2182.58(1414.74-3112.78) | 205.77(133.38-293.46) |  | 1758.93(987.56-2923.59) | 112.04(62.91-186.23) |  | -19.41(-45.01-40.27) | -1.66(-1.90--1.41) |
| Honduras | 621.67(362.03-878.19) | 75.54(43.99-106.71) |  | 280.84(194.21-418.29) | 25.64(17.73-38.18) |  | -54.82(-72.42--3.41) | -3.26(-3.37--3.15) |
| Hungary | 236.15(194.56-271.20) | 38.14(31.42-43.80) |  | 33.78(23.55-43.13) | 7.43(5.18-9.49) |  | -85.70(-90.71--80.06) | -4.95(-5.19--4.72) |
| Iceland | 4.19(3.42-4.79) | 19.84(16.21-22.68) |  | 1.13(0.78-1.52) | 5.16(3.56-6.93) |  | -72.93(-80.73--60.24) | -4.32(-4.69--3.95) |
| India | 70761.18(42945.46-96191.74) | 60.87(36.94-82.75) |  | 32879.39(23106.30-48122.49) | 29.53(20.75-43.22) |  | -53.53(-69.55-8.64) | -1.98(-2.13--1.83) |
| Indonesia | 14290.27(7267.01-19545.89) | 64.02(32.56-87.57) |  | 6545.33(4588.13-8876.09) | 29.88(20.94-40.52) |  | -54.20(-70.09-11.30) | -2.56(-2.65--2.47) |
| Iran (Islamic Republic of) | 12580.46(6654.59-17246.88) | 143.41(75.86-196.61) |  | 616.03(406.30-885.59) | 10.01(6.60-14.39) |  | -95.10(-97.44--88.00) | -5.79(-6.87--4.69) |
| Iraq | 4243.51(2180.61-5857.56) | 135.03(69.39-186.38) |  | 1663.41(1159.89-2344.39) | 38.74(27.02-54.60) |  | -60.80(-77.07--4.78) | -3.97(-4.23--3.70) |
| Ireland | 57.44(49.40-63.15) | 19.88(17.09-21.85) |  | 15.51(11.88-19.82) | 5.20(3.98-6.64) |  | -73.00(-79.51--63.49) | -4.46(-4.75--4.16) |
| Israel | 142.77(112.81-165.90) | 27.65(21.85-32.13) |  | 47.00(34.83-59.27) | 5.12(3.79-6.45) |  | -67.08(-77.22--53.04) | -4.68(-4.96--4.40) |
| Italy | 714.17(609.23-801.25) | 26.01(22.19-29.18) |  | 129.43(93.74-161.37) | 5.96(4.32-7.44) |  | -81.88(-87.99--76.58) | -5.12(-5.30--4.94) |
| Jamaica | 97.19(72.18-121.06) | 34.83(25.87-43.39) |  | 34.83(24.92-47.97) | 20.35(14.56-28.02) |  | -64.16(-76.12--44.88) | -1.07(-1.45--0.68) |
| Japan | 1615.36(1422.34-1760.18) | 24.26(21.36-26.44) |  | 232.38(159.69-317.88) | 5.07(3.48-6.93) |  | -85.61(-90.07--79.07) | -4.77(-4.98--4.56) |
| Jordan | 606.85(417.33-765.77) | 101.11(69.53-127.58) |  | 325.53(248.23-450.69) | 29.67(22.62-41.07) |  | -46.36(-64.38--2.38) | -3.90(-4.00--3.79) |
| Kazakhstan | 776.82(578.50-991.95) | 41.26(30.72-52.68) |  | 584.15(459.00-742.64) | 29.98(23.56-38.11) |  | -24.80(-45.79-5.41) | -1.09(-2.22-0.05) |
| Kenya | 1321.69(530.28-2819.29) | 30.79(12.35-65.67) |  | 859.16(473.92-1693.83) | 14.44(7.96-28.47) |  | -35.00(-61.05-55.30) | -1.87(-2.12--1.63) |
| Kiribati | 10.67(2.54-16.92) | 90.63(21.59-143.64) |  | 6.50(2.59-9.65) | 45.46(18.13-67.50) |  | -39.13(-55.80-5.87) | -2.15(-2.27--2.02) |
| Kuwait | 110.79(90.40-136.71) | 54.19(44.22-66.87) |  | 51.70(41.19-64.69) | 19.51(15.54-24.41) |  | -53.34(-67.33--35.19) | -3.27(-3.97--2.56) |
| Kyrgyzstan | 240.21(193.94-282.62) | 37.37(30.17-43.97) |  | 250.21(197.16-305.88) | 31.52(24.83-38.53) |  | 4.16(-25.50-38.92) | 0.27(-0.38-0.93) |
| Lao People's Democratic Republic | 1376.41(286.20-2150.97) | 193.60(40.26-302.55) |  | 688.52(394.42-1008.77) | 83.00(47.55-121.61) |  | -49.98(-66.04-55.81) | -2.81(-2.97--2.66) |
| Latvia | 92.59(81.11-109.21) | 45.69(40.03-53.90) |  | 5.01(3.73-7.15) | 5.35(3.98-7.63) |  | -94.59(-96.41--91.81) | -6.63(-7.51--5.75) |
| Lebanon | 208.23(90.56-311.76) | 53.44(23.24-80.02) |  | 49.76(32.80-77.00) | 12.26(8.08-18.97) |  | -76.10(-86.68--43.49) | -4.96(-5.29--4.63) |
| Lesotho | 64.08(33.85-100.05) | 26.02(13.74-40.62) |  | 46.30(25.83-75.48) | 22.73(12.68-37.05) |  | -27.74(-54.09-19.00) | -0.48(-0.62--0.35) |
| Liberia | 637.75(104.69-1126.59) | 137.32(22.54-242.58) |  | 306.55(173.35-472.12) | 40.00(22.62-61.60) |  | -51.93(-68.51-89.51) | -3.80(-4.21--3.40) |
| Libya | 731.32(454.77-1001.77) | 114.82(71.40-157.28) |  | 236.50(151.80-345.31) | 55.90(35.88-81.62) |  | -67.66(-78.04--50.21) | -1.75(-2.28--1.20) |
| Lithuania | 116.62(103.85-134.92) | 40.34(35.92-46.67) |  | 10.34(7.19-13.55) | 7.85(5.46-10.30) |  | -91.14(-94.38--88.21) | -5.17(-5.72--4.62) |
| Luxembourg | 3.43(2.85-3.99) | 15.00(12.47-17.44) |  | 0.99(0.67-1.49) | 3.00(2.03-4.51) |  | -71.12(-80.52--54.86) | -5.11(-5.96--4.26) |
| Madagascar | 1316.54(359.15-2681.74) | 61.08(16.66-124.41) |  | 1205.51(631.91-2132.94) | 29.49(15.46-52.17) |  | -8.43(-39.30-96.07) | -1.82(-2.01--1.63) |
| Malawi | 1849.22(400.95-3603.40) | 97.39(21.12-189.78) |  | 812.00(477.62-1365.12) | 29.81(17.53-50.11) |  | -56.09(-74.09-60.93) | -3.50(-3.66--3.35) |
| Malaysia | 654.53(392.02-870.44) | 27.47(16.46-36.54) |  | 274.78(199.61-361.25) | 11.18(8.12-14.69) |  | -58.02(-71.93--15.85) | -2.15(-2.81--1.50) |
| Maldives | 29.35(8.47-44.68) | 70.25(20.28-106.93) |  | 6.23(4.44-8.89) | 19.63(13.99-28.00) |  | -78.76(-88.65--14.79) | -3.65(-3.83--3.46) |
| Mali | 2722.08(1237.26-4668.96) | 157.42(71.55-270.01) |  | 3049.86(1753.48-4457.95) | 66.59(38.28-97.33) |  | 12.04(-28.69-181.50) | -2.56(-2.71--2.42) |
| Malta | 7.59(5.78-8.78) | 26.89(20.48-31.08) |  | 2.32(1.76-2.97) | 10.56(8.02-13.50) |  | -69.39(-77.12--59.52) | -2.22(-2.70--1.73) |
| Marshall Islands | 2.63(1.32-3.61) | 35.40(17.81-48.52) |  | 1.45(0.93-2.17) | 25.52(16.37-38.24) |  | -45.00(-65.63--5.89) | -1.16(-1.59--0.73) |
| Mauritania | 182.54(47.67-274.51) | 49.21(12.85-74.00) |  | 138.39(92.87-194.51) | 21.06(14.14-29.61) |  | -24.19(-53.68-131.72) | -2.66(-3.03--2.29) |
| Mauritius | 38.77(33.09-49.50) | 36.76(31.37-46.93) |  | 17.75(13.97-23.87) | 27.61(21.72-37.13) |  | -54.20(-64.29--43.27) | -1.22(-1.52--0.93) |
| Mexico | 5419.94(4707.37-6517.73) | 45.94(39.90-55.24) |  | 3898.34(2885.61-5115.93) | 39.47(29.22-51.80) |  | -28.07(-49.62--0.30) | -0.48(-0.81--0.16) |
| Micronesia (Federated States of) | 7.92(3.19-11.62) | 50.60(20.35-74.20) |  | 1.84(1.31-2.60) | 19.45(13.82-27.46) |  | -76.75(-85.61--45.22) | -3.08(-3.18--2.99) |
| Monaco | 0.22(0.15-0.31) | 18.84(13.03-26.22) |  | 0.13(0.09-0.18) | 7.74(5.29-11.13) |  | -43.25(-65.55--6.39) | -4.21(-4.83--3.59) |
| Mongolia | 248.98(107.46-370.68) | 73.33(31.65-109.18) |  | 92.57(62.64-124.73) | 23.69(16.03-31.92) |  | -62.82(-79.94--2.37) | -3.50(-3.72--3.27) |
| Montenegro | 11.95(8.35-16.17) | 22.77(15.92-30.82) |  | 1.33(0.81-2.22) | 3.68(2.23-6.13) |  | -88.85(-93.75--77.37) | -5.87(-6.44--5.29) |
| Morocco | 2655.30(1848.07-3591.54) | 74.58(51.91-100.88) |  | 525.41(301.42-1080.69) | 16.16(9.27-33.24) |  | -80.21(-89.53--57.14) | -4.66(-4.99--4.34) |
| Mozambique | 2644.40(576.96-5098.31) | 109.77(23.95-211.64) |  | 2127.20(1124.18-3825.22) | 41.07(21.71-73.86) |  | -19.56(-49.25-121.67) | -2.75(-2.89--2.61) |
| Myanmar | 8892.47(2367.00-14303.08) | 176.36(46.94-283.67) |  | 4503.77(2616.65-6403.53) | 86.17(50.07-122.52) |  | -49.35(-66.53-31.67) | -2.51(-2.79--2.23) |
| Namibia | 47.63(29.43-67.48) | 21.04(13.00-29.81) |  | 37.76(23.86-58.19) | 13.56(8.57-20.90) |  | -20.72(-52.64-54.68) | -0.82(-1.08--0.57) |
| Nauru | 0.77(0.37-1.11) | 46.85(22.50-67.80) |  | 0.51(0.31-0.75) | 36.20(22.19-53.94) |  | -33.90(-55.71-4.06) | -0.90(-1.55--0.24) |
| Nepal | 1752.10(1197.78-2371.24) | 53.20(36.37-72.00) |  | 472.33(245.12-1053.71) | 15.20(7.89-33.92) |  | -73.04(-86.74--34.69) | -3.88(-3.97--3.78) |
| Netherlands | 208.91(172.37-233.99) | 22.31(18.41-24.99) |  | 43.87(35.81-57.33) | 5.09(4.16-6.66) |  | -79.00(-83.45--69.71) | -4.95(-5.25--4.65) |
| New Zealand | 45.85(40.47-51.94) | 16.40(14.47-18.57) |  | 15.68(11.25-21.47) | 5.02(3.60-6.87) |  | -65.80(-74.74--55.09) | -3.82(-4.23--3.41) |
| Nicaragua | 528.42(265.87-771.32) | 79.64(40.07-116.25) |  | 153.87(101.92-226.06) | 23.65(15.67-34.75) |  | -70.88(-84.31--28.70) | -3.35(-3.53--3.18) |
| Niger | 1837.56(260.17-3187.92) | 108.93(15.42-188.97) |  | 2436.60(1002.49-3804.88) | 47.81(19.67-74.66) |  | 32.60(-9.90-328.55) | -2.77(-3.01--2.53) |
| Nigeria | 12661.18(2714.71-19785.92) | 79.65(17.08-124.48) |  | 19933.73(9694.02-30922.06) | 53.70(26.12-83.31) |  | 57.44(13.62-284.03) | -0.82(-0.98--0.66) |
| Niue | 0.08(0.05-0.12) | 33.07(21.52-45.63) |  | 0.11(0.08-0.13) | 89.68(70.43-114.42) |  | 24.85(-8.21-86.38) | 0.58(-0.35-1.52) |
| North Macedonia | 124.56(88.49-157.80) | 73.12(51.95-92.63) |  | 7.95(5.61-11.64) | 7.91(5.58-11.58) |  | -93.62(-96.13--87.33) | -5.86(-6.32--5.39) |
| Northern Mariana Islands | 0.67(0.47-0.97) | 14.17(9.94-20.47) |  | 0.24(0.16-0.34) | 7.38(5.03-10.51) |  | -64.87(-73.62--49.56) | -1.27(-1.72--0.81) |
| Norway | 62.17(56.84-68.72) | 22.54(20.61-24.91) |  | 10.21(6.38-17.70) | 3.63(2.27-6.30) |  | -83.57(-89.67--71.18) | -5.62(-5.93--5.30) |
| Oman | 236.16(136.19-341.09) | 72.07(41.56-104.09) |  | 71.37(54.68-96.49) | 16.83(12.89-22.76) |  | -69.78(-81.59--35.79) | -3.52(-4.37--2.66) |
| Pakistan | 11008.42(7527.89-14742.84) | 59.63(40.78-79.86) |  | 10184.47(6087.14-14906.35) | 34.26(20.48-50.15) |  | -7.48(-37.61-60.87) | -0.90(-1.28--0.53) |
| Palau | 0.66(0.34-0.98) | 44.36(22.76-66.64) |  | 0.21(0.15-0.29) | 22.19(15.38-30.21) |  | -67.92(-78.41--44.10) | -1.84(-2.05--1.63) |
| Palestine | 417.11(259.95-568.67) | 106.95(66.66-145.82) |  | 152.38(105.90-215.68) | 24.87(17.28-35.20) |  | -63.47(-78.81--32.65) | -4.40(-4.69--4.10) |
| Panama | 179.19(150.11-215.59) | 62.75(52.57-75.50) |  | 158.57(119.22-203.98) | 42.72(32.12-54.95) |  | -11.50(-35.69-23.06) | -1.00(-1.16--0.83) |
| Papua New Guinea | 708.63(180.08-1106.23) | 109.41(27.80-170.80) |  | 1353.26(460.94-2109.63) | 88.95(30.30-138.67) |  | 90.97(40.61-198.10) | -0.53(-0.72--0.34) |
| Paraguay | 266.58(194.14-385.38) | 43.20(31.46-62.46) |  | 183.37(113.99-279.96) | 28.21(17.54-43.07) |  | -31.21(-64.31-28.59) | -1.04(-1.28--0.80) |
| Peru | 2527.95(1278.10-3431.42) | 86.40(43.68-117.27) |  | 764.79(462.37-1115.49) | 23.17(14.01-33.80) |  | -69.75(-83.90--18.81) | -3.42(-3.74--3.09) |
| Philippines | 6206.62(3579.58-8895.84) | 67.09(38.69-96.16) |  | 3584.21(2718.33-4927.82) | 31.96(24.24-43.95) |  | -42.25(-59.36-5.06) | -1.96(-2.12--1.79) |
| Poland | 1495.72(1227.63-1724.56) | 51.24(42.06-59.08) |  | 213.06(155.68-269.08) | 11.32(8.27-14.30) |  | -85.76(-90.70--81.14) | -4.83(-5.16--4.50) |
| Portugal | 219.61(184.67-253.87) | 37.97(31.93-43.89) |  | 24.39(17.48-30.28) | 5.73(4.11-7.12) |  | -88.89(-92.61--85.23) | -6.40(-6.85--5.95) |
| Puerto Rico | 83.67(71.96-94.40) | 26.21(22.54-29.57) |  | 13.88(10.57-17.21) | 13.21(10.06-16.37) |  | -83.41(-87.16--78.92) | -2.59(-3.01--2.17) |
| Qatar | 25.21(15.69-35.08) | 49.63(30.89-69.04) |  | 16.38(10.90-23.48) | 8.89(5.92-12.74) |  | -35.04(-59.24-26.46) | -5.08(-5.24--4.92) |
| Republic of Korea | 1238.23(760.19-1636.95) | 37.31(22.90-49.32) |  | 69.53(49.52-101.86) | 4.49(3.20-6.57) |  | -94.38(-96.80--88.04) | -6.27(-6.45--6.10) |
| Republic of Moldova | 247.17(175.20-326.31) | 57.37(40.66-75.73) |  | 34.09(24.63-46.22) | 22.11(15.97-29.97) |  | -86.21(-90.38--79.88) | -2.84(-3.32--2.37) |
| Romania | 1008.14(837.48-1211.56) | 56.97(47.33-68.47) |  | 150.03(124.32-179.23) | 16.00(13.26-19.11) |  | -85.12(-89.20--80.88) | -3.97(-4.48--3.45) |
| Russian Federation | 3917.53(3462.61-4859.23) | 33.70(29.79-41.80) |  | 700.75(518.48-891.93) | 9.21(6.81-11.72) |  | -82.11(-88.42--76.01) | -4.42(-5.37--3.46) |
| Rwanda | 976.34(235.47-1851.33) | 72.44(17.47-137.35) |  | 494.08(294.29-806.24) | 28.26(16.83-46.11) |  | -49.39(-71.49-51.25) | -3.36(-3.71--3.01) |
| Saint Kitts and Nevis | 1.82(1.54-2.13) | 38.83(32.97-45.41) |  | 0.70(0.52-0.92) | 22.89(17.00-30.22) |  | -61.61(-71.69--47.67) | -1.35(-1.55--1.14) |
| Saint Lucia | 5.89(4.67-7.15) | 33.33(26.43-40.52) |  | 1.75(1.22-2.43) | 19.78(13.76-27.52) |  | -70.32(-80.35--56.26) | -1.05(-1.36--0.75) |
| Saint Vincent and the Grenadines | 5.50(4.39-6.84) | 43.02(34.39-53.52) |  | 1.24(0.90-1.65) | 17.17(12.46-22.95) |  | -77.53(-84.84--67.96) | -2.75(-3.15--2.34) |
| Samoa | 10.18(5.59-14.57) | 39.93(21.94-57.14) |  | 5.51(3.67-7.99) | 18.86(12.56-27.31) |  | -45.83(-68.54-10.89) | -2.20(-2.28--2.13) |
| San Marino | 0.11(0.07-0.16) | 9.12(5.89-13.42) |  | 0.02(0.01-0.03) | 1.35(0.70-2.47) |  | -84.87(-92.52--66.95) | -5.67(-5.91--5.43) |
| Sao Tome and Principe | 13.92(4.00-21.61) | 67.96(19.55-105.52) |  | 3.88(2.27-6.72) | 15.54(9.10-26.94) |  | -72.15(-88.06-34.65) | -4.55(-4.93--4.17) |
| Saudi Arabia | 2428.37(1249.99-3535.54) | 100.44(51.70-146.24) |  | 186.72(101.43-318.23) | 7.67(4.17-13.08) |  | -92.31(-96.62--78.70) | -7.93(-8.02--7.83) |
| Senegal | 1100.15(262.41-1765.01) | 75.09(17.91-120.47) |  | 706.77(491.14-971.30) | 31.12(21.62-42.76) |  | -35.76(-61.80-129.20) | -2.43(-2.77--2.10) |
| Serbia | 499.18(313.65-648.23) | 73.33(46.08-95.23) |  | 34.40(24.81-47.91) | 9.33(6.73-12.99) |  | -93.11(-95.73--87.04) | -7.21(-7.95--6.47) |
| Seychelles | 2.89(2.32-3.60) | 35.82(28.84-44.73) |  | 2.55(1.84-3.38) | 32.35(23.37-42.90) |  | -11.74(-36.54-22.93) | 0.21(-0.02-0.44) |
| Sierra Leone | 1125.12(188.12-1924.26) | 145.40(24.31-248.68) |  | 891.55(430.51-1399.15) | 66.39(32.06-104.18) |  | -20.76(-49.73-162.95) | -2.50(-2.62--2.38) |
| Singapore | 95.38(80.24-106.34) | 46.75(39.32-52.12) |  | 10.19(6.24-15.51) | 3.56(2.18-5.43) |  | -89.32(-93.35--82.36) | -7.06(-7.66--6.45) |
| Slovakia | 133.95(109.33-157.77) | 32.74(26.72-38.56) |  | 32.24(24.40-42.24) | 11.27(8.53-14.76) |  | -75.93(-82.41--68.09) | -3.14(-3.40--2.89) |
| Slovenia | 32.82(25.14-37.30) | 26.69(20.44-30.33) |  | 3.55(2.31-4.65) | 3.63(2.36-4.75) |  | -89.18(-93.29--82.92) | -6.40(-6.57--6.24) |
| Solomon Islands | 35.98(16.72-52.23) | 60.32(28.03-87.56) |  | 30.13(20.69-42.64) | 31.60(21.71-44.72) |  | -16.26(-44.06-67.38) | -2.03(-2.16--1.91) |
| Somalia | 1101.67(244.18-2380.62) | 71.30(15.80-154.08) |  | 1766.06(601.93-3800.92) | 42.77(14.58-92.05) |  | 60.31(10.52-182.78) | -1.14(-1.44--0.84) |
| South Africa | 1152.84(929.02-1539.42) | 23.63(19.04-31.56) |  | 751.45(491.35-1118.23) | 15.15(9.90-22.54) |  | -34.82(-58.60-4.47) | -1.00(-1.14--0.86) |
| South Sudan | 879.09(187.94-1801.48) | 86.37(18.47-177.00) |  | 1169.13(396.07-2112.38) | 74.83(25.35-135.21) |  | 32.99(-11.47-147.01) | -0.13(-0.58-0.33) |
| Spain | 601.67(521.74-681.30) | 28.89(25.05-32.71) |  | 103.93(74.72-126.84) | 5.64(4.06-6.89) |  | -82.73(-88.32--77.94) | -5.93(-6.25--5.61) |
| Sri Lanka | 640.87(408.29-933.64) | 36.25(23.10-52.81) |  | 266.90(187.32-390.17) | 17.05(11.96-24.92) |  | -58.35(-73.93--15.40) | -2.06(-2.47--1.66) |
| Sudan | 11127.59(3110.98-18699.52) | 319.68(89.37-537.21) |  | 5251.66(3410.69-7424.71) | 93.13(60.48-131.67) |  | -52.81(-70.02-24.77) | -3.65(-3.87--3.42) |
| Suriname | 22.09(16.25-28.51) | 50.42(37.09-65.09) |  | 12.80(8.17-18.69) | 28.74(18.34-41.96) |  | -42.05(-63.29--11.27) | -1.82(-1.99--1.65) |
| Sweden | 125.61(105.37-140.71) | 22.31(18.71-24.99) |  | 18.47(12.35-29.07) | 3.17(2.12-4.98) |  | -85.29(-90.20--73.81) | -5.26(-5.75--4.77) |
| Switzerland | 144.37(117.09-162.05) | 36.24(29.40-40.68) |  | 31.50(24.86-38.29) | 7.13(5.62-8.66) |  | -78.18(-83.39--71.03) | -4.82(-5.31--4.32) |
| Syrian Arab Republic | 2805.88(1573.01-3793.65) | 130.09(72.93-175.88) |  | 280.54(199.81-389.30) | 27.91(19.88-38.73) |  | -90.00(-93.64--76.84) | -3.83(-4.57--3.09) |
| Taiwan (Province of China) | 421.38(389.58-451.87) | 26.23(24.25-28.13) |  | 75.48(60.11-90.42) | 8.46(6.74-10.13) |  | -82.09(-85.84--77.79) | -3.97(-4.39--3.55) |
| Tajikistan | 287.17(215.67-427.19) | 30.37(22.81-45.18) |  | 354.52(202.33-748.40) | 26.49(15.12-55.91) |  | 23.45(-37.42-186.97) | 0.38(0.12-0.64) |
| Thailand | 2250.00(1525.52-3291.58) | 43.27(29.34-63.30) |  | 440.31(290.42-573.55) | 15.57(10.27-20.29) |  | -80.43(-87.05--69.34) | -3.27(-3.47--3.06) |
| Timor-Leste | 198.94(50.88-320.93) | 142.05(36.33-229.15) |  | 114.98(72.96-165.86) | 62.20(39.47-89.73) |  | -42.20(-62.73-71.81) | -2.97(-3.17--2.76) |
| Togo | 444.41(116.05-678.56) | 64.75(16.91-98.87) |  | 361.36(229.87-521.87) | 30.82(19.61-44.51) |  | -18.69(-46.97-116.27) | -2.10(-2.24--1.96) |
| Tokelau | 0.07(0.04-0.10) | 36.03(20.16-49.82) |  | 0.10(0.07-0.15) | 99.17(68.30-149.07) |  | 40.12(-16.10-246.65) | -1.46(-3.31-0.42) |
| Tonga | 3.66(2.26-5.18) | 23.99(14.80-33.91) |  | 1.84(1.22-2.94) | 12.75(8.47-20.43) |  | -49.89(-69.98--1.47) | -1.84(-2.10--1.58) |
| Trinidad and Tobago | 55.79(46.58-66.57) | 41.85(34.94-49.93) |  | 27.71(20.43-36.92) | 34.42(25.38-45.86) |  | -50.33(-65.57--26.93) | -0.54(-1.00--0.08) |
| Tunisia | 1537.48(700.08-2189.73) | 144.07(65.60-205.19) |  | 221.80(163.65-292.45) | 24.86(18.35-32.78) |  | -85.57(-91.21--67.78) | -5.15(-5.31--5.00) |
| Turkey | 14281.56(6314.00-22059.41) | 205.34(90.78-317.17) |  | 1585.43(1175.53-2061.90) | 28.56(21.17-37.14) |  | -88.90(-93.86--72.17) | -6.07(-6.24--5.89) |
| Turkmenistan | 250.47(188.76-321.46) | 42.77(32.23-54.90) |  | 292.12(202.59-388.38) | 54.12(37.54-71.96) |  | 16.63(-13.17-57.68) | 2.85(1.93-3.78) |
| Tuvalu | 1.50(0.39-2.38) | 99.75(26.17-158.08) |  | 0.33(0.21-0.48) | 25.40(16.20-37.41) |  | -78.34(-87.73--31.97) | -4.10(-4.29--3.90) |
| Uganda | 2240.45(588.19-4380.69) | 62.38(16.38-121.98) |  | 2316.41(1348.52-3846.68) | 31.65(18.43-52.57) |  | 3.39(-31.63-176.30) | -1.87(-2.08--1.67) |
| Ukraine | 1759.08(1501.43-2083.99) | 46.67(39.84-55.29) |  | 264.04(207.36-330.34) | 16.58(13.02-20.75) |  | -84.99(-88.78--80.44) | -3.45(-3.91--2.99) |
| United Arab Emirates | 155.67(102.47-214.73) | 68.29(44.95-94.20) |  | 46.49(26.22-66.65) | 10.74(6.06-15.40) |  | -70.14(-81.68--40.51) | -4.11(-4.70--3.51) |
| United Kingdom | 804.82(759.67-874.12) | 20.95(19.78-22.75) |  | 238.40(194.60-300.81) | 6.52(5.32-8.23) |  | -70.38(-76.19--63.26) | -3.22(-3.56--2.88) |
| United Republic of Tanzania | 4140.48(982.89-7862.65) | 86.12(20.44-163.53) |  | 3390.78(1829.72-5967.61) | 38.33(20.68-67.46) |  | -18.11(-49.40-135.01) | -2.11(-2.31--1.91) |
| United States of America | 4130.55(3564.78-4530.66) | 20.91(18.05-22.94) |  | 1322.34(1084.69-1659.78) | 7.11(5.83-8.93) |  | -67.99(-74.55--56.80) | -2.98(-3.17--2.79) |
| United States Virgin Islands | 3.64(2.74-4.53) | 33.06(24.87-41.19) |  | 0.35(0.21-0.61) | 8.98(5.30-15.52) |  | -90.33(-94.58--81.33) | -3.90(-4.13--3.67) |
| Uruguay | 114.45(93.37-135.57) | 41.95(34.22-49.69) |  | 37.10(28.03-47.38) | 19.12(14.45-24.42) |  | -67.58(-77.54--57.26) | -2.45(-2.74--2.17) |
| Uzbekistan | 1056.19(868.26-1250.15) | 31.31(25.74-37.06) |  | 2094.77(1479.48-2763.61) | 54.62(38.58-72.06) |  | 98.33(42.13-168.71) | 2.61(1.89-3.34) |
| Vanuatu | 12.06(4.71-17.35) | 44.60(17.40-64.15) |  | 10.90(6.65-15.34) | 25.86(15.77-36.38) |  | -9.60(-38.87-65.31) | -1.76(-2.17--1.34) |
| Venezuela (Bolivarian Republic of) | 940.43(837.98-1038.46) | 37.16(33.11-41.03) |  | 805.44(555.97-1098.23) | 36.86(25.45-50.26) |  | -14.35(-41.12-18.54) | 0.54(0.37-0.72) |
| Viet Nam | 3033.76(1230.23-4208.54) | 32.24(13.07-44.73) |  | 1025.79(660.56-1539.07) | 12.60(8.11-18.90) |  | -66.19(-80.15--10.79) | -2.60(-2.93--2.26) |
| Yemen | 7762.36(2128.33-12591.35) | 279.57(76.65-453.48) |  | 4853.89(3041.45-6709.90) | 103.33(64.75-142.85) |  | -37.47(-58.80-52.94) | -3.30(-3.44--3.16) |
| Zambia | 1201.15(307.74-2313.74) | 79.59(20.39-153.31) |  | 881.92(528.73-1559.78) | 30.15(18.08-53.33) |  | -26.58(-63.81-215.62) | -2.76(-3.01--2.50) |
| Zimbabwe | 349.71(244.40-462.93) | 19.83(13.86-26.25) |  | 446.65(297.36-639.76) | 20.21(13.46-28.95) |  | 27.72(-7.49-94.90) | 0.46(0.24-0.68) |
