# Supplementary material for: Global, regional, and national epidemiology of congenital heart disease in children from 1990 to 2021
Source: Front Cardiovasc Med. 2025 May 16;12:1522644. doi: 10.3389/fcvm.2025.1522644 (PMC12122482; doi:10.3389/fcvm.2025.1522644)
Supplement: Supplementary file 7 [file Table4.docx]

Table S4. DALYs of Childhood Congenital Heart Disease at the national level

| location | 1990 | |  | 2021 | |  | 1990-2021 | |
| --- | --- | --- | --- | --- | --- | --- | --- | --- |
|  | DALYs case | DALYs rate |  | DALYs case | DALYs rate |  | Cases change | EAPC |
| Afghanistan | 549829.84(127822.61-901639.15) | 32075.53(7456.81-52599.10) |  | 720863.69(354121.30-1034524.28) | 13150.98(6460.37-18873.21) |  | 31.11(-12.43-220.06) | -2.49(-2.64--2.34) |
| Albania | 17505.66(12890.19-23833.99) | 4335.54(3192.45-5902.85) |  | 3397.12(2099.65-5035.44) | 2382.22(1472.37-3531.09) |  | -80.59(-88.95--68.03) | -1.98(-2.16--1.79) |
| Algeria | 560863.34(273731.72-794537.73) | 15012.48(7326.91-21267.19) |  | 181192.34(134843.99-238381.78) | 3851.33(2866.17-5066.91) |  | -67.69(-78.62--27.69) | -3.69(-3.96--3.41) |
| American Samoa | 145.54(105.98-191.94) | 1909.50(1390.48-2518.27) |  | 46.99(31.60-69.66) | 1266.76(851.96-1878.01) |  | -67.72(-79.27--48.78) | -1.08(-1.20--0.96) |
| Andorra | 43.96(28.38-58.17) | 1624.03(1048.56-2148.84) |  | 5.85(4.13-8.10) | 231.81(163.64-320.73) |  | -86.69(-91.52--76.63) | -5.47(-5.86--5.09) |
| Angola | 143760.43(34353.97-274772.64) | 7364.15(1759.79-14075.28) |  | 154489.12(95535.19-249733.71) | 2742.48(1695.93-4433.25) |  | 7.46(-30.62-234.46) | -3.04(-3.32--2.76) |
| Antigua and Barbuda | 132.74(107.00-162.42) | 2190.04(1765.48-2679.81) |  | 94.35(76.89-111.34) | 1789.54(1458.34-2111.95) |  | -28.92(-43.54--11.63) | -0.51(-0.87--0.15) |
| Argentina | 128003.97(103777.27-155135.11) | 3724.50(3019.58-4513.93) |  | 60171.05(47496.05-75419.98) | 2008.20(1585.18-2517.13) |  | -52.99(-65.33--37.81) | -1.75(-2.06--1.44) |
| Armenia | 17035.50(12836.92-22186.11) | 4448.76(3352.31-5793.82) |  | 4361.39(3276.64-5704.24) | 2342.20(1759.66-3063.36) |  | -74.40(-82.45--63.68) | -1.34(-2.30--0.36) |
| Australia | 17027.40(15270.19-19048.25) | 1348.57(1209.40-1508.62) |  | 7178.68(5263.03-9050.33) | 477.48(350.06-601.97) |  | -57.84(-69.64--46.48) | -2.93(-3.15--2.72) |
| Austria | 10786.30(8605.59-11925.26) | 2412.89(1925.06-2667.67) |  | 2420.78(1908.78-3027.97) | 560.21(441.73-700.73) |  | -77.56(-83.66--67.30) | -4.33(-4.79--3.86) |
| Azerbaijan | 55961.03(41173.25-69617.09) | 6192.05(4555.79-7703.08) |  | 23578.86(14674.87-38505.10) | 3270.09(2035.22-5340.17) |  | -57.87(-72.45--34.13) | -1.62(-2.06--1.18) |
| Bahamas | 769.24(621.64-935.24) | 3016.33(2437.58-3667.26) |  | 335.71(239.66-451.56) | 1594.40(1138.24-2144.64) |  | -56.36(-69.90--38.52) | -1.80(-2.11--1.49) |
| Bahrain | 3648.32(2689.32-4784.62) | 5940.20(4378.75-7790.32) |  | 1015.41(779.33-1311.85) | 1083.80(831.82-1400.20) |  | -72.17(-80.66--56.71) | -4.85(-5.33--4.37) |
| Bangladesh | 1641977.72(805041.97-2801147.32) | 8673.52(4252.52-14796.67) |  | 310312.50(143857.06-571495.98) | 2160.34(1001.51-3978.65) |  | -81.10(-89.65--43.57) | -4.22(-4.43--4.01) |
| Barbados | 617.23(507.34-743.91) | 3151.91(2590.78-3798.85) |  | 320.18(225.38-443.95) | 2351.28(1655.14-3260.22) |  | -48.13(-63.41--28.92) | -0.66(-0.93--0.39) |
| Belarus | 36250.69(29550.97-43856.28) | 4499.03(3667.54-5442.95) |  | 2497.23(1731.73-4584.23) | 534.33(370.53-980.88) |  | -93.11(-95.59--86.83) | -7.63(-8.82--6.42) |
| Belgium | 11685.84(9319.06-13088.43) | 1959.06(1562.28-2194.20) |  | 3746.93(2849.98-4625.99) | 633.00(481.47-781.50) |  | -67.94(-76.74--57.23) | -3.90(-4.41--3.39) |
| Belize | 1112.37(908.08-1366.41) | 3765.38(3073.85-4625.31) |  | 669.30(529.88-847.91) | 1755.22(1389.59-2223.61) |  | -39.83(-57.22--18.11) | -2.30(-2.61--1.98) |
| Benin | 82373.31(18854.48-135679.99) | 8348.25(1910.84-13750.69) |  | 107575.91(63843.99-163347.46) | 4592.62(2725.62-6973.62) |  | 30.60(-13.11-303.18) | -1.56(-1.73--1.40) |
| Bermuda | 89.58(65.61-122.33) | 2085.12(1527.34-2847.65) |  | 20.10(11.24-32.31) | 787.87(440.69-1266.30) |  | -77.56(-85.09--67.86) | -2.77(-3.04--2.50) |
| Bhutan | 6986.20(2819.46-12575.16) | 7317.91(2953.33-13172.23) |  | 1634.66(779.97-2824.06) | 2678.43(1278.00-4627.30) |  | -76.60(-87.82--30.11) | -3.53(-3.75--3.32) |
| Bolivia (Plurinational State of) | 150840.79(60087.84-213904.72) | 14935.49(5949.59-21179.76) |  | 67099.90(49039.01-88816.72) | 5618.89(4106.48-7437.43) |  | -55.52(-70.35-0.10) | -2.88(-3.02--2.75) |
| Bosnia and Herzegovina | 7093.40(4817.71-9620.04) | 2017.47(1370.23-2736.08) |  | 1281.08(971.11-1722.74) | 848.17(642.95-1140.59) |  | -81.94(-87.09--73.02) | -3.09(-3.44--2.74) |
| Botswana | 3553.63(2458.59-4899.02) | 1668.20(1154.15-2299.77) |  | 3267.47(2039.36-4766.60) | 1387.47(865.98-2024.05) |  | -8.05(-37.97-42.48) | -0.44(-0.56--0.33) |
| Brazil | 662548.88(557437.20-778886.28) | 4024.68(3386.17-4731.37) |  | 387983.85(311156.11-474033.84) | 2343.25(1879.24-2862.95) |  | -41.44(-56.46--23.92) | -1.07(-1.50--0.65) |
| Brunei Darussalam | 968.84(756.98-1202.97) | 2809.35(2195.01-3488.28) |  | 578.14(429.79-746.61) | 1871.29(1391.10-2416.58) |  | -40.33(-57.54--18.02) | -0.93(-1.10--0.75) |
| Bulgaria | 26986.28(23566.39-31031.26) | 5027.11(4390.04-5780.63) |  | 4844.60(3869.46-5909.24) | 1600.56(1278.39-1952.29) |  | -82.05(-86.80--77.29) | -4.17(-4.75--3.59) |
| Burkina Faso | 188907.29(34942.12-311389.10) | 10066.87(1862.07-16593.92) |  | 271857.02(112475.69-425943.04) | 6620.22(2738.99-10372.50) |  | 43.91(1.51-253.15) | -1.04(-1.18--0.91) |
| Burundi | 72813.98(18360.89-126730.26) | 6772.77(1707.83-11787.77) |  | 61287.89(34175.98-99893.38) | 2836.89(1581.94-4623.87) |  | -15.83(-44.84-120.44) | -2.18(-2.62--1.73) |
| Cabo Verde | 2852.38(1060.98-4160.00) | 4793.40(1782.97-6990.86) |  | 508.67(287.00-805.27) | 1154.51(651.40-1827.70) |  | -82.17(-91.76--42.48) | -4.94(-5.27--4.60) |
| Cambodia | 269418.96(70036.67-407210.13) | 14762.34(3837.54-22312.37) |  | 105272.64(70037.20-144922.55) | 6020.58(4005.45-8288.18) |  | -60.93(-74.30-21.65) | -3.18(-3.40--2.97) |
| Cameroon | 127850.22(37130.07-201300.85) | 6372.11(1850.58-10032.93) |  | 174959.35(99992.61-249590.18) | 3597.24(2055.89-5131.68) |  | 36.85(-8.47-200.83) | -1.46(-1.63--1.28) |
| Canada | 39721.76(32421.48-43827.90) | 2063.41(1684.19-2276.71) |  | 11436.15(9210.14-13894.15) | 601.87(484.72-731.24) |  | -71.21(-77.32--62.17) | -3.25(-3.64--2.85) |
| Central African Republic | 39980.49(11517.38-76125.32) | 7937.44(2286.58-15113.37) |  | 41917.59(18174.08-72708.24) | 4997.08(2166.57-8667.69) |  | 4.85(-25.14-87.80) | -1.21(-1.34--1.07) |
| Chad | 85974.86(17089.91-137218.77) | 6917.18(1374.98-11040.05) |  | 194932.82(64839.57-300435.50) | 5361.90(1783.50-8263.90) |  | 126.73(61.43-337.70) | -0.63(-0.69--0.57) |
| Chile | 36703.65(32210.52-41562.89) | 2554.10(2241.44-2892.24) |  | 10062.51(8460.64-11865.34) | 924.90(777.66-1090.60) |  | -72.58(-78.16--65.83) | -2.51(-2.93--2.08) |
| China | 9577321.70(5848319.75-13339994.41) | 8566.02(5230.77-11931.37) |  | 1097777.75(831139.72-1452397.77) | 1413.42(1070.11-1870.00) |  | -88.54(-92.60--77.75) | -5.80(-6.21--5.39) |
| Colombia | 181949.11(151558.08-209281.65) | 4324.98(3602.58-4974.68) |  | 84896.53(58849.77-120928.28) | 2465.94(1709.37-3512.53) |  | -53.34(-69.01--34.66) | -1.15(-1.59--0.70) |
| Comoros | 5075.59(1516.88-9912.79) | 6158.39(1840.48-12027.54) |  | 2172.54(1263.71-3991.01) | 2672.02(1554.24-4908.56) |  | -57.20(-73.53--1.42) | -2.53(-2.72--2.34) |
| Congo | 16911.79(6275.70-31553.83) | 4286.98(1590.83-7998.60) |  | 10921.03(7131.52-16866.76) | 1725.71(1126.90-2665.24) |  | -35.42(-57.83-62.37) | -3.03(-3.35--2.71) |
| Cook Islands | 33.37(19.60-48.29) | 1489.82(874.94-2155.86) |  | 5.89(2.63-14.04) | 523.44(234.09-1248.97) |  | -82.37(-93.12--38.74) | -5.60(-6.39--4.81) |
| Costa Rica | 13735.67(12441.71-15181.75) | 3383.42(3064.69-3739.63) |  | 5444.48(4289.52-6763.08) | 1765.96(1391.34-2193.66) |  | -60.36(-69.16--49.97) | -1.74(-2.07--1.40) |
| Croatia | 8409.00(6810.95-9264.22) | 2791.13(2260.70-3074.99) |  | 1398.70(1069.18-1820.60) | 767.47(586.66-998.97) |  | -83.37(-87.48--76.68) | -4.28(-4.63--3.93) |
| Cuba | 34304.43(30291.51-37656.32) | 3832.63(3384.29-4207.11) |  | 5018.39(3945.62-6658.11) | 921.71(724.68-1222.87) |  | -85.37(-88.41--80.18) | -4.27(-4.46--4.07) |
| Cyprus | 1604.88(805.08-2258.09) | 2511.55(1259.91-3533.78) |  | 291.92(210.43-391.97) | 389.01(280.42-522.33) |  | -81.81(-89.28--60.02) | -5.94(-6.17--5.71) |
| Czechia | 15721.16(12003.02-17585.13) | 2423.06(1849.99-2710.35) |  | 1754.67(1206.17-2226.33) | 312.18(214.60-396.10) |  | -88.84(-92.75--83.56) | -6.08(-6.45--5.70) |
| C么te d'Ivoire | 149996.41(41932.37-245158.45) | 6492.08(1814.90-10610.84) |  | 163157.99(100040.09-244676.58) | 3746.98(2297.45-5619.08) |  | 8.77(-28.46-192.45) | -1.53(-1.71--1.34) |
| Democratic People's Republic of Korea | 146214.93(101524.78-200727.41) | 6258.35(4345.50-8591.61) |  | 31629.05(19749.30-49832.24) | 2090.32(1305.20-3293.34) |  | -78.37(-87.81--61.09) | -3.00(-3.34--2.65) |
| Democratic Republic of the Congo | 452059.43(140744.74-800113.98) | 6196.33(1929.17-10967.07) |  | 306356.38(178898.04-501709.46) | 2259.14(1319.24-3699.72) |  | -32.23(-55.39-64.26) | -2.81(-3.11--2.51) |
| Denmark | 10112.00(8337.41-11429.05) | 3491.66(2878.89-3946.43) |  | 1907.46(1437.85-2316.20) | 613.93(462.79-745.49) |  | -81.14(-85.85--74.43) | -5.42(-5.94--4.90) |
| Djibouti | 2896.23(881.85-5894.75) | 4481.34(1364.49-9120.94) |  | 2999.49(1714.61-5398.75) | 2058.73(1176.84-3705.50) |  | 3.57(-39.37-168.38) | -2.34(-2.70--1.98) |
| Dominica | 211.27(162.19-271.09) | 2439.33(1872.70-3130.00) |  | 122.45(79.60-176.71) | 3520.21(2288.27-5080.14) |  | -42.04(-62.94--12.56) | 1.46(1.10-1.81) |
| Dominican Republic | 51249.94(39682.21-72943.59) | 5146.36(3984.76-7324.77) |  | 19973.65(10911.10-38709.88) | 1933.33(1056.13-3746.88) |  | -61.03(-78.60--22.51) | -2.56(-2.85--2.26) |
| Ecuador | 54412.32(45081.19-62989.21) | 4043.82(3350.35-4681.24) |  | 48229.34(36117.73-63522.83) | 2903.42(2174.29-3824.09) |  | -11.36(-35.82-20.30) | -0.06(-0.37-0.24) |
| Egypt | 1380734.26(468944.36-2113711.55) | 16181.40(5495.75-24771.47) |  | 431346.09(320242.49-573283.30) | 3308.53(2456.34-4397.22) |  | -68.76(-81.94--0.41) | -4.50(-4.85--4.15) |
| El Salvador | 68105.74(36108.00-91259.21) | 8843.96(4688.85-11850.58) |  | 13631.43(8596.99-20645.77) | 2269.15(1431.10-3436.79) |  | -79.98(-88.28--49.88) | -3.73(-4.02--3.43) |
| Equatorial Guinea | 4389.67(1471.24-8533.04) | 5336.33(1788.52-10373.24) |  | 3294.68(1947.68-5330.53) | 1758.68(1039.66-2845.40) |  | -24.94(-61.07-185.36) | -3.99(-4.20--3.77) |
| Eritrea | 31989.31(7913.40-64958.08) | 5130.65(1269.20-10418.39) |  | 27374.66(14498.89-52746.05) | 2981.87(1579.34-5745.53) |  | -14.43(-48.57-128.40) | -1.47(-1.64--1.30) |
| Estonia | 3423.27(3009.23-3827.51) | 2838.40(2495.10-3173.57) |  | 259.98(169.98-362.62) | 375.82(245.72-524.20) |  | -92.41(-95.34--88.60) | -6.93(-7.68--6.17) |
| Eswatini | 3072.89(1844.78-4276.08) | 2113.19(1268.63-2940.61) |  | 2223.33(1484.08-3339.44) | 1583.39(1056.91-2378.24) |  | -27.65(-54.21-46.92) | -0.52(-0.83--0.21) |
| Ethiopia | 736347.89(175055.78-1508933.14) | 7639.01(1816.06-15653.95) |  | 448060.81(253804.89-804559.03) | 2806.50(1589.75-5039.48) |  | -39.15(-61.39-68.68) | -3.33(-3.46--3.21) |
| Fiji | 4125.51(3120.32-5278.41) | 4377.68(3311.05-5601.05) |  | 3635.37(2542.23-5075.04) | 3992.93(2792.26-5574.19) |  | -11.88(-41.07-37.16) | -0.19(-0.51-0.12) |
| Finland | 6956.42(5968.84-7855.28) | 2224.80(1908.95-2512.27) |  | 1244.33(891.96-1611.78) | 512.50(367.37-663.83) |  | -82.11(-86.60--76.18) | -4.86(-5.10--4.62) |
| France | 80728.95(66452.92-86701.45) | 2075.62(1708.57-2229.18) |  | 18902.94(14533.74-24984.79) | 534.90(411.26-707.00) |  | -76.58(-81.97--64.41) | -4.15(-4.43--3.87) |
| Gabon | 5189.88(2504.96-9606.75) | 3322.79(1603.79-6150.66) |  | 3188.69(2055.39-5364.97) | 1493.26(962.54-2512.41) |  | -38.56(-63.16-43.47) | -1.82(-2.24--1.39) |
| Gambia | 9087.02(2608.71-13874.60) | 4893.61(1404.86-7471.85) |  | 8388.00(5562.19-11960.32) | 2345.71(1555.47-3344.71) |  | -7.69(-45.10-163.75) | -2.51(-2.73--2.29) |
| Georgia | 10033.76(7431.81-12500.38) | 2141.39(1586.08-2667.81) |  | 4357.31(3176.30-5702.42) | 1790.97(1305.54-2343.85) |  | -56.57(-67.64--40.97) | 0.37(-0.13-0.87) |
| Germany | 94710.22(79749.68-109547.05) | 2114.44(1780.44-2445.68) |  | 25427.98(19975.03-30209.31) | 628.87(494.01-747.12) |  | -73.15(-79.40--66.58) | -3.77(-4.08--3.45) |
| Ghana | 120731.95(38672.66-189975.72) | 4583.57(1468.20-7212.40) |  | 102728.01(62758.45-147910.84) | 2213.90(1352.51-3187.64) |  | -14.91(-48.56-159.51) | -1.75(-1.96--1.55) |
| Greece | 22304.81(18646.11-25033.18) | 4019.09(3359.84-4510.72) |  | 3769.82(2954.50-4571.40) | 892.03(699.10-1081.70) |  | -83.10(-87.25--76.60) | -4.70(-5.17--4.24) |
| Greenland | 183.37(98.31-271.43) | 3329.84(1785.31-4929.05) |  | 28.76(19.10-49.08) | 711.63(472.53-1214.41) |  | -84.31(-91.86--68.85) | -5.04(-5.30--4.78) |
| Grenada | 439.20(354.65-544.80) | 3657.38(2953.29-4536.77) |  | 180.51(139.67-230.14) | 2628.56(2033.83-3351.25) |  | -58.90(-68.03--45.85) | -0.64(-0.89--0.39) |
| Guam | 280.18(222.20-366.34) | 1732.38(1373.88-2265.11) |  | 177.86(128.59-248.38) | 1391.82(1006.29-1943.68) |  | -36.52(-54.79--15.02) | 0.39(-0.12-0.91) |
| Guatemala | 23490.30(19447.99-29978.14) | 1531.28(1267.77-1954.21) |  | 47518.15(34128.39-62622.30) | 3047.47(2188.75-4016.14) |  | 102.29(40.60-186.98) | 3.89(3.05-4.74) |
| Guinea | 128264.88(25966.48-222434.15) | 11052.16(2237.45-19166.42) |  | 118832.66(66693.67-177392.38) | 5280.33(2963.54-7882.44) |  | -7.35(-38.79-187.21) | -1.97(-2.15--1.79) |
| Guinea-Bissau | 16615.84(3226.41-27457.34) | 8834.63(1715.48-14599.04) |  | 10880.44(6730.52-15568.03) | 3270.78(2023.27-4679.93) |  | -34.52(-62.21-138.31) | -2.96(-3.30--2.62) |
| Guyana | 4258.85(3498.62-5148.48) | 3775.52(3101.57-4564.19) |  | 1964.32(1392.17-2687.93) | 2636.23(1868.37-3607.36) |  | -53.88(-68.35--33.68) | -0.12(-0.51-0.27) |
| Haiti | 195130.70(126686.37-277827.44) | 18396.38(11943.64-26192.80) |  | 157813.19(89050.11-261677.93) | 10052.78(5672.53-16669.01) |  | -19.12(-44.61-40.30) | -1.64(-1.89--1.40) |
| Honduras | 56076.30(32831.62-79026.36) | 6813.60(3989.24-9602.17) |  | 25749.15(17867.62-38026.65) | 2350.40(1630.97-3471.10) |  | -54.08(-71.69--2.95) | -3.21(-3.32--3.10) |
| Hungary | 21621.90(17846.70-24793.83) | 3492.03(2882.32-4004.31) |  | 3370.92(2469.27-4279.28) | 741.83(543.40-941.73) |  | -84.41(-89.46--78.59) | -4.71(-4.95--4.48) |
| Iceland | 385.19(316.75-437.87) | 1823.25(1499.27-2072.61) |  | 112.31(80.49-146.76) | 511.14(366.33-667.93) |  | -70.84(-78.51--58.12) | -4.07(-4.43--3.70) |
| India | 6386241.44(3894988.24-8669444.68) | 5493.80(3350.69-7457.94) |  | 3006729.23(2137601.21-4357783.78) | 2700.58(1919.95-3914.07) |  | -52.92(-68.96-8.57) | -1.94(-2.09--1.80) |
| Indonesia | 1289430.21(661483.04-1759735.33) | 5776.81(2963.53-7883.84) |  | 596215.76(420853.33-803642.96) | 2721.63(1921.13-3668.50) |  | -53.76(-69.59-11.03) | -2.53(-2.62--2.45) |
| Iran (Islamic Republic of) | 1130909.48(601394.98-1547087.12) | 12892.09(6855.76-17636.42) |  | 59260.36(40670.75-83668.50) | 962.78(660.76-1359.33) |  | -94.76(-97.16--87.44) | -5.69(-6.74--4.63) |
| Iraq | 381801.80(196990.09-526503.27) | 12148.70(6268.10-16753.01) |  | 151340.18(106292.97-212290.46) | 3524.92(2475.71-4944.54) |  | -60.36(-76.61--4.50) | -3.94(-4.20--3.68) |
| Ireland | 5299.26(4554.03-5818.01) | 1833.79(1575.90-2013.30) |  | 1576.01(1238.54-1973.73) | 528.15(415.06-661.44) |  | -70.26(-76.68--61.10) | -4.15(-4.42--3.87) |
| Israel | 13065.61(10372.54-15102.15) | 2530.32(2008.77-2924.72) |  | 4729.57(3620.34-5860.22) | 515.05(394.25-638.18) |  | -63.80(-74.13--49.54) | -4.45(-4.72--4.18) |
| Italy | 65814.32(56838.42-73661.85) | 2396.97(2070.06-2682.78) |  | 12947.68(9779.51-15878.81) | 596.63(450.64-731.70) |  | -80.33(-86.37--74.98) | -4.86(-5.03--4.68) |
| Jamaica | 8842.07(6636.29-10967.45) | 3169.10(2378.52-3930.86) |  | 3223.64(2327.76-4396.25) | 1883.17(1359.82-2568.18) |  | -63.54(-75.28--44.54) | -1.03(-1.40--0.65) |
| Japan | 148574.93(131324.66-161545.70) | 2231.43(1972.35-2426.23) |  | 23727.70(17132.10-31149.32) | 517.46(373.62-679.31) |  | -84.03(-88.44--77.61) | -4.48(-4.66--4.30) |
| Jordan | 54825.68(37882.78-69149.24) | 9134.37(6311.55-11520.78) |  | 29992.79(23103.28-41142.91) | 2733.29(2105.44-3749.41) |  | -45.29(-63.08--1.28) | -3.84(-3.94--3.74) |
| Kazakhstan | 70877.39(53251.91-90224.26) | 3764.27(2828.19-4791.78) |  | 53707.12(42628.13-67643.85) | 2756.42(2187.81-3471.69) |  | -24.23(-44.89-5.33) | -1.07(-2.18-0.05) |
| Kenya | 120227.47(49538.89-254105.65) | 2800.55(1153.95-5919.08) |  | 79769.77(45395.96-153677.78) | 1340.63(762.93-2582.74) |  | -33.65(-59.53-54.72) | -1.83(-2.07--1.59) |
| Kiribati | 959.51(233.21-1517.45) | 8147.89(1980.32-12885.79) |  | 587.60(237.83-867.52) | 4111.25(1664.00-6069.77) |  | -38.76(-55.39-6.17) | -2.13(-2.25--2.01) |
| Kuwait | 10046.25(8229.71-12398.25) | 4913.79(4025.29-6064.19) |  | 4837.54(3856.06-5994.65) | 1825.39(1455.04-2262.01) |  | -51.85(-65.76--33.79) | -3.18(-3.87--2.49) |
| Kyrgyzstan | 21965.70(17752.26-25804.35) | 3417.49(2761.95-4014.72) |  | 23023.00(18225.18-28121.90) | 2899.99(2295.65-3542.25) |  | 4.81(-24.45-38.87) | 0.27(-0.36-0.91) |
| Lao People's Democratic Republic | 123460.86(26044.81-192801.59) | 17365.49(3663.36-27118.67) |  | 62087.84(35732.90-90895.43) | 7484.67(4307.59-10957.42) |  | -49.71(-65.73-54.88) | -2.80(-2.95--2.64) |
| Latvia | 8440.13(7410.03-9961.25) | 4165.16(3656.81-4915.83) |  | 517.19(401.06-706.40) | 551.88(427.96-753.78) |  | -93.87(-95.80--91.12) | -6.31(-7.12--5.49) |
| Lebanon | 18931.57(8388.87-28232.94) | 4859.08(2153.13-7246.42) |  | 4742.10(3213.35-7167.41) | 1168.29(791.66-1765.81) |  | -74.95(-85.76--42.22) | -4.82(-5.13--4.50) |
| Lesotho | 5856.63(3159.18-9086.06) | 2377.81(1282.64-3688.97) |  | 4248.50(2442.92-6835.96) | 2085.52(1199.19-3355.66) |  | -27.46(-53.54-18.42) | -0.47(-0.60--0.34) |
| Liberia | 57112.46(9627.90-100623.97) | 12297.48(2073.08-21666.39) |  | 27739.93(15902.58-42474.78) | 3619.57(2075.00-5542.20) |  | -51.43(-68.06-88.87) | -3.77(-4.17--3.37) |
| Libya | 65780.37(41065.03-90005.76) | 10327.86(6447.42-14131.38) |  | 21317.33(13746.23-30991.97) | 5038.66(3249.12-7325.41) |  | -67.59(-77.90--50.26) | -1.74(-2.28--1.21) |
| Lithuania | 10664.09(9538.36-12291.60) | 3689.01(3299.59-4252.01) |  | 1029.22(752.38-1311.02) | 782.12(571.75-996.26) |  | -90.35(-93.56--87.46) | -4.93(-5.45--4.41) |
| Luxembourg | 320.54(268.97-369.87) | 1399.91(1174.72-1615.35) |  | 109.32(81.58-153.78) | 330.62(246.72-465.07) |  | -65.90(-74.99--48.97) | -4.55(-5.33--3.77) |
| Madagascar | 118404.98(33212.86-240125.24) | 5492.91(1540.77-11139.61) |  | 109343.19(58115.08-192987.01) | 2674.70(1421.58-4720.75) |  | -7.65(-38.64-95.80) | -1.80(-1.99--1.61) |
| Malawi | 165946.20(36750.45-322710.78) | 8739.86(1935.53-16996.15) |  | 73554.16(43714.00-122988.34) | 2700.17(1604.74-4514.90) |  | -55.68(-73.82-60.35) | -3.48(-3.63--3.33) |
| Malaysia | 59768.58(36487.23-79213.77) | 2508.82(1531.57-3325.04) |  | 25912.83(19169.65-33795.76) | 1054.05(779.76-1374.70) |  | -56.64(-70.44--15.01) | -2.08(-2.71--1.44) |
| Maldives | 2640.79(778.65-4009.99) | 6320.47(1863.62-9597.53) |  | 572.47(411.44-809.31) | 1802.60(1295.54-2548.35) |  | -78.32(-88.30--15.11) | -3.59(-3.77--3.40) |
| Mali | 242546.68(110801.45-414594.28) | 14026.75(6407.77-23976.45) |  | 273851.56(158435.13-398392.04) | 5978.80(3459.00-8697.79) |  | 12.91(-27.91-181.55) | -2.54(-2.68--2.40) |
| Malta | 695.71(530.99-800.20) | 2463.23(1880.04-2833.18) |  | 221.79(173.13-280.15) | 1008.02(786.88-1273.26) |  | -68.12(-75.65--58.33) | -2.11(-2.58--1.64) |
| Marshall Islands | 239.09(121.96-326.91) | 3214.76(1639.88-4395.52) |  | 132.14(86.33-196.79) | 2329.15(1521.69-3468.66) |  | -44.73(-65.06--6.71) | -1.14(-1.57--0.72) |
| Mauritania | 16456.72(4418.77-24671.00) | 4436.43(1191.22-6650.85) |  | 12667.35(8646.78-17660.92) | 1928.05(1316.10-2688.11) |  | -23.03(-52.32-130.21) | -2.62(-2.98--2.26) |
| Mauritius | 3529.43(3010.81-4483.59) | 3346.39(2854.67-4251.07) |  | 1623.93(1292.83-2169.62) | 2525.74(2010.76-3374.45) |  | -53.99(-63.83--43.27) | -1.20(-1.50--0.91) |
| Mexico | 491213.91(427194.85-589918.24) | 4163.52(3620.90-5000.14) |  | 355044.01(263738.56-463647.81) | 3594.61(2670.20-4694.16) |  | -27.72(-49.05--0.34) | -0.47(-0.79--0.15) |
| Micronesia (Federated States of) | 716.12(291.62-1048.38) | 4573.23(1862.28-6695.06) |  | 169.29(121.17-237.87) | 1787.60(1279.50-2511.75) |  | -76.36(-85.21--45.27) | -3.04(-3.13--2.94) |
| Monaco | 20.40(14.27-27.98) | 1741.79(1218.36-2389.70) |  | 12.16(8.61-17.11) | 751.51(532.19-1057.45) |  | -40.38(-62.37--4.57) | -3.89(-4.45--3.32) |
| Mongolia | 22468.96(9869.76-33289.07) | 6617.73(2906.92-9804.55) |  | 8587.53(5907.66-11469.08) | 2198.01(1512.09-2935.55) |  | -61.78(-78.92--1.94) | -3.41(-3.63--3.19) |
| Montenegro | 1097.88(778.27-1477.53) | 2092.61(1483.42-2816.25) |  | 139.43(91.72-218.48) | 385.26(253.42-603.67) |  | -87.30(-92.15--75.72) | -5.51(-6.03--4.99) |
| Morocco | 239408.46(167507.40-323307.74) | 6724.23(4704.76-9080.70) |  | 48627.25(28569.74-98219.15) | 1495.86(878.86-3021.39) |  | -79.69(-89.07--56.63) | -4.60(-4.91--4.28) |
| Mozambique | 236889.41(52826.66-456260.29) | 9833.50(2192.88-18939.80) |  | 192063.16(102624.18-344154.44) | 3708.34(1981.46-6644.91) |  | -18.92(-48.55-121.25) | -2.73(-2.87--2.59) |
| Myanmar | 796628.90(214706.60-1279366.63) | 15799.17(4258.18-25373.09) |  | 405476.03(236816.96-574973.18) | 7758.21(4531.16-11001.30) |  | -49.10(-66.28-31.16) | -2.49(-2.77--2.21) |
| Namibia | 4364.53(2757.79-6139.93) | 1928.15(1218.32-2712.47) |  | 3509.35(2268.62-5340.35) | 1260.31(814.73-1917.87) |  | -19.59(-50.59-53.20) | -0.80(-1.05--0.55) |
| Nauru | 69.09(33.66-99.66) | 4228.65(2059.81-6099.36) |  | 45.80(28.37-67.94) | 3276.39(2029.50-4860.28) |  | -33.71(-55.39-3.94) | -0.89(-1.53--0.24) |
| Nepal | 157889.93(108437.13-213819.07) | 4794.16(3292.58-6492.40) |  | 43674.54(23407.80-95687.47) | 1405.96(753.54-3080.34) |  | -72.34(-86.00--34.32) | -3.80(-3.89--3.71) |
| Netherlands | 19248.70(15935.29-21481.70) | 2055.79(1701.91-2294.28) |  | 4419.05(3660.21-5649.90) | 513.11(425.00-656.03) |  | -77.04(-81.68--67.67) | -4.64(-4.92--4.36) |
| New Zealand | 4269.95(3778.85-4792.45) | 1526.91(1351.29-1713.75) |  | 1591.51(1202.09-2127.66) | 509.12(384.55-680.64) |  | -62.73(-71.39--52.18) | -3.55(-3.93--3.18) |
| Nicaragua | 47598.46(24053.68-69289.88) | 7173.75(3625.22-10442.95) |  | 14112.43(9477.50-20608.71) | 2169.36(1456.88-3167.97) |  | -70.35(-83.79--28.24) | -3.31(-3.48--3.13) |
| Niger | 164453.40(24402.89-284372.55) | 9748.34(1446.53-16856.81) |  | 219569.11(91681.18-339906.20) | 4308.37(1798.96-6669.61) |  | 33.51(-9.38-322.01) | -2.75(-2.99--2.51) |
| Nigeria | 1135484.61(249722.58-1766760.19) | 7143.57(1571.06-11115.06) |  | 1791050.29(882748.92-2764790.19) | 4825.31(2378.23-7448.68) |  | 57.73(14.10-278.50) | -0.82(-0.98--0.66) |
| Niue | 7.65(5.01-10.53) | 2998.20(1965.43-4127.72) |  | 9.43(7.39-12.02) | 8029.61(6294.88-10237.43) |  | 23.31(-9.00-82.76) | 0.57(-0.35-1.50) |
| North Macedonia | 11256.23(8033.57-14240.22) | 6608.07(4716.18-8359.84) |  | 773.94(565.60-1104.14) | 769.96(562.68-1098.45) |  | -93.12(-95.72--86.81) | -5.69(-6.14--5.24) |
| Northern Mariana Islands | 62.61(44.28-89.34) | 1317.39(931.64-1879.93) |  | 22.66(15.95-31.57) | 706.66(497.28-984.43) |  | -63.80(-72.37--48.90) | -1.21(-1.65--0.78) |
| Norway | 5724.29(5267.95-6303.17) | 2075.12(1909.69-2284.97) |  | 1088.31(742.31-1761.99) | 387.14(264.06-626.78) |  | -80.99(-87.20--68.59) | -5.20(-5.49--4.90) |
| Oman | 21327.21(12365.58-30739.71) | 6508.61(3773.71-9381.10) |  | 6628.54(5131.94-8848.03) | 1563.25(1210.30-2086.69) |  | -68.92(-80.80--34.83) | -3.46(-4.29--2.61) |
| Pakistan | 994277.43(681733.45-1329512.01) | 5385.68(3692.73-7201.54) |  | 925109.72(558089.36-1347865.49) | 3112.12(1877.45-4534.30) |  | -6.96(-37.09-60.58) | -0.90(-1.27--0.53) |
| Palau | 59.31(30.64-88.91) | 4015.97(2074.41-6019.99) |  | 19.22(13.42-25.94) | 2029.68(1417.19-2739.15) |  | -67.59(-78.10--43.96) | -1.81(-2.02--1.61) |
| Palestine | 37543.98(23453.74-51039.24) | 9626.82(6013.88-13087.20) |  | 14001.35(9982.95-19747.49) | 2284.98(1629.19-3222.73) |  | -62.71(-78.21--31.90) | -4.34(-4.63--4.05) |
| Panama | 16193.20(13581.05-19444.52) | 5670.75(4756.00-6809.34) |  | 14411.48(10866.14-18468.84) | 3882.06(2927.04-4975.00) |  | -11.00(-34.87-23.19) | -0.98(-1.14--0.82) |
| Papua New Guinea | 63676.70(16418.98-99111.70) | 9831.28(2534.99-15302.22) |  | 121735.05(41833.46-189362.46) | 8001.98(2749.83-12447.31) |  | 91.18(41.14-196.88) | -0.53(-0.71--0.34) |
| Paraguay | 24195.86(17722.85-34801.13) | 3921.44(2872.36-5640.25) |  | 16795.69(10573.60-25421.40) | 2583.68(1626.53-3910.57) |  | -30.58(-63.67-28.15) | -1.02(-1.25--0.79) |
| Peru | 227389.27(115842.77-308219.19) | 7771.39(3959.11-10533.88) |  | 70159.19(42998.21-101512.28) | 2125.72(1302.78-3075.67) |  | -69.15(-83.35--18.37) | -3.37(-3.68--3.05) |
| Philippines | 558018.75(324228.12-798343.05) | 6031.89(3504.73-8629.66) |  | 325810.08(248042.04-445055.21) | 2905.55(2212.02-3968.97) |  | -41.61(-58.60-5.29) | -1.93(-2.09--1.77) |
| Poland | 136359.50(112606.98-157145.00) | 4671.74(3857.97-5383.86) |  | 20704.94(15404.22-25551.42) | 1100.11(818.47-1357.62) |  | -84.82(-89.77--80.14) | -4.65(-4.99--4.32) |
| Portugal | 19988.73(16826.78-23080.81) | 3455.76(2909.10-3990.33) |  | 2445.16(1817.76-2970.58) | 574.89(427.38-698.42) |  | -87.77(-91.69--84.13) | -6.11(-6.54--5.66) |
| Puerto Rico | 7700.93(6648.69-8677.45) | 2412.61(2082.95-2718.54) |  | 1314.42(1015.97-1611.56) | 1250.38(966.47-1533.04) |  | -82.93(-86.76--78.54) | -2.49(-2.90--2.09) |
| Qatar | 2292.60(1438.02-3180.35) | 4512.54(2830.46-6259.92) |  | 1597.05(1107.78-2215.86) | 866.54(601.07-1202.31) |  | -30.34(-55.02-32.26) | -4.90(-5.04--4.75) |
| Republic of Korea | 113226.39(70014.40-148741.58) | 3411.37(2109.44-4481.39) |  | 7190.31(5352.17-10196.25) | 463.92(345.32-657.87) |  | -93.65(-96.21--87.23) | -5.95(-6.14--5.77) |
| Republic of Moldova | 22440.66(15978.08-29488.10) | 5208.40(3708.46-6844.09) |  | 3168.69(2325.21-4240.67) | 2054.62(1507.69-2749.70) |  | -85.88(-90.10--79.54) | -2.78(-3.24--2.31) |
| Romania | 91316.51(76256.81-109514.33) | 5160.48(4309.43-6188.88) |  | 14078.17(11725.32-16824.68) | 1501.11(1250.23-1793.96) |  | -84.58(-88.68--80.35) | -3.87(-4.37--3.36) |
| Russian Federation | 358316.34(316886.65-445428.11) | 3082.48(2726.08-3831.88) |  | 67701.44(51225.11-84996.18) | 889.56(673.07-1116.81) |  | -81.11(-87.54--75.06) | -4.26(-5.18--3.34) |
| Rwanda | 87625.61(21650.75-165933.44) | 6501.07(1606.30-12310.85) |  | 44771.91(27096.45-72652.04) | 2560.64(1549.73-4155.20) |  | -48.91(-70.85-50.20) | -3.33(-3.67--2.99) |
| Saint Kitts and Nevis | 165.18(140.48-193.01) | 3525.29(2998.21-4119.29) |  | 64.07(48.19-83.92) | 2099.58(1579.37-2750.15) |  | -61.21(-71.24--47.42) | -1.32(-1.52--1.12) |
| Saint Lucia | 536.54(427.90-649.68) | 3038.43(2423.22-3679.13) |  | 161.57(114.21-222.24) | 1829.11(1292.99-2515.89) |  | -69.89(-79.81--56.01) | -1.02(-1.32--0.72) |
| Saint Vincent and the Grenadines | 499.49(401.18-619.41) | 3909.14(3139.74-4847.62) |  | 114.78(84.42-152.56) | 1594.93(1173.15-2119.94) |  | -77.02(-84.27--67.63) | -2.69(-3.08--2.29) |
| Samoa | 921.83(512.48-1314.48) | 3616.34(2010.45-5156.74) |  | 506.34(344.58-725.61) | 1731.45(1178.30-2481.23) |  | -45.07(-67.51-10.94) | -2.16(-2.24--2.09) |
| San Marino | 10.23(6.80-14.82) | 862.41(573.25-1249.38) |  | 2.09(1.30-3.31) | 173.15(107.69-273.48) |  | -79.54(-88.14--60.19) | -4.87(-5.10--4.65) |
| Sao Tome and Principe | 1249.80(370.38-1931.85) | 6102.38(1808.47-9432.62) |  | 358.97(214.94-610.04) | 1439.09(861.70-2445.63) |  | -71.28(-87.29-33.93) | -4.46(-4.83--4.09) |
| Saudi Arabia | 218459.47(113543.61-317378.41) | 9035.85(4696.36-13127.31) |  | 18090.33(10551.76-29903.32) | 743.51(433.67-1229.01) |  | -91.72(-96.13--77.84) | -7.73(-7.81--7.65) |
| Senegal | 98615.85(24118.30-157739.27) | 6731.21(1646.24-10766.79) |  | 64241.68(45188.79-88108.32) | 2828.23(1989.43-3878.95) |  | -34.86(-61.11-126.55) | -2.40(-2.73--2.07) |
| Serbia | 45103.00(28439.01-58453.68) | 6625.75(4177.77-8587.00) |  | 3311.04(2429.55-4512.84) | 898.13(659.02-1224.12) |  | -92.66(-95.35--86.47) | -7.00(-7.72--6.28) |
| Seychelles | 262.85(212.06-327.86) | 3261.93(2631.57-4068.66) |  | 232.70(169.43-306.91) | 2955.16(2151.68-3897.48) |  | -11.47(-36.07-22.68) | 0.21(-0.01-0.44) |
| Sierra Leone | 100757.44(17220.47-172164.75) | 13021.18(2225.45-22249.36) |  | 80196.94(39174.73-125464.85) | 5971.69(2917.06-9342.47) |  | -20.41(-49.49-161.10) | -2.49(-2.61--2.37) |
| Singapore | 8667.88(7308.89-9635.77) | 4248.04(3582.01-4722.39) |  | 1094.81(748.38-1559.93) | 382.90(261.74-545.56) |  | -87.37(-91.52--80.11) | -6.60(-7.20--6.00) |
| Slovakia | 12300.58(10103.19-14432.60) | 3006.21(2469.18-3527.27) |  | 3099.94(2445.42-4006.78) | 1083.40(854.65-1400.33) |  | -74.80(-81.33--66.89) | -3.02(-3.26--2.77) |
| Slovenia | 3030.74(2327.54-3420.10) | 2464.18(1892.43-2780.74) |  | 393.36(277.27-493.97) | 401.80(283.22-504.57) |  | -87.02(-91.25--80.68) | -5.85(-6.02--5.68) |
| Solomon Islands | 3251.59(1527.27-4701.00) | 5450.65(2560.16-7880.28) |  | 2744.65(1896.65-3879.30) | 2878.86(1989.40-4069.00) |  | -15.59(-43.15-66.96) | -2.01(-2.13--1.89) |
| Somalia | 99044.41(22476.71-213516.10) | 6410.48(1454.77-13819.47) |  | 159815.91(56204.29-341947.16) | 3870.51(1361.19-8281.46) |  | 61.36(11.54-182.82) | -1.13(-1.42--0.83) |
| South Africa | 105237.14(85196.92-139320.16) | 2157.26(1746.46-2855.93) |  | 69638.93(46688.63-102939.63) | 1403.64(941.05-2074.85) |  | -33.83(-57.23-4.64) | -0.96(-1.10--0.83) |
| South Sudan | 78900.25(17262.25-161311.28) | 7752.00(1696.03-15848.93) |  | 104978.10(36200.68-188804.78) | 6719.31(2317.09-12084.79) |  | 33.05(-11.38-145.85) | -0.13(-0.58-0.32) |
| Spain | 55119.53(47963.26-62103.96) | 2646.48(2302.89-2981.83) |  | 10427.62(7743.91-12517.23) | 566.33(420.58-679.82) |  | -81.08(-86.82--76.36) | -5.64(-5.95--5.34) |
| Sri Lanka | 58165.32(37318.24-84349.50) | 3290.19(2110.95-4771.33) |  | 24770.81(17585.78-35629.03) | 1582.19(1123.26-2275.73) |  | -57.41(-73.01--14.99) | -2.01(-2.40--1.61) |
| Sudan | 995512.20(280269.57-1667626.51) | 28599.37(8051.67-47908.07) |  | 472670.87(307573.97-666602.87) | 8382.07(5454.34-11821.14) |  | -52.52(-69.72-25.03) | -3.63(-3.85--3.41) |
| Suriname | 1999.11(1474.69-2575.84) | 4563.80(3366.58-5880.43) |  | 1167.61(753.02-1697.21) | 2621.65(1690.77-3810.76) |  | -41.59(-62.55--11.12) | -1.79(-1.96--1.63) |
| Sweden | 11803.21(10026.93-13127.22) | 2096.27(1780.80-2331.42) |  | 2107.34(1550.38-3068.27) | 361.34(265.84-526.12) |  | -82.15(-86.91--70.85) | -4.77(-5.25--4.29) |
| Switzerland | 13190.72(10769.00-14795.34) | 3311.59(2703.61-3714.44) |  | 3104.16(2520.93-3734.58) | 702.30(570.35-844.94) |  | -76.47(-81.57--69.23) | -4.58(-5.06--4.10) |
| Syrian Arab Republic | 252386.40(141693.78-340653.13) | 11701.12(6569.20-15793.33) |  | 25731.06(18620.73-35547.27) | 2560.12(1852.68-3536.79) |  | -89.80(-93.45--76.57) | -3.78(-4.52--3.05) |
| Taiwan (Province of China) | 38436.73(35510.10-41009.85) | 2392.64(2210.46-2552.82) |  | 7163.89(5849.89-8501.07) | 802.92(655.65-952.79) |  | -81.36(-85.02--77.14) | -3.84(-4.24--3.44) |
| Tajikistan | 26298.04(20002.75-38724.20) | 2781.28(2115.49-4095.46) |  | 32769.03(19232.65-68269.89) | 2448.22(1436.90-5100.54) |  | 24.61(-35.41-183.69) | 0.39(0.13-0.64) |
| Thailand | 204339.79(139586.11-297955.75) | 3929.41(2684.21-5729.62) |  | 40914.94(27396.08-52953.65) | 1447.28(969.08-1873.12) |  | -79.98(-86.51--69.02) | -3.19(-3.40--2.99) |
| Timor-Leste | 17828.36(4621.69-28693.90) | 12730.01(3300.03-20488.35) |  | 10369.65(6607.07-14931.31) | 5609.81(3574.31-8077.58) |  | -41.84(-62.39-71.10) | -2.94(-3.15--2.74) |
| Togo | 39924.05(10758.25-60670.93) | 5817.31(1567.58-8840.32) |  | 32765.26(20983.53-47105.09) | 2794.46(1789.63-4017.46) |  | -17.93(-46.16-114.95) | -2.08(-2.22--1.94) |
| Tokelau | 6.35(3.61-8.74) | 3265.75(1857.78-4495.09) |  | 8.80(6.07-13.22) | 8888.55(6132.06-13353.98) |  | 38.57(-16.61-238.99) | -1.41(-3.22-0.44) |
| Tonga | 334.44(208.56-470.19) | 2189.54(1365.41-3078.27) |  | 170.36(116.02-268.69) | 1182.40(805.23-1864.86) |  | -49.06(-69.17--1.71) | -1.79(-2.04--1.55) |
| Trinidad and Tobago | 5073.40(4246.81-6047.19) | 3805.51(3185.49-4535.94) |  | 2532.67(1873.13-3361.78) | 3145.65(2326.48-4175.43) |  | -50.08(-64.99--27.09) | -0.53(-0.98--0.07) |
| Tunisia | 138297.20(63358.73-196728.87) | 12959.40(5937.15-18434.85) |  | 20477.15(15230.69-26764.85) | 2295.58(1707.42-3000.45) |  | -85.19(-90.90--67.15) | -5.08(-5.24--4.93) |
| Turkey | 1283589.36(570917.71-1980750.32) | 18455.50(8208.68-28479.30) |  | 145667.14(108131.85-188136.77) | 2623.75(1947.67-3388.71) |  | -88.65(-93.69--71.84) | -6.01(-6.18--5.84) |
| Turkmenistan | 22771.70(17281.93-29136.66) | 3888.69(2951.21-4975.62) |  | 26506.93(18536.32-35139.78) | 4911.29(3434.47-6510.81) |  | 16.40(-12.67-56.79) | 2.81(1.91-3.73) |
| Tuvalu | 135.31(36.05-213.78) | 8968.57(2389.74-14169.80) |  | 29.75(19.08-43.47) | 2318.06(1486.56-3387.14) |  | -78.01(-87.50--31.81) | -4.05(-4.25--3.86) |
| Uganda | 201713.18(54040.26-393547.85) | 5616.46(1504.69-10957.86) |  | 209918.75(124418.46-346321.31) | 2868.64(1700.24-4732.64) |  | 4.07(-30.99-172.65) | -1.86(-2.06--1.65) |
| Ukraine | 160603.83(137814.35-190130.30) | 4261.23(3656.57-5044.65) |  | 24873.34(19846.73-30730.82) | 1562.20(1246.50-1930.08) |  | -84.51(-88.24--79.99) | -3.35(-3.80--2.91) |
| United Arab Emirates | 14079.57(9323.15-19355.11) | 6176.67(4090.04-8491.03) |  | 4436.75(2644.77-6235.59) | 1025.42(611.26-1441.16) |  | -68.49(-79.96--38.53) | -4.01(-4.58--3.43) |
| United Kingdom | 73688.55(69571.90-79866.21) | 1918.24(1811.08-2079.06) |  | 23263.10(19056.63-28580.36) | 636.54(521.44-782.03) |  | -68.43(-74.06--61.40) | -3.02(-3.34--2.69) |
| United Republic of Tanzania | 371695.71(90082.30-704622.86) | 7730.74(1873.58-14655.14) |  | 306082.06(167588.89-536570.27) | 3460.05(1894.48-6065.57) |  | -17.65(-49.17-132.95) | -2.10(-2.29--1.90) |
| United States of America | 381202.84(328714.11-416695.84) | 1930.07(1664.31-2109.77) |  | 128745.71(106500.84-158601.74) | 692.40(572.77-852.97) |  | -66.23(-72.94--55.29) | -2.83(-3.01--2.65) |
| United States Virgin Islands | 332.64(252.10-413.89) | 3021.77(2290.14-3759.89) |  | 33.76(20.74-56.49) | 861.45(529.22-1441.27) |  | -89.85(-94.00--80.92) | -3.76(-3.99--3.53) |
| Uruguay | 10404.21(8514.55-12305.64) | 3813.33(3120.74-4510.24) |  | 3445.33(2646.46-4356.13) | 1775.93(1364.14-2245.41) |  | -66.89(-76.89--56.81) | -2.39(-2.66--2.12) |
| Uzbekistan | 96857.93(80205.86-113449.60) | 2871.57(2377.88-3363.47) |  | 190149.35(134556.72-249029.38) | 4957.95(3508.43-6493.18) |  | 96.32(41.30-163.67) | 2.56(1.85-3.27) |
| Vanuatu | 1092.11(435.47-1567.16) | 4038.89(1610.47-5795.71) |  | 994.86(615.19-1390.29) | 2360.20(1459.47-3298.31) |  | -8.90(-37.93-64.97) | -1.73(-2.15--1.32) |
| Venezuela (Bolivarian Republic of) | 85493.42(76207.50-94340.20) | 3378.13(3011.21-3727.70) |  | 73376.63(51139.23-99569.65) | 3358.30(2340.54-4557.10) |  | -14.17(-40.37-18.04) | 0.55(0.38-0.71) |
| Viet Nam | 275726.94(114323.72-380853.06) | 2930.35(1215.00-4047.60) |  | 95583.45(62993.32-141678.41) | 1173.99(773.70-1740.14) |  | -65.33(-79.24--10.84) | -2.53(-2.85--2.21) |
| Yemen | 696179.99(192091.82-1127580.64) | 25073.26(6918.28-40610.37) |  | 437373.23(275322.18-603398.06) | 9311.15(5861.28-12845.62) |  | -37.18(-58.62-53.00) | -3.29(-3.43--3.14) |
| Zambia | 107750.37(28189.52-207030.45) | 7139.72(1867.89-13718.19) |  | 79813.03(48243.54-139833.48) | 2728.62(1649.33-4780.57) |  | -25.93(-63.11-212.33) | -2.74(-2.99--2.48) |
| Zimbabwe | 32115.43(22676.68-42187.69) | 1821.09(1285.87-2392.23) |  | 40975.66(27546.55-58609.58) | 1854.34(1246.61-2652.36) |  | 27.59(-6.91-93.16) | 0.44(0.22-0.65) |
