# Supplementary material for: Global, regional, and national epidemiology of congenital heart disease in children from 1990 to 2021
Source: Front Cardiovasc Med. 2025 May 16;12:1522644. doi: 10.3389/fcvm.2025.1522644 (PMC12122482; doi:10.3389/fcvm.2025.1522644)
Supplement: Supplementary file 8 [file Table5.docx]

Table S5. Prevalence of Childhood Congenital Heart Disease at the National Level

| location | 1990 | |  | 2021 | |  | 1990-2021 | |
| --- | --- | --- | --- | --- | --- | --- | --- | --- |
|  | prevalence case | prevalence rate |  | prevalence case | prevalence rate |  | Cases change | EAPC |
| Afghanistan | 13933.57(12017.21-16335.34) | 812.85(701.05-952.96) |  | 44756.92(38799.66-53911.71) | 816.52(707.84-983.53) |  | 221.22(201.28-242.56) | -0.01(-0.03-0.01) |
| Albania | 3040.46(2554.06-3592.18) | 753.02(632.55-889.66) |  | 1059.80(909.33-1255.86) | 743.18(637.67-880.67) |  | -65.14(-67.85--62.47) | -0.03(-0.07-0.02) |
| Algeria | 24136.38(20978.49-27426.63) | 646.05(561.53-734.12) |  | 31467.51(27150.29-35799.93) | 668.86(577.09-760.94) |  | 30.37(23.13-37.74) | 0.14(0.11-0.17) |
| American Samoa | 36.56(31.93-42.32) | 479.71(418.94-555.20) |  | 17.58(15.31-20.10) | 473.89(412.84-542.02) |  | -51.93(-54.53--49.35) | -0.08(-0.11--0.06) |
| Andorra | 16.65(14.60-18.86) | 614.95(539.20-696.75) |  | 15.25(13.35-17.33) | 604.07(528.63-686.56) |  | -8.39(-12.82--3.72) | -0.05(-0.07--0.02) |
| Angola | 15166.25(12651.57-18172.70) | 776.89(648.08-930.90) |  | 39819.51(33495.60-48498.46) | 706.87(594.61-860.94) |  | 162.55(136.77-192.86) | -0.34(-0.38--0.31) |
| Antigua and Barbuda | 33.60(28.85-38.63) | 554.33(476.02-637.44) |  | 28.81(24.69-32.97) | 546.52(468.36-625.32) |  | -14.24(-18.49--9.43) | -0.07(-0.12--0.02) |
| Argentina | 17903.13(15408.27-20751.49) | 520.92(448.33-603.80) |  | 16694.82(14106.24-19350.36) | 557.19(470.79-645.82) |  | -6.75(-13.81-0.65) | 0.18(0.16-0.20) |
| Armenia | 4027.73(3371.69-4852.45) | 1051.83(880.50-1267.20) |  | 2069.77(1759.88-2436.76) | 1111.53(945.11-1308.62) |  | -48.61(-52.81--43.95) | 0.28(0.23-0.33) |
| Australia | 7390.18(6364.75-8518.22) | 585.30(504.09-674.64) |  | 8978.26(7632.00-10464.76) | 597.18(507.63-696.05) |  | 21.49(13.68-29.08) | 0.03(-0.03-0.10) |
| Austria | 3059.63(2817.85-3307.73) | 684.44(630.35-739.94) |  | 3617.72(3291.40-3942.99) | 837.21(761.69-912.48) |  | 18.24(12.04-24.39) | 0.51(0.35-0.67) |
| Azerbaijan | 8977.83(7588.40-10869.95) | 993.39(839.65-1202.75) |  | 7105.53(5942.48-8487.91) | 985.45(824.15-1177.17) |  | -20.85(-26.70--14.56) | 0.02(-0.03-0.06) |
| Bahamas | 132.15(114.95-150.23) | 518.18(450.74-589.07) |  | 109.79(94.48-125.47) | 521.45(448.74-595.90) |  | -16.92(-20.77--12.44) | -0.05(-0.09--0.02) |
| Bahrain | 353.63(306.79-401.18) | 575.78(499.52-653.21) |  | 544.58(472.03-620.45) | 581.26(503.82-662.23) |  | 54.00(45.34-62.78) | 0.05(0.03-0.07) |
| Bangladesh | 128028.81(106578.09-152797.31) | 676.29(562.98-807.13) |  | 93064.94(79440.84-111967.04) | 647.90(553.05-779.49) |  | -27.31(-33.25--20.90) | -0.13(-0.15--0.10) |
| Barbados | 109.79(94.02-125.80) | 560.67(480.14-642.41) |  | 78.47(67.42-89.91) | 576.27(495.09-660.24) |  | -28.53(-32.28--24.28) | 0.07(0.05-0.10) |
| Belarus | 6768.95(5736.92-7957.05) | 840.09(712.00-987.54) |  | 3790.51(3212.29-4560.41) | 811.05(687.33-975.78) |  | -44.00(-48.44--39.61) | -0.10(-0.13--0.08) |
| Belgium | 4011.50(3658.20-4365.35) | 672.50(613.28-731.82) |  | 3975.61(3528.86-4400.56) | 671.63(596.16-743.42) |  | -0.89(-7.32-5.41) | 0.00(-0.13-0.13) |
| Belize | 141.81(124.78-160.95) | 480.04(422.37-544.80) |  | 186.40(164.53-211.20) | 488.83(431.48-553.86) |  | 31.44(24.73-38.52) | 0.01(-0.05-0.06) |
| Benin | 7081.21(5907.94-8761.02) | 717.66(598.75-887.90) |  | 16069.39(13650.98-19092.45) | 686.03(582.79-815.09) |  | 126.93(107.68-145.33) | -0.12(-0.13--0.10) |
| Bermuda | 25.84(22.25-29.84) | 601.39(517.94-694.65) |  | 15.00(12.84-17.41) | 587.88(503.42-682.48) |  | -41.94(-45.01--38.77) | -0.09(-0.12--0.06) |
| Bhutan | 606.06(518.34-722.10) | 634.84(542.95-756.38) |  | 384.02(329.40-464.88) | 629.22(539.73-761.72) |  | -36.64(-42.13--31.12) | -0.04(-0.05--0.02) |
| Bolivia (Plurinational State of) | 5307.83(4700.80-5976.49) | 525.55(465.45-591.76) |  | 6203.28(5460.62-7094.52) | 519.46(457.27-594.09) |  | 16.87(11.37-22.57) | -0.04(-0.05--0.02) |
| Bosnia and Herzegovina | 2691.98(2386.07-3058.13) | 765.64(678.63-869.78) |  | 1147.59(977.92-1328.31) | 759.79(647.46-879.44) |  | -57.37(-61.14--53.39) | 0.01(-0.00-0.03) |
| Botswana | 1548.14(1337.84-1843.60) | 726.75(628.03-865.45) |  | 1672.38(1465.38-1937.56) | 710.14(622.25-822.75) |  | 8.03(0.60-16.53) | -0.03(-0.08-0.02) |
| Brazil | 87319.61(78051.80-97523.81) | 530.43(474.13-592.41) |  | 87983.02(78210.37-98566.50) | 531.38(472.35-595.30) |  | 0.76(-3.28-4.09) | 0.01(-0.07-0.10) |
| Brunei Darussalam | 231.95(204.69-267.80) | 672.60(593.55-776.54) |  | 204.52(178.71-237.25) | 661.98(578.43-767.91) |  | -11.83(-16.91--6.97) | -0.05(-0.08--0.03) |
| Bulgaria | 4678.89(4001.85-5453.16) | 871.60(745.48-1015.84) |  | 2737.47(2368.29-3202.47) | 904.41(782.44-1058.03) |  | -41.49(-46.12--37.35) | 0.35(0.27-0.44) |
| Burkina Faso | 14271.14(11892.14-17338.92) | 760.51(633.73-923.99) |  | 30436.63(25484.86-36459.29) | 741.19(620.60-887.85) |  | 113.27(97.46-131.14) | -0.10(-0.11--0.08) |
| Burundi | 6932.50(5841.71-8162.38) | 644.82(543.36-759.22) |  | 13341.10(11285.70-15861.91) | 617.53(522.39-734.22) |  | 92.44(78.36-107.47) | -0.13(-0.16--0.10) |
| Cabo Verde | 308.79(266.37-351.26) | 518.91(447.63-590.28) |  | 233.45(200.20-266.31) | 529.86(454.38-604.45) |  | -24.40(-29.15--20.40) | 0.05(0.02-0.08) |
| Cambodia | 11874.75(10087.43-14014.85) | 650.66(552.72-767.92) |  | 10638.50(9125.95-12474.37) | 608.42(521.92-713.41) |  | -10.41(-16.33--3.92) | -0.20(-0.22--0.19) |
| Cameroon | 14045.92(11693.45-17065.61) | 700.05(582.81-850.56) |  | 31923.60(27021.26-38617.08) | 656.36(555.57-793.98) |  | 127.28(109.75-143.89) | -0.18(-0.21--0.15) |
| Canada | 14977.60(13337.97-16692.78) | 778.04(692.86-867.13) |  | 11942.36(10020.63-13997.49) | 628.52(527.38-736.68) |  | -20.27(-27.82--12.76) | -0.88(-0.99--0.77) |
| Central African Republic | 4173.67(3476.38-5031.88) | 828.61(690.17-998.99) |  | 6772.04(5690.61-8215.54) | 807.31(678.39-979.39) |  | 62.26(49.06-77.31) | -0.08(-0.09--0.07) |
| Chad | 9818.32(8219.56-11787.24) | 789.94(661.31-948.35) |  | 27887.31(23222.80-33914.28) | 767.08(638.78-932.86) |  | 184.03(161.52-209.44) | -0.12(-0.14--0.10) |
| Chile | 8806.93(7561.05-10309.51) | 612.85(526.15-717.41) |  | 7130.16(6411.80-7900.26) | 655.37(589.34-726.15) |  | -19.04(-28.49--9.81) | 0.25(0.23-0.27) |
| China | 726313.09(619822.09-849826.88) | 649.62(554.37-760.09) |  | 429591.79(371813.44-487773.45) | 553.11(478.72-628.02) |  | -40.85(-44.17--37.25) | -0.34(-0.40--0.29) |
| Colombia | 23770.37(20699.55-27098.92) | 565.03(492.03-644.15) |  | 19805.27(17218.38-22737.37) | 575.27(500.13-660.44) |  | -16.68(-20.65--12.04) | 0.07(0.05-0.09) |
| Comoros | 452.10(384.68-538.32) | 548.55(466.75-653.17) |  | 428.31(373.10-502.78) | 526.78(458.88-618.38) |  | -5.26(-12.29-1.42) | -0.15(-0.16--0.13) |
| Congo | 2599.24(2207.51-3149.42) | 658.88(559.58-798.35) |  | 3894.40(3299.75-4624.63) | 615.38(521.42-730.77) |  | 49.83(39.49-60.58) | -0.26(-0.30--0.22) |
| Cook Islands | 10.43(9.05-11.93) | 465.76(403.90-532.49) |  | 5.05(4.39-5.73) | 449.57(390.07-509.29) |  | -51.55(-54.28--48.89) | -0.15(-0.19--0.12) |
| Costa Rica | 2313.15(2004.93-2670.27) | 569.78(493.86-657.75) |  | 1875.60(1606.28-2167.54) | 608.37(521.01-703.06) |  | -18.92(-23.30--14.64) | 0.21(0.19-0.23) |
| Croatia | 2073.11(1839.60-2315.40) | 688.11(610.60-768.53) |  | 1359.37(1175.33-1553.12) | 745.89(644.91-852.20) |  | -34.43(-39.99--27.60) | 0.53(0.42-0.65) |
| Cuba | 5292.06(4535.62-6074.62) | 591.25(506.74-678.68) |  | 3034.69(2616.55-3512.60) | 557.37(480.57-645.15) |  | -42.66(-45.56--39.23) | -0.25(-0.30--0.21) |
| Cyprus | 403.77(352.22-455.29) | 631.87(551.20-712.50) |  | 460.49(405.96-519.61) | 613.65(540.98-692.43) |  | 14.05(7.03-20.59) | -0.08(-0.10--0.06) |
| Czechia | 5162.07(4493.14-6030.44) | 795.62(692.52-929.46) |  | 4581.86(3906.91-5356.63) | 815.18(695.10-953.03) |  | -11.24(-18.21--4.04) | 0.10(0.00-0.19) |
| C么te d'Ivoire | 15617.46(13253.25-18717.59) | 675.95(573.62-810.13) |  | 28520.71(24083.71-34065.03) | 654.99(553.09-782.31) |  | 82.62(68.85-95.85) | -0.14(-0.17--0.11) |
| Democratic People's Republic of Korea | 12561.71(10857.76-14406.08) | 537.67(464.74-616.61) |  | 7926.53(6848.59-9161.57) | 523.85(452.61-605.48) |  | -36.90(-41.14--32.53) | -0.09(-0.11--0.08) |
| Democratic Republic of the Congo | 54031.15(45626.52-66153.16) | 740.60(625.40-906.75) |  | 93568.45(78400.35-113476.80) | 690.00(578.14-836.80) |  | 73.18(58.06-88.69) | -0.22(-0.26--0.19) |
| Denmark | 2323.70(2107.65-2531.81) | 802.37(727.77-874.23) |  | 2246.28(1969.50-2531.97) | 722.99(633.90-814.94) |  | -3.33(-11.20-5.56) | -0.48(-0.61--0.35) |
| Djibouti | 351.09(302.38-412.60) | 543.25(467.87-638.42) |  | 756.28(647.84-888.77) | 519.08(444.65-610.02) |  | 115.41(99.32-130.46) | -0.14(-0.18--0.10) |
| Dominica | 41.39(36.14-46.91) | 477.87(417.33-541.65) |  | 16.68(14.56-18.93) | 479.65(418.50-544.29) |  | -59.69(-61.82--57.32) | -0.03(-0.06--0.01) |
| Dominican Republic | 4813.00(4234.74-5495.82) | 483.31(425.24-551.87) |  | 4886.31(4294.10-5547.04) | 472.96(415.64-536.92) |  | 1.52(-3.83-6.84) | -0.08(-0.09--0.06) |
| Ecuador | 6891.69(6064.44-7727.21) | 512.18(450.70-574.27) |  | 8904.46(7799.54-10068.87) | 536.05(469.53-606.15) |  | 29.21(22.29-35.91) | 0.19(0.09-0.28) |
| Egypt | 53045.94(46329.06-61551.49) | 621.67(542.95-721.35) |  | 82463.73(72419.98-94248.00) | 632.52(555.48-722.90) |  | 55.46(46.63-66.38) | 0.10(0.05-0.15) |
| El Salvador | 4518.31(3925.87-5133.63) | 586.73(509.80-666.63) |  | 3473.32(2998.28-3974.90) | 578.19(499.11-661.68) |  | -23.13(-27.70--18.51) | -0.01(-0.03-0.01) |
| Equatorial Guinea | 587.85(490.87-718.66) | 714.62(596.73-873.65) |  | 1142.00(982.11-1361.49) | 609.59(524.24-726.75) |  | 94.27(78.59-110.84) | -0.58(-0.61--0.54) |
| Eritrea | 3862.59(3296.25-4586.75) | 619.51(528.67-735.65) |  | 5346.42(4601.50-6372.03) | 582.38(501.23-694.09) |  | 38.42(28.18-49.11) | -0.16(-0.18--0.15) |
| Estonia | 985.77(838.61-1147.04) | 817.35(695.33-951.07) |  | 564.32(472.31-662.12) | 815.79(682.78-957.16) |  | -42.75(-46.70--37.96) | -0.06(-0.09--0.02) |
| Eswatini | 1005.12(859.60-1197.68) | 691.21(591.13-823.63) |  | 962.86(827.59-1134.49) | 685.72(589.38-807.95) |  | -4.20(-10.57-1.62) | -0.05(-0.08--0.02) |
| Ethiopia | 63395.67(53762.36-75188.56) | 657.68(557.74-780.02) |  | 93105.90(79352.83-109852.57) | 583.18(497.04-688.08) |  | 46.86(40.89-52.32) | -0.42(-0.44--0.41) |
| Fiji | 497.81(433.15-567.09) | 528.24(459.63-601.75) |  | 483.62(417.38-555.37) | 531.19(458.43-610.00) |  | -2.85(-8.14-2.18) | 0.00(-0.02-0.02) |
| Finland | 2497.26(2181.34-2854.01) | 798.67(697.64-912.77) |  | 1850.22(1615.47-2105.89) | 762.04(665.35-867.34) |  | -25.91(-33.31--19.70) | -0.21(-0.30--0.12) |
| France | 35651.05(31245.46-40669.21) | 916.63(803.35-1045.65) |  | 30481.94(26285.19-34648.38) | 862.55(743.80-980.45) |  | -14.50(-19.23--9.15) | 0.00(-0.06-0.06) |
| Gabon | 940.45(804.76-1130.82) | 602.12(515.25-724.00) |  | 1212.06(1040.69-1422.31) | 567.61(487.35-666.07) |  | 28.88(18.94-39.03) | -0.19(-0.22--0.17) |
| Gambia | 1224.02(1045.75-1445.55) | 659.17(563.17-778.47) |  | 2301.95(1950.74-2694.83) | 643.74(545.53-753.61) |  | 88.07(74.04-102.44) | -0.11(-0.13--0.09) |
| Georgia | 3484.23(3010.43-4076.33) | 743.60(642.48-869.96) |  | 1935.02(1699.59-2182.18) | 795.34(698.58-896.93) |  | -44.46(-49.85--39.18) | 0.41(0.31-0.51) |
| Germany | 32421.38(28868.56-36039.63) | 723.82(644.50-804.60) |  | 29766.34(26125.50-33733.37) | 736.16(646.12-834.27) |  | -8.19(-14.29--1.43) | 0.11(-0.06-0.29) |
| Ghana | 18491.16(15727.40-21837.23) | 702.01(597.09-829.05) |  | 31935.42(27233.99-37430.45) | 688.24(586.92-806.67) |  | 72.71(58.22-84.93) | -0.01(-0.03-0.01) |
| Greece | 3266.46(2874.19-3700.04) | 588.58(517.90-666.71) |  | 2611.11(2306.89-2930.03) | 617.85(545.86-693.31) |  | -20.06(-24.19--14.79) | 0.30(0.19-0.41) |
| Greenland | 33.01(28.71-39.12) | 599.46(521.39-710.44) |  | 23.18(20.02-27.07) | 573.51(495.41-669.73) |  | -29.78(-35.17--24.79) | -0.17(-0.21--0.12) |
| Grenada | 64.00(55.80-72.56) | 532.91(464.63-604.20) |  | 37.57(32.59-42.60) | 547.06(474.58-620.32) |  | -41.30(-44.11--38.26) | 0.04(-0.01-0.09) |
| Guam | 73.84(64.86-84.30) | 456.58(401.06-521.23) |  | 56.70(49.76-64.68) | 443.73(389.37-506.17) |  | -23.21(-26.94--19.23) | -0.11(-0.15--0.07) |
| Guatemala | 8741.33(7526.18-10444.41) | 569.83(490.62-680.85) |  | 8931.41(7790.66-10303.01) | 572.80(499.64-660.76) |  | 2.17(-6.67-11.91) | 0.09(0.06-0.13) |
| Guinea | 9070.24(7469.42-11208.83) | 781.55(643.62-965.83) |  | 16600.37(13863.61-19706.45) | 737.64(616.03-875.66) |  | 83.02(69.62-99.59) | -0.12(-0.14--0.10) |
| Guinea-Bissau | 1496.49(1253.09-1810.60) | 795.68(666.27-962.69) |  | 2514.92(2126.39-3035.86) | 756.01(639.22-912.61) |  | 68.05(55.81-79.41) | -0.18(-0.21--0.15) |
| Guyana | 579.35(513.28-662.32) | 513.60(455.03-587.16) |  | 401.78(351.22-456.62) | 539.21(471.36-612.81) |  | -30.65(-35.02--26.82) | 0.19(0.17-0.22) |
| Haiti | 6725.03(5835.70-7780.39) | 634.02(550.17-733.51) |  | 9835.37(8525.07-11501.79) | 626.52(543.05-732.67) |  | 46.25(36.77-55.88) | -0.05(-0.07--0.03) |
| Honduras | 5033.87(4369.88-5792.07) | 611.65(530.97-703.77) |  | 6615.93(5767.74-7623.74) | 603.91(526.48-695.90) |  | 31.43(23.83-38.60) | -0.06(-0.08--0.04) |
| Hungary | 5107.47(4332.82-5952.37) | 824.88(699.77-961.33) |  | 3762.99(3213.85-4366.35) | 828.11(707.26-960.89) |  | -26.32(-31.14--21.22) | -0.12(-0.20--0.04) |
| Iceland | 120.55(106.90-134.71) | 570.60(505.99-637.62) |  | 126.41(112.07-141.28) | 575.32(510.04-643.01) |  | 4.86(-0.46-10.11) | 0.08(0.05-0.12) |
| India | 771142.93(658776.26-908684.64) | 663.38(566.72-781.70) |  | 725280.90(623693.65-841748.04) | 651.43(560.19-756.04) |  | -5.95(-8.77--2.74) | 0.12(0.04-0.20) |
| Indonesia | 127580.54(109654.31-149148.80) | 571.58(491.27-668.21) |  | 121509.79(104704.68-140950.86) | 554.67(477.96-643.42) |  | -4.76(-6.97--2.59) | -0.16(-0.19--0.13) |
| Iran (Islamic Republic of) | 56799.84(50195.51-63756.72) | 647.50(572.22-726.81) |  | 40766.87(36073.83-45815.60) | 662.32(586.08-744.35) |  | -28.23(-29.61--26.51) | 0.08(0.05-0.11) |
| Iraq | 20409.22(17752.68-23468.00) | 649.41(564.88-746.74) |  | 27976.81(24401.84-31828.02) | 651.62(568.35-741.32) |  | 37.08(29.30-44.76) | -0.05(-0.08--0.02) |
| Ireland | 1745.76(1598.67-1907.93) | 604.11(553.21-660.23) |  | 1998.48(1772.32-2233.88) | 669.73(593.94-748.62) |  | 14.48(7.56-22.01) | 0.32(0.17-0.46) |
| Israel | 3016.42(2630.42-3433.97) | 584.17(509.41-665.03) |  | 5507.04(4828.19-6228.99) | 599.72(525.79-678.34) |  | 82.57(73.90-92.82) | 0.09(0.05-0.13) |
| Italy | 18853.14(16762.90-21055.26) | 686.63(610.51-766.84) |  | 14318.89(12660.90-15983.46) | 659.81(583.41-736.52) |  | -24.05(-26.12--22.12) | -0.03(-0.26-0.20) |
| Jamaica | 1414.82(1223.60-1612.88) | 507.09(438.55-578.07) |  | 928.06(801.19-1064.27) | 542.15(468.04-621.72) |  | -34.40(-37.47--31.40) | 0.18(0.14-0.22) |
| Japan | 48227.03(42907.80-53807.89) | 724.31(644.43-808.13) |  | 32920.40(29242.67-36648.59) | 717.93(637.73-799.24) |  | -31.74(-33.54--30.01) | -0.05(-0.08--0.02) |
| Jordan | 4064.38(3502.23-4649.77) | 677.16(583.50-774.69) |  | 7628.37(6573.80-8775.06) | 695.19(599.08-799.68) |  | 87.69(78.36-100.60) | 0.05(0.04-0.06) |
| Kazakhstan | 18046.29(15289.53-21560.00) | 958.43(812.02-1145.04) |  | 18893.11(15892.01-22334.71) | 969.65(815.63-1146.29) |  | 4.69(-2.61-13.31) | 0.02(-0.03-0.06) |
| Kenya | 23141.36(20414.17-26079.31) | 539.05(475.52-607.49) |  | 31814.63(27925.73-35975.45) | 534.68(469.33-604.61) |  | 37.48(35.66-39.50) | -0.02(-0.03--0.02) |
| Kiribati | 73.28(63.31-86.40) | 622.31(537.61-733.66) |  | 85.63(74.14-99.56) | 599.13(518.72-696.60) |  | 16.85(10.23-23.51) | -0.09(-0.11--0.07) |
| Kuwait | 1211.20(1042.74-1383.81) | 592.42(510.02-676.85) |  | 1872.18(1610.02-2158.16) | 706.45(607.52-814.36) |  | 54.57(45.67-63.29) | 0.55(0.46-0.64) |
| Kyrgyzstan | 6226.87(5250.85-7542.07) | 968.80(816.94-1173.42) |  | 7876.06(6648.28-9375.32) | 992.07(837.42-1180.92) |  | 26.49(17.73-35.57) | 0.13(0.09-0.17) |
| Lao People's Democratic Republic | 4977.46(4241.67-5861.57) | 700.11(596.62-824.46) |  | 5373.56(4643.64-6263.38) | 647.78(559.79-755.05) |  | 7.96(1.07-16.40) | -0.27(-0.29--0.24) |
| Latvia | 1681.98(1425.12-1967.52) | 830.05(703.29-970.96) |  | 763.90(646.40-891.83) | 815.14(689.76-951.66) |  | -54.58(-57.89--51.03) | 0.01(-0.02-0.03) |
| Lebanon | 2452.64(2117.84-2796.72) | 629.51(543.58-717.82) |  | 2592.62(2247.17-2950.70) | 638.73(553.63-726.95) |  | 5.71(0.81-11.74) | 0.01(-0.02-0.04) |
| Lesotho | 1719.89(1480.67-2054.38) | 698.28(601.16-834.08) |  | 1457.20(1259.68-1732.61) | 715.32(618.35-850.51) |  | -15.27(-19.87--9.87) | 0.07(0.05-0.08) |
| Liberia | 3395.58(2866.25-4176.80) | 731.14(617.16-899.35) |  | 5154.83(4331.32-6120.80) | 672.61(565.16-798.66) |  | 51.81(39.87-66.68) | -0.30(-0.33--0.26) |
| Libya | 3510.44(3080.37-3935.15) | 551.16(483.63-617.84) |  | 2447.12(2122.74-2792.52) | 578.41(501.74-660.05) |  | -30.29(-33.97--26.51) | 0.11(0.09-0.12) |
| Lithuania | 2421.71(2056.77-2854.11) | 837.74(711.50-987.32) |  | 1111.29(939.49-1308.04) | 844.49(713.93-993.99) |  | -54.11(-57.13--50.50) | 0.06(0.02-0.10) |
| Luxembourg | 142.03(125.12-159.36) | 620.32(546.45-695.99) |  | 210.80(185.30-235.79) | 637.53(560.41-713.11) |  | 48.41(41.40-56.53) | 0.15(0.14-0.17) |
| Madagascar | 13180.97(11237.23-15835.11) | 611.48(521.30-734.60) |  | 23559.22(20106.96-28216.77) | 576.29(491.85-690.22) |  | 78.74(65.95-91.97) | -0.15(-0.17--0.14) |
| Malawi | 12078.93(10153.10-14308.57) | 636.16(534.73-753.59) |  | 15861.17(13605.29-19268.60) | 582.26(499.45-707.35) |  | 31.31(18.82-41.14) | -0.30(-0.33--0.28) |
| Malaysia | 11997.62(10365.13-13738.07) | 503.61(435.08-576.66) |  | 12394.48(10651.78-14191.34) | 504.17(433.28-577.26) |  | 3.31(-3.76-10.10) | -0.01(-0.04-0.01) |
| Maldives | 224.57(190.97-262.18) | 537.48(457.07-627.49) |  | 158.19(137.68-181.47) | 498.10(433.51-571.41) |  | -29.56(-34.57--24.27) | -0.23(-0.25--0.21) |
| Mali | 13865.57(11675.24-17047.03) | 801.86(675.19-985.85) |  | 34344.52(28858.19-40925.16) | 749.82(630.04-893.49) |  | 147.70(123.77-173.04) | -0.20(-0.23--0.17) |
| Malta | 159.19(140.62-177.94) | 563.65(497.88-630.02) |  | 133.85(118.18-149.94) | 608.34(537.12-681.48) |  | -15.92(-20.70--11.10) | 0.51(0.40-0.62) |
| Marshall Islands | 39.92(34.48-46.35) | 536.81(463.55-623.20) |  | 30.41(26.58-34.81) | 536.00(468.56-613.62) |  | -23.83(-28.31--19.26) | -0.05(-0.07--0.03) |
| Mauritania | 2410.58(2040.96-2887.34) | 649.85(550.20-778.37) |  | 4007.02(3408.42-4761.37) | 609.89(518.78-724.71) |  | 66.23(53.07-80.29) | -0.18(-0.19--0.16) |
| Mauritius | 544.23(466.95-625.00) | 516.01(442.73-592.59) |  | 315.49(271.23-360.05) | 490.69(421.84-559.99) |  | -42.03(-44.92--39.03) | -0.19(-0.27--0.12) |
| Mexico | 71267.70(63461.91-80048.48) | 604.06(537.90-678.49) |  | 62152.94(55226.38-69573.38) | 629.26(559.13-704.39) |  | -12.79(-15.16--10.37) | 0.15(0.09-0.22) |
| Micronesia (Federated States of) | 84.43(73.02-97.59) | 539.15(466.33-623.22) |  | 50.34(43.68-58.79) | 531.59(461.19-620.75) |  | -40.37(-43.65--37.17) | -0.10(-0.13--0.08) |
| Monaco | 7.35(6.45-8.27) | 627.90(551.14-706.09) |  | 10.38(9.13-11.65) | 641.61(564.26-719.87) |  | 41.21(34.30-48.58) | 0.10(0.08-0.13) |
| Mongolia | 3945.23(3313.68-4684.69) | 1161.98(975.97-1379.77) |  | 4345.98(3652.95-5205.04) | 1112.37(934.99-1332.25) |  | 10.16(0.78-20.58) | -0.13(-0.17--0.08) |
| Montenegro | 332.72(281.00-383.94) | 634.19(535.60-731.81) |  | 241.32(205.12-287.20) | 666.79(566.75-793.57) |  | -27.47(-32.51--21.68) | 0.14(0.10-0.18) |
| Morocco | 20664.34(18104.93-23987.82) | 580.40(508.51-673.74) |  | 18984.41(16603.76-21818.79) | 583.99(510.76-671.18) |  | -8.13(-14.22--1.45) | 0.01(-0.01-0.04) |
| Mozambique | 15058.33(12747.28-17884.87) | 625.09(529.15-742.42) |  | 30528.87(26294.38-35831.41) | 589.45(507.69-691.83) |  | 102.74(87.88-118.04) | -0.19(-0.21--0.17) |
| Myanmar | 33605.49(28526.83-39547.96) | 666.48(565.76-784.34) |  | 32710.30(27975.87-39007.31) | 625.87(535.28-746.35) |  | -2.66(-10.34-4.82) | -0.26(-0.29--0.23) |
| Namibia | 1533.05(1309.60-1826.21) | 677.27(578.55-806.78) |  | 1838.68(1590.36-2139.08) | 660.32(571.14-768.20) |  | 19.94(11.21-30.04) | -0.10(-0.12--0.08) |
| Nauru | 9.21(8.03-10.76) | 563.54(491.22-658.66) |  | 7.72(6.65-8.89) | 551.98(475.49-635.99) |  | -16.20(-21.80--11.21) | -0.11(-0.14--0.07) |
| Nepal | 19986.18(16796.85-23831.92) | 606.86(510.02-723.63) |  | 17827.17(14963.13-21571.44) | 573.89(481.69-694.42) |  | -10.80(-17.99--3.19) | -0.20(-0.27--0.13) |
| Netherlands | 6193.25(5699.74-6742.03) | 661.45(608.74-720.06) |  | 5047.06(4440.36-5670.27) | 586.03(515.59-658.39) |  | -18.51(-24.98--11.08) | -0.24(-0.32--0.17) |
| New Zealand | 1749.06(1520.22-2013.99) | 625.45(543.62-720.19) |  | 1994.75(1740.47-2269.39) | 638.12(556.77-725.97) |  | 14.05(7.34-21.48) | 0.14(0.07-0.21) |
| Nicaragua | 3541.00(3070.53-4063.16) | 533.68(462.77-612.38) |  | 3479.68(3036.83-3961.39) | 534.90(466.82-608.94) |  | -1.73(-6.77-4.30) | 0.02(0.01-0.04) |
| Niger | 14088.74(11843.50-17247.78) | 835.14(702.05-1022.40) |  | 39870.12(33381.09-48294.11) | 782.33(655.00-947.62) |  | 182.99(156.95-209.79) | -0.26(-0.29--0.23) |
| Nigeria | 115818.02(97905.91-138414.90) | 728.64(615.95-870.80) |  | 256091.70(218233.95-303237.79) | 689.94(587.95-816.96) |  | 121.12(116.68-125.20) | -0.20(-0.22--0.18) |
| Niue | 1.24(1.08-1.42) | 485.93(422.01-558.63) |  | 0.57(0.49-0.66) | 484.44(416.90-558.52) |  | -54.10(-56.44--51.62) | -0.06(-0.08--0.04) |
| North Macedonia | 1205.36(1036.70-1426.36) | 707.62(608.60-837.36) |  | 710.21(601.15-837.26) | 706.55(598.06-832.95) |  | -41.08(-45.41--36.45) | -0.02(-0.04--0.00) |
| Northern Mariana Islands | 22.63(19.64-25.66) | 476.27(413.29-539.97) |  | 14.56(12.69-16.48) | 453.87(395.57-514.02) |  | -35.69(-38.76--32.21) | -0.18(-0.21--0.15) |
| Norway | 1826.89(1624.77-2038.71) | 662.27(589.00-739.06) |  | 1873.96(1660.82-2108.36) | 666.61(590.79-749.99) |  | 2.58(-0.55-5.58) | -0.15(-0.30--0.01) |
| Oman | 1999.00(1774.21-2222.26) | 610.05(541.45-678.19) |  | 2501.98(2189.98-2821.73) | 590.06(516.48-665.47) |  | 25.16(15.33-34.28) | -0.18(-0.23--0.14) |
| Pakistan | 124163.19(103819.56-148816.70) | 672.55(562.36-806.09) |  | 202804.62(169349.05-243125.48) | 682.25(569.70-817.89) |  | 63.34(55.60-71.43) | 0.08(0.04-0.11) |
| Palau | 7.28(6.33-8.32) | 493.23(428.33-563.17) |  | 4.59(3.99-5.33) | 484.82(420.96-562.80) |  | -36.97(-40.43--33.19) | -0.10(-0.12--0.07) |
| Palestine | 2426.48(2102.34-2762.75) | 622.18(539.07-708.41) |  | 3817.23(3323.93-4336.74) | 622.96(542.45-707.74) |  | 57.32(48.36-66.61) | -0.02(-0.04--0.00) |
| Panama | 1603.16(1385.11-1825.48) | 561.42(485.06-639.27) |  | 2130.45(1839.91-2431.97) | 573.88(495.62-655.11) |  | 32.89(25.86-40.03) | 0.12(0.11-0.14) |
| Papua New Guinea | 3923.04(3363.72-4603.96) | 605.69(519.34-710.82) |  | 9254.27(7887.75-10866.15) | 608.31(518.48-714.26) |  | 135.90(120.78-153.72) | 0.02(-0.00-0.04) |
| Paraguay | 3221.39(2810.71-3674.75) | 522.09(455.53-595.57) |  | 3620.76(3151.54-4098.57) | 556.98(484.80-630.48) |  | 12.40(7.50-18.18) | 0.24(0.22-0.26) |
| Peru | 14767.78(12932.04-16670.45) | 504.71(441.97-569.74) |  | 16651.67(14451.16-18833.96) | 504.52(437.85-570.64) |  | 12.76(6.65-18.16) | 0.00(-0.02-0.02) |
| Philippines | 52908.07(45929.51-61011.69) | 571.91(496.47-659.50) |  | 63951.78(55896.56-73267.67) | 570.32(498.48-653.39) |  | 20.87(19.26-22.49) | -0.01(-0.02-0.01) |
| Poland | 23047.03(19965.46-26208.17) | 789.60(684.03-897.90) |  | 15723.87(13634.66-17771.48) | 835.45(724.45-944.25) |  | -31.77(-34.54--28.56) | -0.07(-0.21-0.06) |
| Portugal | 3237.83(2818.09-3696.67) | 559.77(487.21-639.10) |  | 2561.54(2221.72-2883.92) | 602.25(522.36-678.05) |  | -20.89(-26.24--14.96) | 0.32(0.29-0.35) |
| Puerto Rico | 1745.27(1493.06-2011.82) | 546.77(467.76-630.28) |  | 589.39(503.05-682.36) | 560.68(478.54-649.12) |  | -66.23(-67.99--64.33) | -0.03(-0.06--0.00) |
| Qatar | 314.36(270.80-357.07) | 618.77(533.02-702.83) |  | 1205.88(1045.72-1376.35) | 654.30(567.40-746.79) |  | 283.59(263.39-300.68) | 0.26(0.22-0.29) |
| Republic of Korea | 24431.74(21574.88-28186.28) | 736.10(650.02-849.22) |  | 10390.55(9011.93-11787.89) | 670.40(581.45-760.56) |  | -57.47(-59.98--54.84) | -0.37(-0.41--0.34) |
| Republic of Moldova | 3574.87(3033.56-4194.93) | 829.72(704.08-973.63) |  | 1271.30(1077.14-1474.52) | 824.33(698.43-956.10) |  | -64.44(-66.93--61.68) | 0.05(0.01-0.08) |
| Romania | 13247.84(11256.90-15516.91) | 748.66(636.15-876.89) |  | 7202.42(6166.54-8411.74) | 767.97(657.52-896.92) |  | -45.63(-49.50--41.39) | 0.09(0.05-0.12) |
| Russian Federation | 89293.41(76299.49-102925.66) | 768.16(656.38-885.44) |  | 57870.60(49394.22-66863.71) | 760.39(649.01-878.55) |  | -35.19(-36.29--34.04) | -0.04(-0.08--0.00) |
| Rwanda | 8451.89(7179.66-9964.38) | 627.06(532.67-739.27) |  | 10009.57(8534.66-11763.83) | 572.48(488.12-672.81) |  | 18.43(10.64-28.67) | -0.37(-0.41--0.32) |
| Saint Kitts and Nevis | 23.38(20.54-26.57) | 498.98(438.35-567.13) |  | 14.99(13.09-17.07) | 491.09(428.87-559.31) |  | -35.91(-39.35--32.47) | -0.05(-0.09--0.01) |
| Saint Lucia | 92.65(80.10-105.46) | 524.67(453.63-597.20) |  | 46.40(40.26-52.94) | 525.26(455.75-599.36) |  | -49.92(-52.56--47.30) | -0.02(-0.05-0.01) |
| Saint Vincent and the Grenadines | 68.68(59.79-78.17) | 537.49(467.93-611.74) |  | 38.57(33.46-44.08) | 535.95(464.90-612.50) |  | -43.84(-46.91--40.91) | -0.01(-0.07-0.04) |
| Samoa | 127.33(109.91-147.76) | 499.53(431.18-579.65) |  | 144.64(124.85-166.94) | 494.58(426.93-570.85) |  | 13.59(7.46-20.48) | -0.06(-0.08--0.03) |
| San Marino | 6.72(5.94-7.58) | 566.70(500.97-639.15) |  | 7.07(6.21-7.98) | 584.77(513.36-659.86) |  | 5.16(-0.10-10.62) | 0.14(0.11-0.17) |
| Sao Tome and Principe | 125.22(106.87-146.98) | 611.42(521.81-717.68) |  | 148.12(126.08-174.73) | 593.81(505.47-700.47) |  | 18.29(10.11-26.69) | -0.11(-0.13--0.08) |
| Saudi Arabia | 14554.60(12926.65-16242.38) | 602.00(534.67-671.81) |  | 13764.03(11947.73-15449.52) | 565.70(491.05-634.97) |  | -5.43(-12.34-1.58) | -0.15(-0.20--0.10) |
| Senegal | 10037.69(8450.36-11788.05) | 685.14(576.79-804.62) |  | 15115.50(12798.23-17801.88) | 665.46(563.44-783.72) |  | 50.59(40.38-63.33) | -0.08(-0.10--0.06) |
| Serbia | 4811.87(4098.20-5660.71) | 706.88(602.04-831.57) |  | 2597.86(2227.73-3019.14) | 704.68(604.28-818.95) |  | -46.01(-50.15--42.06) | -0.08(-0.11--0.05) |
| Seychelles | 42.56(36.73-49.07) | 528.15(455.85-608.97) |  | 41.83(35.84-48.31) | 531.18(455.10-613.48) |  | -1.72(-6.76-3.60) | 0.04(0.02-0.07) |
| Sierra Leone | 6012.73(5063.32-7321.20) | 777.04(654.35-946.14) |  | 9746.57(8251.39-11904.14) | 725.76(614.42-886.42) |  | 62.10(49.74-76.32) | -0.19(-0.21--0.17) |
| Singapore | 1313.61(1138.90-1494.64) | 643.79(558.16-732.51) |  | 1753.20(1512.89-2013.55) | 613.16(529.11-704.21) |  | 33.46(26.04-40.97) | -0.23(-0.26--0.20) |
| Slovakia | 3103.30(2670.32-3608.70) | 758.43(652.61-881.95) |  | 2209.60(1901.75-2565.10) | 772.23(664.64-896.48) |  | -28.80(-33.27--23.89) | 0.06(0.04-0.08) |
| Slovenia | 910.46(782.81-1059.53) | 740.26(636.47-861.46) |  | 745.01(634.42-868.34) | 761.00(648.04-886.98) |  | -18.17(-23.62--13.00) | 0.04(0.03-0.06) |
| Solomon Islands | 349.79(302.79-406.77) | 586.35(507.57-681.88) |  | 551.08(479.27-635.65) | 578.03(502.71-666.74) |  | 57.55(47.11-66.97) | -0.08(-0.09--0.06) |
| Somalia | 10393.88(8846.66-12392.62) | 672.73(572.59-802.09) |  | 27461.75(23250.96-32905.55) | 665.08(563.10-796.92) |  | 164.21(146.78-183.87) | -0.07(-0.09--0.05) |
| South Africa | 30275.98(26580.20-35134.13) | 620.63(544.87-720.22) |  | 30599.82(26850.82-35084.84) | 616.77(541.20-707.17) |  | 1.07(-2.74-4.97) | -0.01(-0.03-0.02) |
| South Sudan | 6525.96(5539.95-7783.06) | 641.18(544.30-764.69) |  | 9730.33(8314.56-11459.33) | 622.81(532.19-733.47) |  | 49.10(39.97-60.55) | -0.12(-0.14--0.11) |
| Spain | 12911.15(11644.62-14222.44) | 619.91(559.10-682.87) |  | 12084.22(10800.64-13305.89) | 656.30(586.59-722.65) |  | -6.40(-11.49--1.47) | -0.00(-0.09-0.08) |
| Sri Lanka | 9080.99(7857.29-10560.98) | 513.68(444.46-597.39) |  | 8150.29(6997.05-9334.33) | 520.58(446.92-596.21) |  | -10.25(-15.53--4.61) | 0.12(0.09-0.14) |
| Sudan | 25169.84(21941.58-29759.58) | 723.09(630.34-854.94) |  | 40153.88(34891.61-46399.77) | 712.07(618.75-822.83) |  | 59.53(47.77-73.00) | -0.05(-0.06--0.03) |
| Suriname | 213.68(188.92-238.75) | 487.81(431.28-545.05) |  | 222.50(194.87-254.07) | 499.59(437.54-570.47) |  | 4.13(-1.22-9.45) | 0.03(-0.00-0.06) |
| Sweden | 4540.43(3923.94-5207.43) | 806.39(696.90-924.85) |  | 4028.16(3506.23-4582.93) | 690.71(601.21-785.83) |  | -11.28(-16.46--6.03) | -0.35(-0.51--0.19) |
| Switzerland | 3148.74(2907.25-3406.83) | 790.51(729.88-855.30) |  | 3078.89(2710.86-3439.31) | 696.59(613.32-778.13) |  | -2.22(-9.04-4.92) | -0.34(-0.42--0.26) |
| Syrian Arab Republic | 12779.76(11046.49-14454.23) | 592.49(512.14-670.13) |  | 6596.98(5744.04-7501.24) | 656.37(571.51-746.34) |  | -48.38(-51.84--45.30) | 0.41(0.37-0.45) |
| Taiwan (Province of China) | 7685.10(6632.98-8735.74) | 478.39(412.90-543.79) |  | 4035.79(3501.45-4590.74) | 452.32(392.44-514.52) |  | -47.49(-50.10--44.82) | -0.18(-0.19--0.16) |
| Tajikistan | 10167.03(8574.56-12204.70) | 1075.26(906.84-1290.77) |  | 15062.42(12690.69-18169.07) | 1125.33(948.14-1357.44) |  | 48.15(36.90-61.61) | 0.15(0.12-0.18) |
| Thailand | 27567.31(23904.29-31881.51) | 530.11(459.67-613.07) |  | 14715.16(12802.82-16798.39) | 520.52(452.87-594.21) |  | -46.62(-49.91--43.24) | -0.06(-0.08--0.04) |
| Timor-Leste | 909.85(772.74-1072.07) | 649.66(551.76-765.49) |  | 1133.66(970.53-1324.37) | 613.29(525.04-716.46) |  | 24.60(15.10-33.64) | -0.27(-0.32--0.22) |
| Togo | 4755.70(3950.20-5720.69) | 692.95(575.58-833.56) |  | 7825.57(6630.45-9321.39) | 667.42(565.49-794.99) |  | 64.55(51.18-77.49) | -0.12(-0.15--0.10) |
| Tokelau | 1.01(0.88-1.16) | 517.80(451.83-596.76) |  | 0.49(0.42-0.56) | 494.69(427.99-568.92) |  | -51.36(-54.14--48.37) | -0.16(-0.19--0.14) |
| Tonga | 73.56(63.44-84.43) | 481.60(415.31-552.78) |  | 69.26(60.22-79.62) | 480.71(417.94-552.61) |  | -5.85(-10.57-0.32) | -0.01(-0.02--0.00) |
| Trinidad and Tobago | 715.44(620.49-812.01) | 536.64(465.42-609.08) |  | 452.54(388.48-518.87) | 562.06(482.50-644.45) |  | -36.75(-40.09--33.21) | 0.15(0.12-0.18) |
| Tunisia | 6706.29(5795.77-7686.73) | 628.43(543.10-720.30) |  | 5840.74(5031.41-6659.65) | 654.77(564.04-746.57) |  | -12.91(-17.40--8.06) | 0.15(0.12-0.17) |
| Turkey | 5876.80(4953.87-7199.67) | 1003.57(845.96-1229.48) |  | 5572.21(4724.45-6661.06) | 1032.44(875.36-1234.18) |  | -5.18(-13.05-3.95) | 0.09(0.06-0.11) |
| Turkmenistan | 8.64(7.38-10.11) | 572.92(489.47-669.97) |  | 6.97(6.02-8.17) | 543.36(469.16-636.51) |  | -19.32(-24.06--13.62) | -0.19(-0.22--0.15) |
| Tuvalu | 48653.08(41996.49-55302.13) | 699.54(603.83-795.14) |  | 38276.25(33148.56-43526.00) | 689.43(597.07-783.99) |  | -21.33(-25.61--16.80) | 0.01(-0.04-0.07) |
| Uganda | 21190.82(17994.95-24969.34) | 590.03(501.05-695.24) |  | 41499.04(35900.85-48861.23) | 567.10(490.60-667.71) |  | 95.83(81.10-111.89) | -0.15(-0.17--0.13) |
| Ukraine | 31479.25(26811.51-36763.78) | 835.23(711.38-975.44) |  | 14075.70(12044.19-16375.05) | 884.04(756.45-1028.45) |  | -55.29(-58.31--52.08) | 0.23(0.18-0.27) |
| United Arab Emirates | 1355.49(1175.99-1540.53) | 594.65(515.90-675.83) |  | 2656.84(2305.06-3020.71) | 614.05(532.74-698.14) |  | 96.01(85.62-106.56) | 0.12(0.09-0.14) |
| United Kingdom | 17004.47(15328.95-18826.44) | 442.66(399.04-490.09) |  | 19971.16(17682.10-22367.47) | 546.46(483.83-612.03) |  | 17.45(13.19-22.61) | 0.81(0.72-0.90) |
| United Republic of Tanzania | 27199.50(23190.98-31951.78) | 565.71(482.34-664.55) |  | 47275.19(40764.36-55620.24) | 534.41(460.81-628.75) |  | 73.81(63.04-86.26) | -0.18(-0.20--0.16) |
| United States of America | 116461.07(100969.26-132921.73) | 589.65(511.22-673.00) |  | 109326.76(96083.13-124797.59) | 587.96(516.74-671.17) |  | -6.13(-10.50--1.29) | -0.01(-0.09-0.06) |
| United States Virgin Islands | 59.36(51.35-68.05) | 539.20(466.48-618.20) |  | 20.87(18.10-23.69) | 532.41(461.75-604.37) |  | -64.85(-66.66--63.23) | -0.09(-0.12--0.07) |
| Uruguay | 1539.95(1327.19-1782.82) | 564.42(486.44-653.44) |  | 1180.03(1015.73-1371.00) | 608.26(523.57-706.70) |  | -23.37(-28.79--17.40) | 0.26(0.23-0.30) |
| Uzbekistan | 33800.07(28503.00-40863.77) | 1002.08(845.03-1211.50) |  | 40897.33(34738.19-48510.07) | 1066.36(905.76-1264.85) |  | 21.00(11.85-30.15) | 0.19(0.16-0.22) |
| Vanuatu | 146.73(126.47-171.53) | 542.63(467.72-634.38) |  | 227.67(196.22-263.23) | 540.13(465.52-624.48) |  | 55.17(45.50-65.60) | -0.05(-0.06--0.03) |
| Venezuela (Bolivarian Republic of) | 13582.09(11805.30-15496.40) | 536.67(466.47-612.31) |  | 12757.22(11017.87-14538.28) | 583.87(504.27-665.39) |  | -6.07(-11.09--0.08) | 0.33(0.27-0.39) |
| Viet Nam | 47455.80(40702.43-55455.48) | 504.35(432.57-589.37) |  | 39699.03(34408.19-45670.87) | 487.60(422.61-560.94) |  | -16.35(-22.23--11.07) | -0.09(-0.11--0.08) |
| Yemen | 19499.56(16959.62-22515.04) | 702.29(610.81-810.89) |  | 33793.15(29827.16-38922.56) | 719.42(634.98-828.61) |  | 73.30(63.63-85.39) | 0.04(0.00-0.07) |
| Zambia | 9200.62(7929.66-10775.62) | 609.65(525.43-714.01) |  | 16380.69(14088.67-19711.07) | 560.02(481.66-673.87) |  | 78.04(67.96-90.59) | -0.28(-0.33--0.24) |
| Zimbabwe | 11678.04(10052.21-13860.34) | 662.20(570.00-785.94) |  | 15153.62(12970.98-17887.19) | 685.77(587.00-809.48) |  | 29.76(20.97-39.84) | 0.15(0.12-0.19) |
